# *Cryptosporidium* lysyl-tRNA synthetase inhibitors define the interplay between solubility and permeability required to achieve efficacy

**DOI:** 10.1126/scitranslmed.adm8631

**Published:** 2024-10-23

**Authors:** Nicola Caldwell, Caroline Peet, Peter Miller, Beatrice L. Colon, Malcolm G. Taylor, Mattia Cocco, Alice Dawson, Iva Lukac, Jose E. Teixeira, Lee Robinson, Laura Frame, Simona Seizova, Sebastian Damerow, Fabio Tamaki, John Post, Jennifer Riley, Nicole Mutter, Jack C. Hanna, Liam Ferguson, Xiao Hu, Michele Tinti, Barbara Forte, Neil R. Norcross, Peter S. Campbell, Nina Svensen, Flora C. Caldwell, Chimed Jansen, Vincent Postis, Kevin D. Read, Christopher D. Huston, Ian H. Gilbert, Beatriz Baragaña, Mattie C. Pawlowic

**Affiliations:** 1 Wellcome Centre for Anti-Infectives Research, Division of Biological Chemistry and Drug Discovery, School of Life Sciences, University of Dundee, Dundee, DD1 5EH, UK; 2 Department of Medicine, University of Vermont, Larner College of Medicine, Burlington, Vermont, 05401, USA

## Abstract

Cryptosporidiosis is a diarrheal disease caused by infection with *Cryptosporidium spp* parasites and is a leading cause of death in malnourished children worldwide. The only approved treatment, nitazoxanide, has limited efficacy in this at-risk patient population. Additional safe therapeutics are urgently required to tackle this unmet medical need. However, development of anti-cryptosporidial drugs is hindered by a lack of understanding of the optimal compound properties required for treatment of this gastrointestinal infection. To address this knowledge gap, a diverse set of potent lysyl-tRNA synthetase inhibitors were profiled to identify optimal physicochemical and pharmacokinetic properties required for efficacy in a chronic mouse model of infection. The results from this comprehensive study illustrated the importance of balancing solubility and permeability to achieve efficacy *in vivo*. Our results establish *in vitro* criteria for solubility and permeability that is predictive of compound efficacy *in vivo* to guide the optimization of anti-cryptosporidial drugs. Two compounds from chemically distinct series (DDD489 and DDD508) were identified as demonstrating superior efficacy and prioritized for further evaluation. Both compounds achieved marked parasite reduction in acute and immunocompromised mouse models as well as in a disease-relevant calf model of infection. Based on this promising data, these compounds have been selected for progression to preclinical safety studies, expanding the portfolio of potential treatments for this neglected infectious disease.

## Introduction

Cryptosporidiosis, a parasitic infection that causes diarrheal disease, is predominantly caused by *Cryptosporidium parvum* (*C. parvum*) and *Cryptosporidium hominis* (*C. hominis*) in humans. An analysis of cause of death in children under the age of five across 195 countries (1980-2017) highlighted that diarrheal disease accounted for 11% of deaths.(1) More recently, cryptosporidiosis has been identified as a leading cause of pathogen-specific diarrhea.(2, 3) In Low- and Middle-Income Countries (LMICs), malnutrition exacerbates the effect of *Cryptosporidium* infection, causing moderate-to-severe diarrhea that can persist. This can cause mortality or other long term effects such as growth stunting and cognitive impairment.(2) Immunocompromised adults, including those with advanced HIV/AIDS or those undergoing organ transplant, are also at increased risk of cryptosporidiosis.(4) Unfortunately, the only approved treatment, nitazoxanide, has limited efficacy in immunocompromised patients and malnourished children.(5) Safe and effective treatments are urgently required to tackle cryptosporidiosis in these highly susceptible patient populations.(6) There is no vaccine for human cryptosporidiosis and preventing exposure to the pathogen is difficult.

Several advanced anti-cryptosporidial compounds have recently emerged.(7) The starting point for these leads originated from phenotypic screens against *Cryptosporidium*, or from target-based anti-malarial drug discovery programs in what is classified as a ‘pathogen-hopping’ strategy.(8) Due to the differences in parasite distribution within the body, considerable optimization of pharmacokinetic (PK) properties is required to adapt anti-malarial compounds for use in cryptosporidiosis. *Cryptosporidium* infection is mainly limited to the small intestine where parasites replicate within epithelial cells, at the brush border amongst the microvilli. Parasite replication occurs inside an intracellular but extracytoplasmic parasitophorous vacuole.(9) This unique location allows parasites to evade the host immune system, and to access nutrients and metabolites from both the intestinal lumen and the host cell. However, this creates a challenge for anti-cryptosporidial drug discovery because compounds must penetrate both intestinal cell membrane and the membrane of the parasitophorous vacuole to reach the parasite.

Due to the specialized location of *Cryptosporidium* parasites, it is not clear whether intestinal or systemic exposure of drug is more desirable for ensuring compounds reach this specialized niche. If systemic exposure is not necessary, restricting compounds to the GI tract could result in increased drug concentration and parasite killing in the gut and a consequent increase in efficacy. One strategy for optimizing drug concentration at the gut wall is to slow down absorption from the small intestine into the systemic circulation by modifying physicochemical properties such as solubility and permeability.(10) The balance of these properties and their relationship with drug efficacy in cryptosporidiosis is not well understood. Compound progression criteria for pre-clinical optimization of new leads for treating systemic infections, such as malaria, have been outlined,(11) but similar guidelines for designing anti-cryptosporidial compounds have not been defined. Ultimately, an understanding of the relationship between *in vitro* physicochemical properties and *in vivo* efficacy is needed to establish criteria to progress the most promising leads along the drug discovery pathway towards the clinic.

We recently described a series of *Plasmodium falciparum* lysyl-tRNA synthetase (*Pf*KRS) inhibitors as potential anti-malarial treatments.(8) By using a pathogen-hopping strategy and exploiting the high sequence identity between *Pf*KRS and *C. parvum* KRS (*Cp*KRS), we demonstrated that an early lead compound from this series (DDD706, Fig. S1A) also inhibited *Cp*KRS and displayed modest inhibition of *C. parvum* growth *in vitro*. Additionally, DDD706 substantially reduced *in vivo* parasite burden in two different mouse models of cryptosporidiosis.(8) This highlighted that *Cp*KRS may also be a valuable target for novel anti-cryptosporidials. Subsequent mode of action studies genetically validated *Cp*KRS as essential for parasite survival and confirmed that DDD706 did indeed target *Cp*KRS.(12)

Despite its utility in validating *Cp*KRS as a drug target for cryptosporidiosis, DDD706 was unsuitable for further progression as toxicity was observed in mice at oral doses greater than 50 mg/kg. Therefore, we initiated a structurally-enabled lead optimization program to develop more advanced *Cp*KRS inhibitors for use as anti-cryptosporidials.(8) Our aim was to identify compound properties that would allow us to select the most promising *Cp*KRS inhibitors for progression. Solubility and permeability are two important factors affecting oral drug absorption and hence determine whether the compound will enter the systemic circulation rapidly, be more slowly absorbed, or be restricted to the small intestine. Therefore, we chose these two properties as the key *in vitro* parameters for investigation. Here we describe the results of our systematic investigation of suitable property profiles to support the development pathway for cryptosporidiosis treatments.

## Results

### A balance between solubility and permeability of anti-cryptosporidial compounds maximizes efficacy in the NOD SCID Gamma mouse model

In the lead optimization phase, we focused on increasing potency and selectivity, and mitigating *in vivo* toxicity. Medicinal chemistry optimization, to be reported separately, delivered two lead chemical series (Fig. S1B) that demonstrated improved potency against *Cp*KRS and selectivity over its human orthologue (*Hs*KRS).

Having optimized potency and selectivity for two series of *Cp*KRS inhibitors (Fig. S1B), the progression of these compounds was stalled by a lack of understanding of the optimal property profile required for successful cryptosporidiosis treatment and no clearly defined compound progression criteria. Therefore, we decided to evaluate a selection of *Cp*KRS inhibitors as tool compounds *in vivo* to fully investigate the relationship between physicochemical properties and efficacy. We selected 14 tool compounds from across the two series to investigate the PK and physicochemical properties required to deliver efficacy in mouse and calf models of infection.

**Figure 1:**
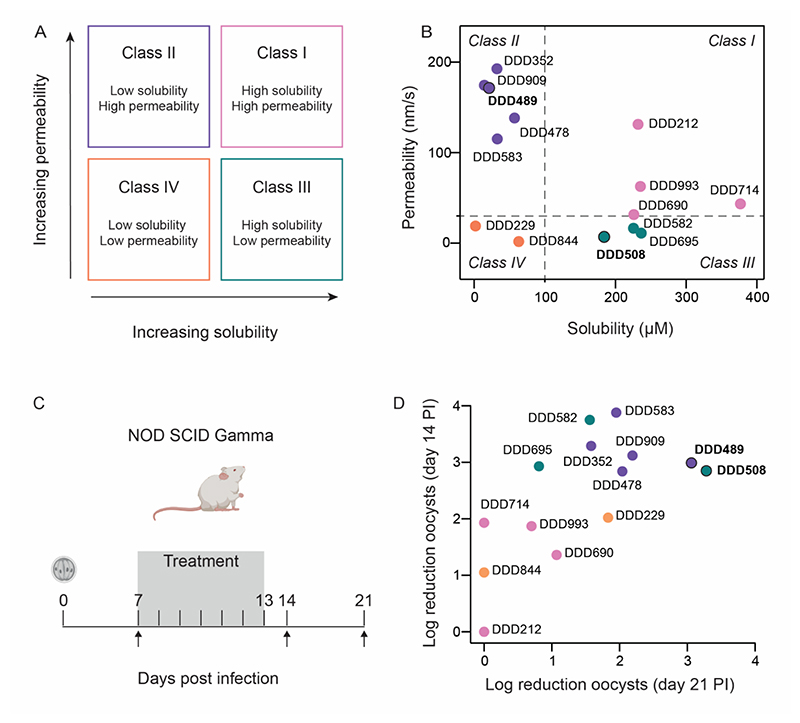
A balance between solubility and permeability maximizes efficacy in a NOD SCID Gamma mouse model of cryptosporidiosis. (**A**) Schematic of Biopharmaceutical Classification System (BCS). (**B**) Aqueous solubility and Parallel Artificial Membrane Permeability Assay (PAMPA) measurements of tool compounds from each BCS class. Data are means of two technical replicates. Solubility and permeability cut-offs were defined at 100 μM and 30 nm/s respectively. Raw data reported for all tool compounds in Table S1. (**C**) NOD SCID Gamma mice were infected with wild type *C. parvum*. Vehicle, positive control, or tool compound was administered via oral gavage starting day 7 post infection (PI) for 7 days (gray box indicates treatment period). Fecal samples were collected on days indicated by arrows. Fecal samples were collected from individual mice (4 animals per treatment group) and analyzed by qPCR to quantify parasite shedding (oocysts/g). (**D**) Log reduction in oocyst shedding measured on day 14 PI vs day 21 PI for tool compounds (colored by BCS class) dosed orally at either 30 or 50 mg/kg twice a day (BID) for 7 days in NOD SCID Gamma mice. Most compounds dosed at 50 mg/kg; DDD212, DDD352, DDD478, DDD583, DDD582 dosed at 30 mg/kg. Mean oocyst shedding was determined for each group of n=4 mice, for all compounds tested. Log reduction in oocyst shedding was calculated as log (mean oocysts on day 7) – log (mean oocysts on day 14 or 21). Two tool compounds that sustain reduction in parasite shedding for a week beyond treatment are labeled in bold. Independent experimental repeats with additional doses of tool compounds reported in Fig. S4.

To investigate our key parameters, solubility and permeability, we used the Biopharmaceutical Classification System (BCS)(13) to categorize our tool compounds in a methodical way. The BCS (Fig. 1A) is a theoretical classification system which correlates an oral drug’s ability to dissolve in the gastrointestinal tract (solubility) and cross cell membranes in the small intestine (permeability) to enter the systemic circulation.(13) Compounds with high solubility and high permeability (Class I, pink) are predicted to be well-absorbed from the small intestine, whereas compounds with low solubility and low permeability (Class IV, orange) are predicted to have limited absorption. Restricting either of these properties is likely to have a rate-determining effect on absorption (Class II, purple; Class III, teal). Using the BCS to prioritize compounds for *in vivo* studies allowed us to systematically examine these individual property combinations and determine whether modulating solubility or permeability influenced compound efficacy. Our hypothesis was that slow absorption due to suboptimal solubility or permeability (i.e. Class II or Class III) would maximize compound concentration at the biophase following a single oral dose.

Accordingly, we categorized our most potent *Cp*KRS inhibitors (*C. parvum* EC_50_ < 0.2 μM, Fig. S2) according to their measured aqueous solubility and passive permeability data (Table S1). Fourteen exemplars which spanned each of the four BCS classes (Fig. 1B) were prioritized for *in vivo* efficacy studies. DDD706, the early lead, was not included in this comparative study because it was less potent than the optimized leads (*C. parvum* EC_50_ 1.5 μM, Table S1) and was not well tolerated in mice at higher doses. Only two Class IV compounds were suitable for inclusion due to the generally poor *in vitro* parasite activity observed for compounds from this category. It is likely that the limited potency is due to the combination of poor solubility and poor permeability resulting in low intracellular compound concentrations. As *in vivo* efficacy requires compounds to cross both host and parasite cell membranes to exert a cidal effect, good *in vitro* potency was a prerequisite to progress compounds to mouse efficacy studies.

An immunocompromised mouse model of cryptosporidiosis (Fig. 1C) was selected to examine the effect of each of the fourteen tool compounds on parasite reduction *in vivo*. NOD SCID Gamma mice(14) infected with wild type *C. parvum* were treated with test compound orally at 30 or 50 mg/kg twice a day for 7 days. Treatment started on day 7 post infection (PI) and concluded on day 13 PI. Infection was determined by measuring the number of parasites shed in the fecal material by quantitative PCR (oocysts/g). Efficacy was determined by calculating the log reduction in the number of oocysts shed by treated animals after completion of compound dosing (day 14) relative to the initial parasite burden in the same group of animals before treatment began (day 7 PI). Hypothesized compound absorption by BCS class is illustrated in Fig. S3. Results indicate that tool compounds with restricted solubility (Class II, purple) or permeability (Class III, teal) produced the highest reduction of oocyst shedding after treatment (day 14 PI) (Fig. 1D). Every compound from Class II and Class III showed >2.8 log reduction in oocyst shedding, equivalent to >99.8% parasite reduction. Contrastingly, compounds with high solubility and permeability (Class I, pink) or low solubility and permeability (Class IV, orange) did not demonstrate such a pronounced effect on parasite shedding. No Class I or Class IV compound achieved a log reduction above 2.0 (99% parasite reduction). Mice did not exhibit any tolerability issues or adverse events when treated with any of the tool compounds, at any doses.

Due to the exponential nature of *Cryptosporidium* replication, it is possible that if a small number of parasites are not killed by drug treatment, infection can rapidly recrudesce after drug treatment stops. With early lead DDD706 (Fig. S1A), some recrudescence was observed.(8) We sought to determine the impact and risk of recrudescence with our advanced tool compounds. The *Cryptosporidium* life cycle is approximately 72 hours so a one-week period is sufficient to monitor for recrudescence (day 21 PI).(15) For many of the tool compounds the log reduction on day 21 PI was lower than the log reduction at day 14 PI (Fig. S4), indicating incomplete parasite killing and recrudescence once drug pressure was removed. This effect was especially pronounced with compounds that had high solubility and permeability (Class I). One compound each from Class II (DDD489) and Class III (DDD508) displayed superior efficacy and lowest parasite recrudescence in the NOD SCID Gamma model and merited further investigation.

**Figure 2:**
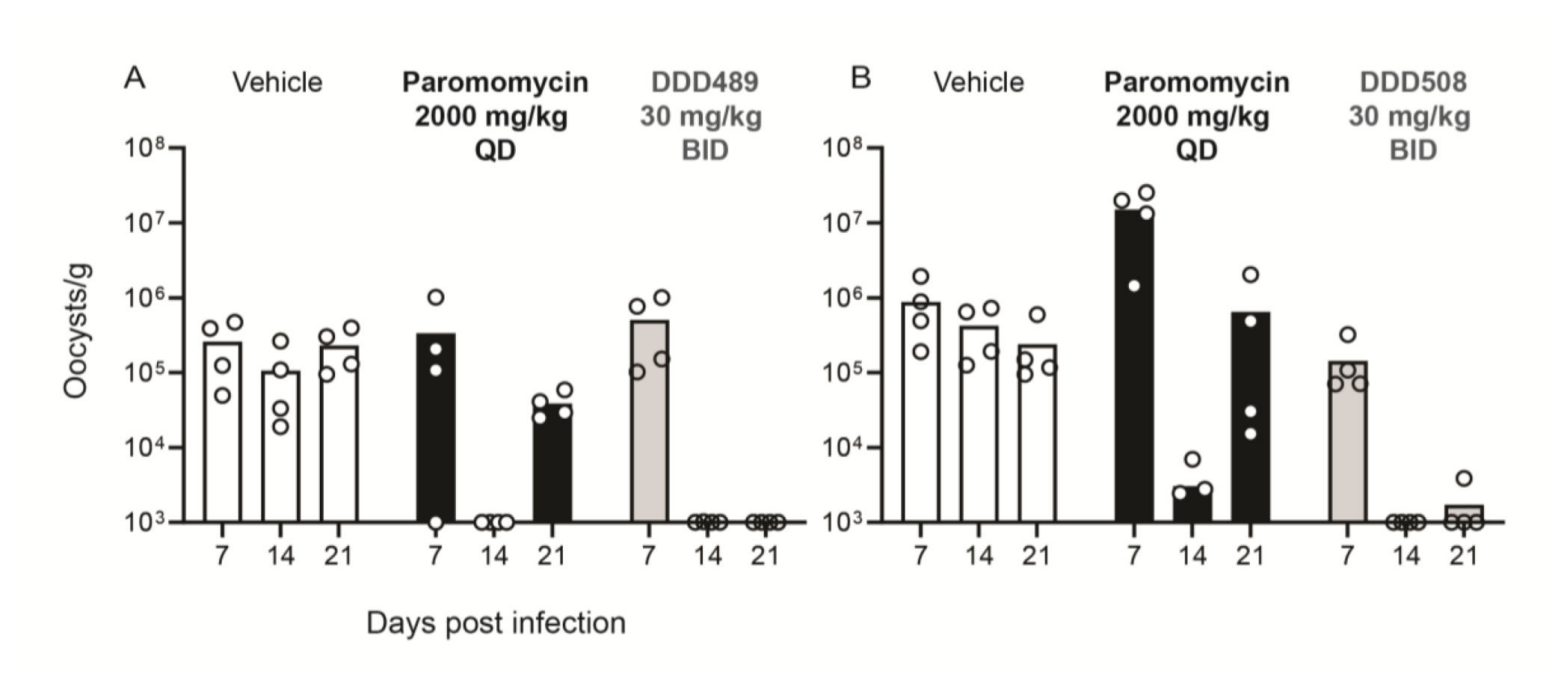
Minimum efficacious dose of DDD489 and DDD508 is 30 mg/kg in NOD SCID Gamma mice. Late lead compounds were administered to NOD SCID Gamma mice as described in Fig. 1C. (**A and B**) Mice were administered vehicle (white), paromomycin at 2000 mg/kg QD (positive control; black), and DDD489 (**A**, gray) or DDD508 (**B**, gray) at 30 mg/kg BID. Representative experiment shown. Independent experimental repeats with additional doses reported in Fig. S5. Bar height indicates average for each treatment group (n=4 mice per cage for all compounds tested), points indicate individual animals (average of three technical replicates plotted). Y-axes begin at limit of quantitation (LOQ).

Follow-up studies were conducted to determine the lowest efficacious dose of DDD489 and DDD508. When administered orally at 10 mg/kg twice a day for 7 days, both compounds resulted in >2.8 log reduction in parasite shedding (>99.8% parasite reduction) but were unable to prevent recrudescence at this dose. A dose-ranging experiment, conducted to determine the dose capable of reducing parasite burden and preventing recrudescence identified 30 mg/kg as the minimum efficacious dose (MED) for both DDD489 and DDD508 (Fig. 2, additional doses reported in Fig. S5).

### Systemic drug concentration is unlikely to be a major driver of efficacy for DDD489 and DDD508

**Figure 3:**
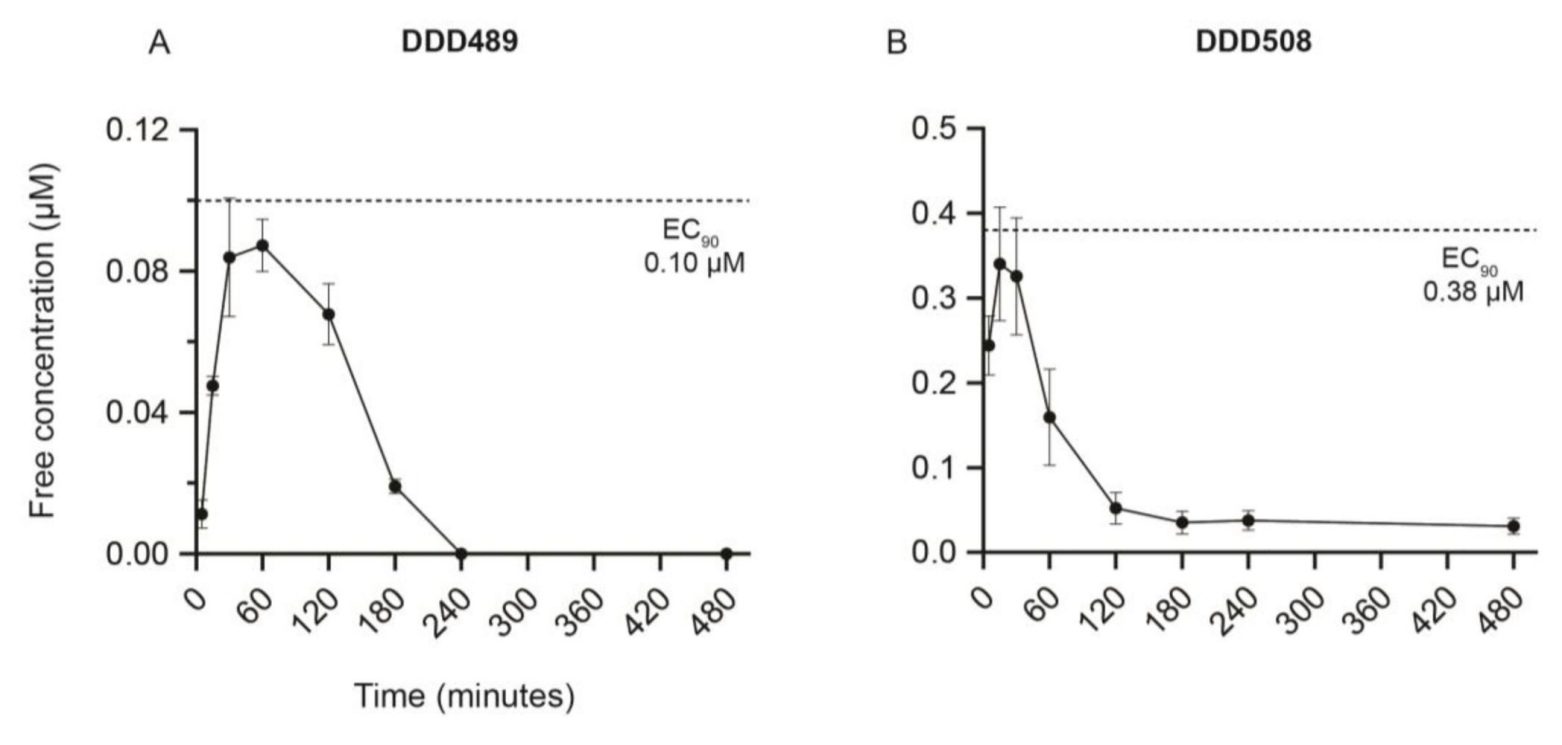
Low solubility or permeability results in low oral bioavailability in mice for late lead compounds. Mean free blood concentration time profiles after a single oral dose (10 mg/kg) of DDD489 **(A)** or DDD508 (**B**) to female BALB/c mice (n=3 mice per group for all compounds tested). Data are mean ± SD. Total drug concentrations were corrected for fraction unbound (fu) (DDD489 fu: 0.16; DDD508 fu: 0.6) assuming blood-to-plasma ratio of 1. Dotted lines indicate *C. parvum* EC_90_, calculated from average EC_50_ and hill slope (DDD489: EC_50_ 0.043 μM, hill slope 2.7; DDD508: EC_50_ 0.13 μM, hill slope 2.1). Both compounds have low oral bioavailability and low clearance (DDD489: F=7%, Clb=17% LBF; DDD508: F=6%, Clb=27% LBF).

Pharmacokinetic studies were carried out for these two efficacious compounds (*in vitro* DMPK reported in Table S2). Because both late lead compounds demonstrated efficacy when administered at oral doses as low as 10 mg/kg (Fig. S5), this dose was used for pharmacokinetic evaluation to understand the relationship between PK and efficacy (PKPD). Uninfected mice were administered a single oral (10 mg/kg) dose of either DDD489 or DDD508 using the same formulation and dose concentration as for the efficacy study. Blood samples were collected at various time points after drug administration (15, 30, 60, 120, 180, 240, and 480 minutes) and analyzed to produce a concentration-time plot of unbound drug concentration (Fig. 3). PK parameters from intravenous (IV) and oral (PO) dosing and cross-species PK data are shown in Table S3.

From the BCS, it was likely that both compounds would have low oral bioavailability due to suboptimal absorption because of restricted solubility (DDD489, Class II) or permeability (DDD508, Class III). This was reflected in the experimental results, with DDD489 and DDD508 having oral bioavailability (F) of 7% and 6% respectively, despite only having 17% and 27% liver blood flow (LBF) clearance, respectively. When the compounds were dosed orally at 10 mg/kg in uninfected mice, the mean free (unbound) drug concentrations of both compounds remained below the EC_90_ for the duration of the study (Fig. 3). In NOD SCID Gamma mice, at the MED (30 mg/kg), free blood concentrations of DDD489 and DDD508 were only above the EC_90_ for around two hours when measured on the first and last days of compound treatment (Fig. S6). Therefore, the substantial reduction of parasite shedding *in vivo*, despite the low oral bioavailability, suggested that systemic concentration was unlikely to be a major driver of efficacy for these compounds.

DDD489 and DDD508 (Table 1) displayed potent inhibition of the *Cp*KRS enzyme and excellent *in vitro* activity against both *C. parvum* and *C. hominis*. They were also selective over *Hs*KRS and showed low cytotoxicity in *Hs*HepG2 cells. Cellular selectivity (*Hs*HepG2 EC_50_/*C. parvum* EC_50_) was high for both compounds (450-fold and 140-fold for DDD489 and DDD508, respectively). As these compounds represented two different chemical series with similar *in vitro* profiles (aside from their solubility and permeability) and showed comparable efficacy and prevention of recrudescence in NOD SCID Gamma mice, we decided to profile them using additional animal models of cryptosporidiosis infection.

### DDD489 and DDD508 are efficacious in additional cryptosporidiosis mouse models

Several mouse efficacy models for cryptosporidiosis are available, but it is unclear which will be most predictive of clinical outcome. Therefore, we evaluated the efficacy of DDD489 and DDD508 in several *in vivo* models. This included the acute efficacy model (interferon-gamma knockout mice, IFN-Gamma KO) which may be more representative of infection in young children, and a chronic efficacy model developed herein which may be more representative of chronically infected immunocompromised adults. The ability to translate results from these animal models to clinical outcomes could help refine the drug discovery cascade for cryptosporidiosis.

**Figure 4:**
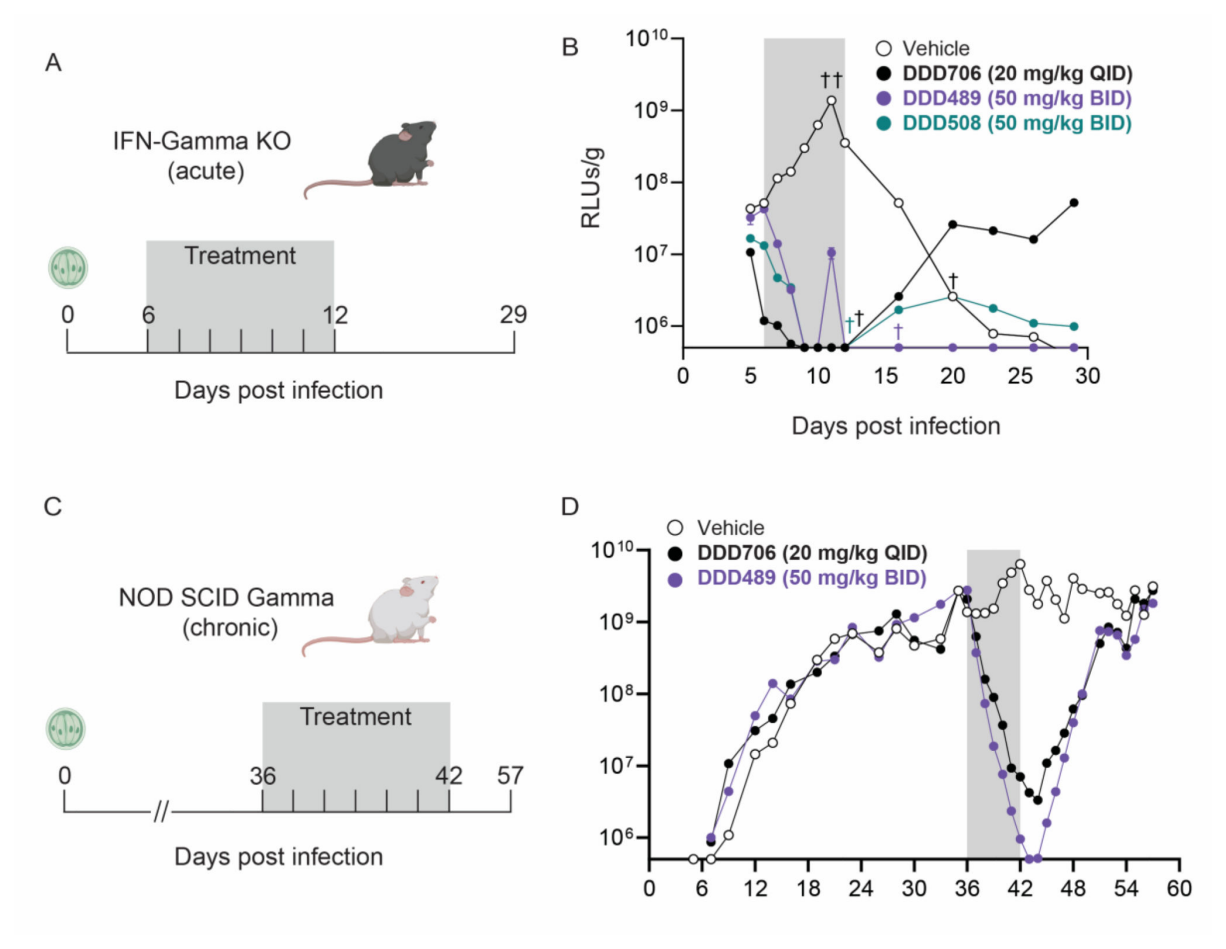
Late leads DDD489 and DDD508 are effective in key cryptosporidiosis mouse models. Efficacy of late lead compounds was assessed in an Interferon-Gamma KO model (acute model of cryptosporidiosis) and in chronically infected NOD SCID Gamma mice. Fecal samples were collected and pooled from each cage of mice; parasite shedding was quantified via NanoLuciferase assay (relative luminesce units, RLUs/g; average of three technical replicates plotted). Y-axis begins at LOQ. (**A**) Interferon-Gamma KO mice were infected with transgenic *C. parvum* that express NanoLuciferase. Treatments were administered by oral gavage starting on day 6 PI for 7 days (gray box indicates treatment period; n=5 mice per cage for all compounds tested). (**B**) Mice treated with vehicle (white), DDD706 (positive control, black), or DDD508 (teal). Associated PK data and infection quantification of individual mice reported in Fig. S6 and S7, respectively. ^†^Mice were culled due to weight loss. **(C)** NOD SCID Gamma mice were infected with transgenic *C. parvum* that express NanoLuciferase. Fecal samples collected, pooled, analyzed, and graphed as in **B** (n=4 mice per cage for all compounds tested). Mice were treated when infection was chronic and high (RLUs/g >10^9^ for over a week). Treatment was administered via oral gavage starting on day 36 PI for 7 days (gray box indicates treatment period). (**D**) Mice treated with vehicle (white), control DDD706 (black), or DDD489 at 50 mg/kg BID (purple).

First, compounds were tested in the acute cryptosporidiosis mouse model, IFN-Gamma KO.(16) In this model, infection peaks at approximately 10-12 days PI, and resolves within a month. Mice were infected with a strain of transgenic *C. parvum* that expresses NanoLuciferase (NLuc).(12) Mice were treated orally for 7 days starting on day 6 PI (Fig. 4A) and a NanoLuciferase assay was used to determine parasite shedding in feces (RLUs/g). Control mice received either vehicle (Fig. 4B, white) or positive control, DDD706 (Fig. 4B, black; NLuc parasites are genetically engineered to be resistant to paromomycin, so DDD706 serves as the positive control) at 20 mg/kg QD; DDD489 and DDD508 were tested at 50 mg/kg BID.

Compounds DDD508 (Fig. 4B, teal) and DDD706 (Fig. 4B, black) reduced parasite shedding below the limit of quantitation (LOQ) during the treatment period but were unable to prevent recrudescence. However, DDD489 (Fig. 4B, purple) reduced parasite shedding below the LOQ and was able to prevent parasite recrudescence. All individual animals were negative for *Cryptosporidium* infection after treatment with DDD489 (Fig. S7). These results suggest that DDD489 demonstrated superior efficacy and aided in the prioritization of this compound for testing in an additional mouse model.

We next developed a mouse efficacy model that mimics chronic infection. In this model, NOD SCID Gamma mice were allowed to become highly infected (>10^9^ RLU) and maintain this for a minimum of one week before compound treatment was administered (Fig. 4C). To achieve efficacy against this chronic infection, compounds must kill more parasites, offering a more rigorous method for assessing suitability for progression.

DDD489 was able to reduce parasite shedding below the LOQ by the end of the treatment period (Fig. 4D). However, with this dosing regimen, DDD489 failed to prevent recrudescence. Further work is needed to determine the dose of DDD489 that is required to cure chronic infection and prevent recrudescence in this model. However, we believe that this chronic mouse model could have wider applications in the development of future anti-cryptosporidial compounds as it offers a more rigorous evaluation of efficacy, requiring a higher amount of parasite killing and facilitating a clearer interpretation of recrudescence risk.

### DDD489 and DDD508 are highly efficacious in a disease-relevant calf model

With evidence that both late lead compounds were highly effective at reducing infection in mouse models, we proceeded to investigate their efficacy in a natural infection model. Unlike mice which must be immunocompromised to allow *C. parvum* infection and do not exhibit clinical symptoms, neonatal calves are natural hosts and develop diarrhea and dehydration. By testing compounds in a neonatal calf model of cryptosporidiosis it is possible to assess reduction in parasite shedding as well as resolution of symptoms including diarrhea. Additional clinical observations such as changes in demeanor and appetite can also be scored.

**Figure 5:**
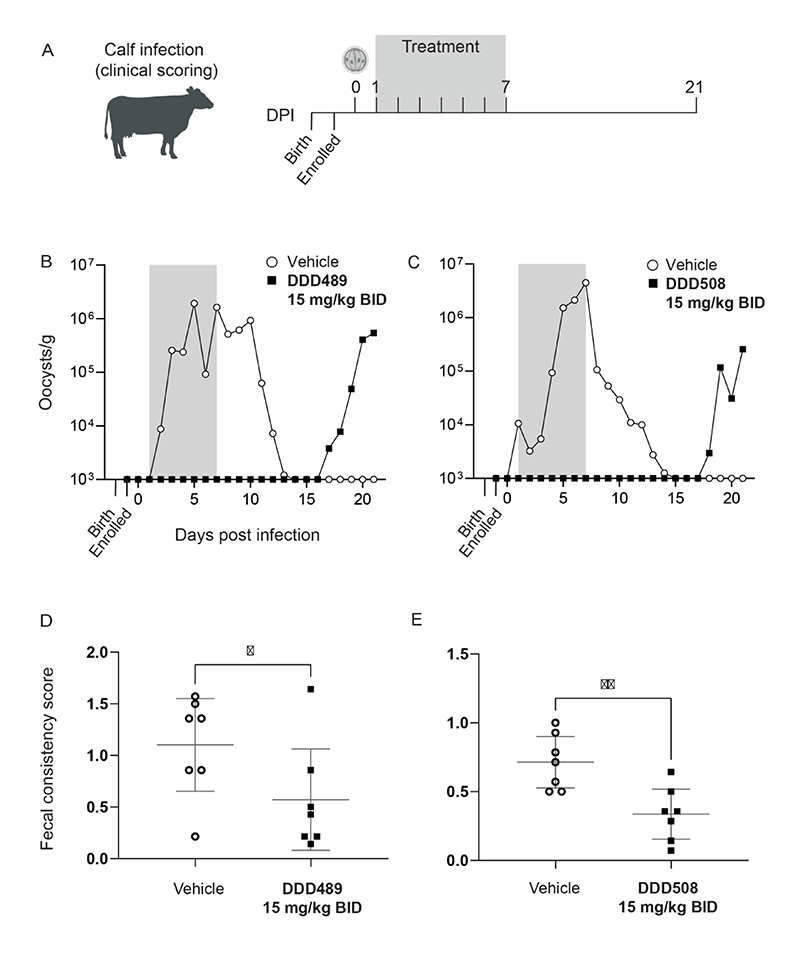
DDD489 and DDD508 reduce parasite shedding and diarrheal scores in calves. **(A)** Neonatal calves are a large animal model and natural host model of cryptosporidiosis. Calves were enrolled in the study at the time of birth and randomly assigned to treatment or control groups (n=7 calves per group). Calves were challenged with wild type oocysts at two days old (0 days PI). Treatment with vehicle or late lead compound was administered orally at 15 mg/kg BID, starting at day 1 PI for 7 days (gray box indicates treatment period). Fecal samples were collected from individual calves daily starting the day prior to infection and ending day 21 PI. (**B and C**) Calves were treated with vehicle (white), DDD489 (**B**, black) or DDD508 (**C**, black). Associated PK data shown in Fig. S10. Parasite shedding was quantified by qPCR and by microscopy; mean values from each group plotted (see Fig. S8 and S9 for microscopy and for individual animal data for qPCR and microscopy). Y-axes begin at LOQ. (**D and E**) Mean fecal consistency scores recorded during treatment period (day 1 to day 7 PI) for vehicle and treated animals. Diarrhea was scored based on fecal consistency (for scoring guide see Table S4). Points represent individual animals, with mean and 95% confidence intervals plotted as horizontal gray lines. P value was determined using the unpaired, single-sided student’s t test (**D**) p=0.0375 (*) (**E**) p=0.002 (**)

Calves were enrolled at birth and challenged with wild type *C. parvum* oocysts (Fig. 5A). DDD489 and DDD508 were administered orally at 15 mg/kg BID for 7 days, beginning on day 1 PI. Blood was sampled and mean free plasma concentration was determined (Fig. S10). Fecal samples were collected from individual animals and parasite shedding was quantified (oocyst/g) by microscopy and quantitative PCR. Fecal samples were collected daily until day 21 PI to monitor for parasite recrudescence. To determine the impact of our late lead compounds on reducing symptoms of cryptosporidiosis, several clinical observations were scored throughout the study.

The vehicle control group in both studies had high parasite shedding, with infection peaking around day 5-7 PI before self-resolving. Conversely, calves that were treated with either DDD489 (Fig. 5B) or DDD508 (Fig. 5C) did not shed any detectable amount of *Cryptosporidium* during treatment or up to 7 days afterwards (to day 16 PI), demonstrating excellent efficacy. Fecal consistency scores were recorded throughout the study to assess the severity of diarrhea observed for each of the calves (0 = normal, 3 = severe, Table S4). Calves treated with either DDD489 or DDD508 all had fecal scores in the normal to mild range (Fig. 5D and E). This represented a significant (DDD489: p=0.0375, DDD508: p=0.002) improvement in fecal consistency compared to vehicle. However, vehicle-treated individuals failed to demonstrate severe diarrhea throughout the study and overall fecal consistency scores were low (≤ 2). Increasing the *C. parvum* oocyst challenge may have increased the severity of diarrhea and allowed for a larger scoring range. However, this was not considered due to ethical implications for animal welfare.

Unlike previously published calf efficacy studies,(17–19) we monitored parasite shedding for two weeks after treatment finished. Once infection had self-resolved in the vehicle group, no parasites were detected for the remainder of the study (data from individual calves is shown in Fig. S8 and S9). However, approximately half of the calves receiving treatment (3/7 for DDD489 and 4/7 for DDD508) shed *Cryptosporidium* after day 16 PI.

Resistance generation towards test compound has previously been observed for another tRNA-synthetase inhibitor in the calf model.(19) To understand if the late and sporadic shedding we observed could be a result of resistance, DNA sequencing of *CpKRS* was performed on fecal samples recovered from each of the three *Cryptosporidium* positive calves from the study with DDD489. No mutations of the *Cp*KRS gene were observed in any of the oocysts shed from any of the three calves (Fig. S11), confirming that this effect was not due to resistance generation in the target, *Cp*KRS.

### Over-expression or mutation of *Cp*KRS confers resistance to DDD489 and DDD508

To deliver effective late leads, considerable compound optimization had occurred to increase potency and selectivity and refine physicochemical properties. Although DDD508 originated from the same chromone-containing series as our previously published *Cp*KRS inhibitor DDD706,(8) DDD489 is structurally distinct. Therefore, it was important to confirm that both compounds were acting on-target for *Cp*KRS. We previously reported an *in vitro Cp*KRS drug sensitivity assay(12) using a *Cryptosporidium* strain that had been genetically modified to overexpress the molecular target (KRS-OE). *Cp*KRS overexpression in this modified strain was confirmed by quantitative proteomics analysis (Fig. S12). This strain should be less susceptible to drug treatment if the compounds are acting on-target. DDD489 and DDD508 showed a loss in potency against the modified strain compared to wild type *Cp*KRS (Fig. S13), providing evidence that *Cp*KRS was indeed the molecular target for both compounds.

**Figure 6:**
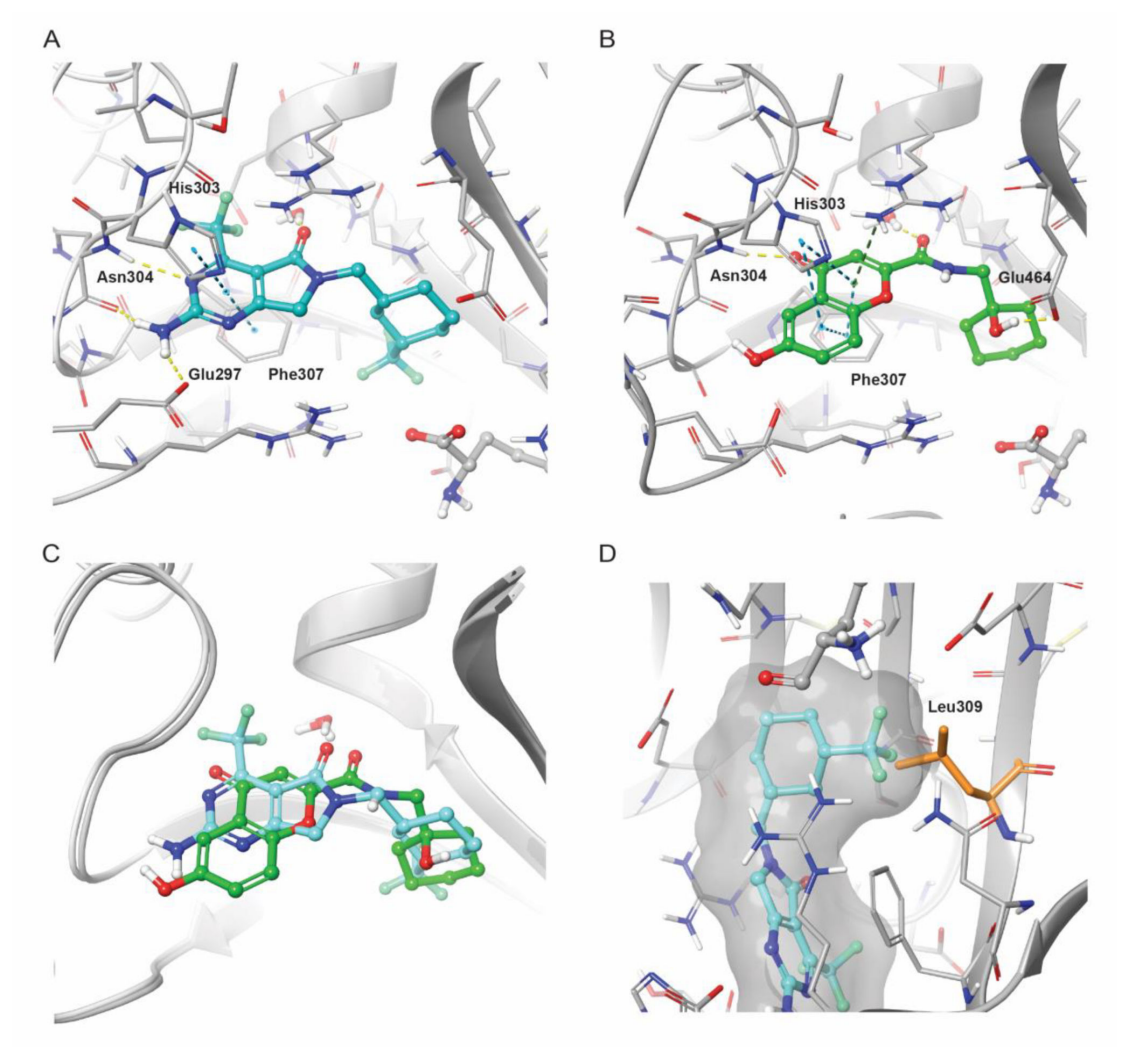
DDD489 and DDD508 bind in a similar pose in the ATP site of *Cp*KRS. (**A and B**) Crystal structures of DDD489 (**A**, blue, PDB ID 8R2A) and DDD508 (**B**, green, PDB ID 8S00) bound to the ATP site of *Cp*KRS. Hydrogen atoms shown in calculated positions. Dotted lines represent hydrogen bonding (yellow), pi stacking (blue), and pi-cation (green) interactions. Key residues labeled for clarity. (**C**) Overlay of DDD489 (blue) and DDD508 in the ATP site of *Cp*KRS. (**D**) Docked pose of DDD489 (blue) in the mutated KRS-A309L strain showing the anticipated clash between the mutated Leu309 residue (orange) and the trifluoromethyl-substituted cyclohexyl ring when bound in the hydrophobic ribose pocket.

A structure-based drug design approach was used to optimize these inhibitors so knowledge of how the compounds bound to the enzyme active site could be exploited to provide additional evidence of on-target activity. We obtained crystal structures of DDD489 (PDB ID: 8R2A) and DDD508 (PDB ID: 8S00) which determined that the compounds bound in the ATP site, making comparable interactions despite the differences between the two heterocycles (Fig. 6). In both series, the cyclohexyl ring pointed into the hydrophobic ribose pocket. Mutation of a single amino acid residue within this pocket in *Pf*KRS conferred resistance towards inhibitors(20) and so a similar effect was expected for *Cp*KRS. It was theorized that mutating an alanine to a leucine residue within this pocket would result in a steric clash with the cyclohexyl ring and confer resistance towards these compounds.(12) A *Cryptosporidium* strain with an A309L mutation in this region of the enzyme active site was shown to confer resistance to the early lead, DDD706.(12) This genetically modified strain (KRS-A309L) was also less susceptible to treatment with DDD489 and DDD508 (Fig. S13), indicating drug resistance in the presence of this mutation, and supporting our presumption that these compounds were killing parasites through inhibition of *Cp*KRS.

## Discussion

Cryptosporidiosis is a leading cause of death in malnourished children, particularly in LMICs, yet there is currently no vaccine or effective treatment. Safe therapeutics are urgently needed to treat this neglected disease in susceptible patient populations. To support drug discovery, a target product profile(21) and clinical development pathway(22) have been proposed and several advanced compounds are now in preclinical development. Although this represents substantial progress towards a new cryptosporidiosis treatment, few candidates have reached clinical trials to date, and it is not known how efficacy will translate from preclinical models to humans. The discovery of novel anti-cryptosporidials and advancement of current leads is still required to ensure a robust pipeline and offset anticipated attrition.(7) Furthermore, increasing our understanding of the key properties required for achieving efficacy will assist in future design of optimized compounds capable of killing this complex gastrointestinal parasite.

The location of parasites presents a unique challenge for cryptosporidiosis drug discovery. *Cryptosporidium* infect and replicate in a parasitophorous vacuole inside epithelial cells lining the small intestine.(9) Drug molecules must cross both the intestinal cell membrane and the parasite membrane. It is likely that specific tailoring of chemical properties will be required to ensure compounds reach the parasites at sufficient concentrations to maximize efficacy *in vivo*. However, due to the small number of advanced compounds in development, there is a limited understanding of the optimal property profile for treating this gastrointestinal infection.

Having developed two series of potent, selective lysyl-tRNA synthetase inhibitors, this was an ideal opportunity to use these as tool compounds to address this knowledge gap. A key unanswered question was whether systemic drug concentration of anti-cryptosporidial compounds is required for efficacy. Previous reports demonstrate contrasting outcomes; some show an apparent improvement in efficacy with increasing oral bioavailability(23) and others show efficacy in the absence of high systemic exposure.(16, 17) Physiologically-based PK modeling predicted that gastrointestinal exposure, not systemic exposure, of a series of *C. parvum* bumped kinase inhibitors correlated better with observed *in vivo* efficacy.(24) If systemic exposure of *Cp*KRS inhibitors is not essential for efficacy, slowing down compound absorption across the GI tract following a single oral dose could maximize drug concentration at the desired site of action and consequently improve efficacy. Additionally, if systemic exposure is not a driver of efficacy, a targeted approach to lower systemic drug concentration could potentially mitigate the risk of toxicity and off-target effects. As patients are mainly children under the age of five years(21) living in low- and middle-income countries with limited access to medical treatment, an excellent safety profile is critical.

We adopted a methodical approach to investigating optimal physiochemical properties of our lead series. As solubility and permeability are two important factors affecting oral drug absorption, we used these properties as criteria for selecting tool compounds. The BCS was used to categorize compounds into four classes which relate to their likelihood of achieving good oral absorption. By using this systematic approach, we were able to select a range of potent compounds that would allow us to examine the effects of restricting absorption completely (Class IV), partially (Classes II and III) or not at all (Class I) on parasite reduction *in vivo*.

Tool compounds were tested in a NOD SCID Gamma immunocompromised mouse model. Soluble and permeable Class I tool compounds, predicted to have good oral bioavailability according to the BCS, reduced parasite shedding by the end of the treatment period. However, these compounds failed to kill all the parasites and we observed recrudescence a week after treatment ended. We hypothesize that these compounds were absorbed too rapidly from the small intestine, so sufficient concentrations were not maintained in the gut wall for long enough, resulting in incomplete parasite killing.

Conversely, Class IV compounds are likely retained in the GI tract due to their limited ability to dissolve and penetrate cell membranes to be absorbed and enter the systemic circulation. We observed diminished parasite-killing activity with Class IV compounds and consequently, a limitation of our study was that only two compounds were included from this category. We hypothesize that the combination of low solubility and permeability prevents the compounds from permeating the gut wall and entering into the *Cryptosporidium* vacuole itself. Because the parasites are intracellular, compounds must be capable of crossing both intestinal cell and parasite membranes to exert an effect. Some degree of solubility and permeability is therefore likely to be required to achieve this.

If our hypotheses for Class I and Class IV compounds hold true, formulation development could potentially be used to enhance the efficacy of compounds in these BCS classes. Class I compounds could be maintained at higher concentrations in the gut through slow-release formulation, whereas Class IV compounds could be formulated to enhance absorption through the gut wall and into the *Cryptosporidium* vacuole.

Compounds with rate-limiting absorption due to solubility (Class II) or permeability (Class III) showed an improvement in efficacy in the NOD SCID Gamma model compared to the other classes of tool compounds, as we predicted. Compounds with this balance of properties are generally slower to be absorbed and, after a single oral dose, should have extended exposure at the target site. Two compounds demonstrated superior efficacy (>2.8 log unit reduction in oocysts) in this model and were able to prevent recrudescence. PK studies in uninfected mice showed that these compounds exhibited very low concentrations in blood (mean unbound blood concentration of both compounds did not exceed the EC_90_ when dosed orally at 10 mg/kg) which suggests systemic exposure was unlikely to drive efficacy of these compounds.

It was not possible to completely restrict systemic exposure of these compounds, as the balance of solubility and permeability that was important for maintaining prolonged efficacy still allowed compounds to be absorbed, albeit slowly. A soft-drug approach, exploiting high first pass metabolism by the liver would be an attractive way to retain efficacy but minimize systemic exposure.(25) This said, systemic concentrations of both late lead compounds were very low compared to our early lead in mice (DDD706, oral bioavailability = 100%) so the risk of toxicity due to off-target effects is likely to be lower, depending on exposure in humans. Subsequent *in vitro* investigation of potential toxicity or adverse effects did not highlight any concerns.

Optimized late leads were progressed for further investigation and showed excellent efficacy in an IFN-Gamma KO mouse model of acute infection. Both compounds reduced parasite shedding below the LOQ during treatment and DDD489 was also able to prevent recrudescence in this model. Subsequently, we developed a cryptosporidiosis mouse model to better evaluate drug efficacy in chronic infections. NOD SCID Gamma mice were allowed to develop high levels of infection that had become stabilized before treatment began. These mice do not resolve the infection, allowing recrudescence to be clearly observed. In this model, DDD489 showed a reduction in parasite shedding to the LOQ, however recrudescence was observed. A higher or longer dose may be required to prevent recrudescence in this chronic model. This could be determined in future studies.

Both compounds were tested in a calf efficacy model. Treatment with DDD489 and DDD508 represented a sustained reduction in oocyst shedding in neonatal calves. No detectable amount of *Cryptosporidium* was measured in feces from any of the treated calves for the duration of treatment. Both late leads also showed significantly (DDD489: p=0.0375, DDD508: p=0.002) lower fecal consistency scores compared to vehicle.

Ultimately, evaluation of compounds in various animal models allowed us to rigorously evaluate our series of *Cp*KRS inhibitors and progress them through the drug discovery cascade. Initial triage of tool compounds in mice furthered our understanding of optimal property combinations, demonstrating that a balance of suboptimal solubility or permeability was necessary to slow down GI absorption to achieve better parasite reduction and prevent recrudescence. Additional mouse models allowed us to differentiate between the two late lead compounds as DDD489 was superior at preventing recrudescence. Last, the calf model provided evidence that the compounds were efficacious in a natural host of infection, supporting the progression of both late lead compounds to preclinical candidate selection phase, pending further *in vivo* safety studies.

One limitation of our study is that we explored the role of solubility and permeability in relation to a single molecular target. To expand upon these results, it would be interesting to explore the influence of these parameters across a wider scope of targets. This could include targets with different modes of action, expression levels, or localization within the parasite.

The results from this study allow us to predict, based on *in vitro* properties, whether analogues within our *Cp*KRS inhibitor series are likely to show efficacy *in vivo*. It is hoped that these learnings could also be applied to the design of anti-cryptosporidials and refine the drug discovery pipeline for cryptosporidiosis. Predicting the likelihood of achieving good efficacy earlier in the screening cascade and establishing which animal models are most predictive of clinical outcome, could help prevent attrition in the development pathway.

## Materials and Methods

### Study design

The objective of this study was to evaluate *Cp*KRS inhibitors in animal models of cryptosporidiosis to establish the relationship between *in vitro* properties and *in vivo* efficacy. Fourteen potent tool compounds were selected according to their measured solubility and permeability to systematically examine each BCS class. NOD SCID Gamma mice, infected with wild type *C. parvum* oocysts, were treated with compounds as outlined in Fig 1C. Efficacy was determined by measuring oocyst shedding in feces by qPCR on the days indicated. Lead compounds were subsequently profiled in additional mouse models of infection as detailed in Fig 4A and 4C. IFN Gamma KO or NOD SCID Gamma mice were infected with NanoLuciferase-expressing transgenic parasites and oocyst shedding in feces was measured by NanoLuciferase assay. Mice were randomly housed together at time of weaning and assigned to treatment or control groups at time of infection. Mice were aged and sexed matched within each experimental cohort, mice with any observed health issues or weight outside the average for the group were not enrolled in the study. Group size of a 3-6 mice per cage, as indicated in corresponding figure legends. Where group size was reduced over the course of the experiment due to the need to cull animals due to welfare considerations, details have been included. Mice were provided food and water ad libitum and housed on a light-dark cycle of 12 hours.

To assess efficacy in a natural host model of *Cryptosporidium* infection, lead compounds were administered to newborn calves as outlined in Fig 5A. Calves were enrolled in the study at the time of birth, randomly assigned to treatment or control groups, and infected with wild type *C. parvum*. Full details of blinding and inclusion/exclusion criteria are provided in the supporting information. Ethical review determined seven calves per group as the minimal group size required for appropriate statistical analysis. Fecal samples were collected daily, and oocyst shedding was quantified by microscopy and qPCR. Clinical observations (Table S4) were scored throughout the study to determine the impact on reducing symptoms of cryptosporidiosis.

For each study, the number of animals per group and individual replicates are indicated. Animal care was in accordance with institutional guidelines, see supplemental information for details.

### NOD SCID Gamma mouse efficacy with tool compounds (Vermont)

Efficacy of tool compounds in the NOD SCID Gamma mouse model was assessed as previously described.(14) Male mice aged 3-5 weeks old were used, 4 mice per group (NOD.Cg-*Prkdc^scid^ Il2rgtm1Wjl*/SzJ; purchased from Jackson Labs). Mice were infected via gavage (100,000 wild type Iowa strain *C. parvum* oocysts from Bunchgrass Farms). Compounds were prepared fresh daily in HPMC with DMSO (0.5% hydroxypropylmethylcellulose, 0.4% Tween 80 and 0.5% Benzyl alcohol (v/v) plus 5% DMSO) and were administered by oral gavage at the concentration indicated starting 7 days post infection. Paromomycin served as the positive control (administered at 2000 mg/kg). Fecal samples were collected on days indicated. Oocyst shedding in feces was measured using a previously validated qPCR assay.(28)

### IFN-Gamma KO mouse efficacy with late leads (Dundee)

Male IFN-Gamma knockout mice (B6.129S7-Ifng^tmlTS^/J, JAX 002287; purchased from Jackson Labs and bred at the University of Dundee) aged 30 weeks old were used, (*n* = 5 mice per experimental group). Mice were infected by oral gavage (100 oocysts of Δ*tk*::mNeon-Neo^R^ as described previously(12)). Compounds were prepared in HMPC without DMSO and administered by oral gavage at 50 mg/kg starting day 6 PI. Early lead compound DDD706 served as the positive control (administered at 20 mg/kg QD). Fecal samples were pooled from all mice in the cage on days indicated. Oocyst shedding in feces was determined by NanoLuciferase assay (20 mg of fecal material was homogenized in 1 ml of lysis buffer and assayed for NLuc activity as previously described).(12)

### NOD SCID Gamma chronic mouse efficacy with DDD489 (Dundee)

Male NOD SCID Gamma mice (NOD.Cg-*Prkdc^scid^ Il2rgtm1Wjl*/SzJ; purchased from Charles Rivers) aged 10 weeks old were used (*n* = 4 mice per experimental group). Mice were infected by oral gavage (5,000 oocysts of Δ*tk*::mNeon-Neo^R^). Oocyst shedding in feces was determined by NanoLuciferase assay as described above. Once infection became chronic (at least 1 week of fecal RLUs/g at >10^9^), compounds were administered. DDD489 was prepared in HPMC without DMSO and administered by oral gavage at 50 mg/kg starting day 36 post infection. Early lead compound DDD706 served as the positive control (administered at 20 mg/kg QD). Fecal samples were pooled from all mice in the cage on days indicated.

### Calf efficacy with late leads (Moredun Scientific)

Calves (Holstein Friesian, British Blue Cross, Limousin Cross, or Aberdeen Angus Cross) were born at commercial dairy farms, provided a colostrum feed, and transported to Moredun Scientific. Calves were enrolled in the study if >30 kg at birth and in good health. Enrolment was staggered due to timing of births.

Calves were assigned to control or treatment groups (7 calves per group) and housed in separate barns (within each barn calves were housed in adjacent, individual pens). Male calves were used in the DDD489 study, and both male and female calves were used in the DDD508 study (control group: 3 females/4 males; treatment group: 2 females/5 males). Calves were challenged on day 1 post birth with 5x10^5^ *C. parvum* oocysts by oral gavage (Sterling Labs for DDD489; Waterborne Inc. for DDD508).

Starting day 1 PI, vehicle (in 0.5% HPMC, 0.4% Tween 80, 0.5% Benzyl alcohol in sterile water) or treatment (DDD489 or DDD508) were administered by oral gavage twice daily immediately prior to milk feeds, and at least 8 hours apart (± 30 minutes). Vehicle was delivered at a set volume (22.6 ml per occasion) and test compound at 15 mg/kg (maximum volume of 20 ml per occasion; prepared in vehicle). Blood samples were collected from all animals prior to morning treatment on Day 1, then 5 (± 2) minutes, 15 (± 2) minutes, 30 (± 5) minutes, 1, 2, 4, 8 and 24 hours (± 10 minutes) post first daily dose. This sample schedule was repeated on Day 7 for the same animals. Blood samples were processed to plasma. Weight was measured and clinical observations recorded as indicated. Fecal samples were collected daily throughout the study.

### Statistical analysis

P values were determined using the unpaired, single-sided student’s t test, assuming Gaussian distribution. Statistical significance was defined as *p* < 0.05. EC_50_ curves for *in vitro* assays were plotted using four parameter logistic non-linear regression. Analysis was performed using GraphPad Prism (version 10.1.2).

## Supplementary Material

Compound Spectra

Supplemental information

## Figures and Tables

**Table 1:** Late leads DDD489 and DDD508 are potent, selective *Cp*KRS inhibitors.

	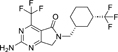	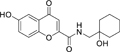
	DDD489	DDD508
***Cp*KRS IC_50_ (μM)** ^a^	0.85 (0.71 – 1.00)	1.70 (1.26 – 2.34)
***C. parvum* EC_50_ (μM)** ^b^	0.04 (0.04 – 0.05)	0.13 (0.08 – 0.20)
***C. hominis* EC_50_ (μM)** ^c^	0.04 (0.03 – 0.07)	0.1 (0.02 – 0.26)
***Hs*KRS IC_50_ (μM)** ^d^	22 (16 – 29)	10 (7.1 – 13)
**HepG2 EC_50_ (μM)** ^e^	18 (10 – 35)	18 (9.5 – 33)
**Cellular selectivity (fold)** ^f^	450	140
**Aqueous solubility (μM)** ^g^	21	184
**PAMPA Papp (nm/s)** ^h^	171	7

Potency data are mean (95% confidence interval). Biochemical and cellular potency data are means of at least 4 and 2 biological replicates, respectively. Solubility and permeability data are means of 2 technical replicates.

a *C. parvum* lysyl-tRNA synthetase enzyme assay.

b Inhibition of *C. parvum* growth when co-cultured with HCT-8 cells for 48 hrs.

c Inhibition of *C. hominis* when co-cultured with HCT-8 cells for 48 hrs.

d *Homo sapiens* lysyl-tRNA synthetase enzyme assay.

e HepG2 is a human liver cancer cell line.

f Cellular selectivity is the ratio of mean HepG2/*C. parvum* EC_50_.

g Aqueous solubility is kinetic solubility measured using UHPLC.

h PAMPA is Parallel Artificial Membrane Permeability Assay; Papp is apparent permeability.

## Data Availability

All data are available in the main text or the supplementary materials.

## One Sentence Summary

Potent lysyl-tRNA synthetase inhibitors advance our understanding of desirable properties for achieving efficacy in cryptosporidiosis animal models.

## References

[1] GBD 2017 Causes of Death Collaborators (2018). Global, regional, and national age-sex-specific mortality for 282 causes of death in 195 countries and territories, 1980–2017: a systematic analysis for the Global Burden of Disease Study 2017. Lancet.

[2] Kotloff KL, Nataro JP, Blackwelder WC, Nasrin D, Farag TH, Panchalingam S, Wu Y, Sow SO, Sur D, Breiman RF, Faruque ASG (2013). Burden and aetiology of diarrhoeal disease in infants and young children in developing countries (the Global Enteric Multicenter Study, GEMS): A prospective, case-control study. Lancet.

[3] Kotloff KL, Nasrin D, Blackwelder WC, Wu Y, Farag T, Panchalingham S, Sow SO, Sur D, Zaidi KM, Faruque ASG, Saha D (2019). The incidence, aetiology, and adverse clinical consequences of less severe diarrhoeal episodes among infants and children residing in low-income and middle-income countries: a 12-month case-control study as a follow-on to the Global Enteric Multicenter Study (GEMS). Lancet Glob Health.

[4] Abubakar I, Aliyu SH, Arumugam C, Usman NK, Hunter PR (2007). Treatment of cryptosporidiosis in immunocompromised individuals: Systematic review and meta-analysis. Br J Clin Pharmacol.

[5] Amadi B, Mwiya M, Musuku J, Watuka A, Sianongo S, Ayoub A, Kelly P (2002). Effect of nitazoxanide on morbidity and mortality in Zambian children with cryptosporidiosis: A randomised controlled trial. Lancet.

[6] Gilbert IH, Vinayak S, Striepen B, Manjunatha UH, Khalil IA, Van Voorhis WC (2023). Safe and effective treatments are needed for cryptosporidiosis, a truly neglected tropical disease. BMJ Glob Health.

[7] Love MS, Choy RKM (2021). Emerging treatment options for cryptosporidiosis. Curr Opin Infect Dis.

[8] Baragaña B, Forte B, Choi R, Hewitt SN, Bueren-Calabuig JA, Pisco JP, Peet C, Dranow DM, Robinson DA, Jansen C, Norcross NR (2019). Lysyl-tRNA synthetase as a drug target in malaria and cryptosporidiosis. Proc Natl Acad Sci USA.

[9] Leitch GJ, He Q (2011). Cryptosporidiosis-an overview. J Biomed Res.

[10] Dorel R, Wong AR, Crawford JJ (2023). Trust Your Gut: Strategies and Tactics for Intestinally Restricted Drugs. ACS Med Chem Lett.

[11] Key Compound Progression Parameters Medicines for Malaria Venture.

[12] Hanna JC, Corpas-Lopez V, Seizova S, Colon BL, Bacchetti R, Hall GMJ, Sands EM, Robinson L, Baragaña B, Wyllie S, Pawlowic MC (2023). Mode of action studies confirm on-target engagement of lysyl-tRNA synthetase inhibitor and lead to new selection marker for Cryptosporidium. Front Cell Infect Microbiol.

[13] Amidon GL, Lennernas H, Shah VP, Crison JR (1995). A theoretical basis for a biopharmaceutic drug classification: the correlation of in vitro drug product dissolution and in vivo bioavailability. Pharm Res.

[14] Jumani RS, Bessoff K, Love MS, Miller P, Stebbins EE, Teixeira JE, Campbell MA, Meyers MJ, Zambriski JA, Nunez V, Woods AK (2018). A novel piperazine-based drug lead for cryptosporidiosis from the medicines for malaria venture open-access malaria box. Antimicrob Agents Chemother.

[15] English ED, Guerin A, Tandel J, Striepen B (2022). Live imaging of the Cryptosporidium parvum life cycle reveals direct development of male and female gametes from type I meronts. PLoS Biol.

[16] Manjunatha UH, Vinayak S, Zambriski JA, Chao AT, Sy T, Noble CG, Bonamy GMC, Kondreddi RR, Zou B, Gedeck P, Brooks CF (2017). A Cryptosporidium PI(4)K inhibitor is a drug candidate for cryptosporidiosis. Nature.

[17] Schaefer DA, Betzer DP, Smith KD, Millman ZG, Michalski HC, Menchaca SE, Zambriski JA, Ojo KK, Hulverson MA, Arnold SLM, Rivas KL (2016). Novel bumped kinase inhibitors are safe and effective therapeutics in the calf clinical model for cryptosporidiosis. J Inf Dis.

[18] Stebbins E, Jumani RS, Klopfer C, Barlow J, Miller P, Campbell MA, Meyers MJ, Griggs DW, Huston CD (2018). Clinical and microbiologic efficacy of the piperazine-based drug lead MMV665917 in the dairy calf cryptosporidiosis model. PLoS Negl Trop Dis.

[19] Hasan MM, Stebbins EE, Choy RKM, Gillespie JR, De Hostos EL, Miller P, Mushtaq A, Ranade RM, Teixeira JE, Verlinde CLMJ, Sateriale A (2021). Spontaneous Selection of Cryptosporidium Drug Resistance in a Calf Model of Infection. Antimicrob Agents Chemother.

[20] Milne R, Wiedemar N, Corpas-Lopez V, Moynihan E, Wall RJ, Dawson A, Robinson DA, Shepherd SM, Smith RJ, Hallyburton I, Post JM (2022). Toolkit of Approaches To Support Target-Focused Drug Discovery for Plasmodium falciparum Lysyl tRNA Synthetase. ACS Infect Dis.

[21] Huston CD, Spangenberg T, Burrows J, Willis P, Wells TNC, Van Voorhis W (2015). A Proposed Target Product Profile and Developmental Cascade for New Cryptosporidiosis Treatments. PLoS Negl Trop Dis.

[22] Jumani RS, Blais J, Tillmann HC, Segal F, Wetty D, Ostermeier C, Nuber N, Lakshman J, Aziz N, Chandra R, Chen WH (2021). Opportunities and Challenges in Developing a Cryptosporidium Controlled Human Infection Model for Testing Antiparasitic Agents. ACS Infect Dis.

[23] Vinayak S, Jumani RS, Miller P, Hasan MM, McLeod BI, Tandel J, Stebbins EE, Teixeira JE, Borrel J, Gonse A, Zhang M (2020). Bicyclic azetidines kill the diarrheal pathogen Cryptosporidium in mice by inhibiting parasite phenylalanyl-tRNA synthetase. Sci Transl Med.

[24] Arnold SLM, Choi R, Hulverson MA, Schaefer DA, Vinayak S, Vidadala RSR, McCloskey MC, Whitman GR, Huang W, Barrett LK, Ojo KK (2017). Necessity of Bumped Kinase Inhibitor Gastrointestinal Exposure in Treating Cryptosporidium Infection. J Inf Dis.

[25] De Rycker M, Horn D, Aldridge B, Amewu RK, Barry CE, Buckner FS, Cook S, Ferguson MAJ, Gobeau N, Herrmann J, Herrling P (2020). Setting Our Sights on Infectious Diseases. ACS Infect Dis.

[26] Green SR, Davis SH, Damerow S, Engelhart CA, Mathieson M, Baragaña B, Robinson DA, Tamjar J, Dawson A, Tamaki FK, Buchanan KI (2022). Lysyl-tRNA synthetase, a target for urgently needed M. tuberculosis drugs. Nat Commun.

[27] Murugesan D, Ray PC, Bayliss T, Prosser GA, Harrison JR, Green K, Soares De Melo C, Feng TS, Street LJ, Chibale K, Warner DF (2018). 2-Mercapto-Quinazolinones as Inhibitors of Type II NADH Dehydrogenase and Mycobacterium tuberculosis: Structure-Activity Relationships, Mechanism of Action and Absorption, Distribution, Metabolism, and Excretion Characterization. ACS Infect Dis.

[28] Cai X, Woods KM, Upton SJ, Zhu G (2005). Application of quantitative real-time reverse transcription-PCR in assessing drug efficacy against the intracellular pathogen Cryptosporidium parvum in vitro. Antimicrob Agents Chemother.

[29] Bessoff K, Sateriale A, Lee KK, Huston CD (2013). Drug repurposing screen reveals FDA-approved inhibitors of human HMG-CoA reductase and isoprenoid synthesis that block Cryptosporidium parvum growth. Antimicrob Agents Chemother.

[30] Thomas MG, De Rycker M, Ajakane M, Albrecht S, Álvarez-Pedraglio AI, Boesche M, Brand S, Campbell L, Cantizani-Perez J, Cleghorn LAT, Copley RCB (2019). Identification of GSK3186899/DDD853651 as a Preclinical Development Candidate for the Treatment of Visceral Leishmaniasis. J Med Chem.

[31] Baragaña B, Hallyburton I, Lee MCS, Norcross NR, Grimaldi R, Otto TD, Proto WR, Blagborough AM, Meister S, Wirjanata G, Ruecker A (2015). A novel multiple-stage antimalarial agent that inhibits protein synthesis. Nature.

[32] Pawlowic MC, Vinayak S, Sateriale A, Brooks CF, Striepen B (2017). Generating and maintaining transgenic cryptosporidium parvum parasites. Curr Protoc Microbiol.

[33] Sateriale A, Šlapeta J, Baptista R, Engiles JB, Gullicksrud JA, Herbert GT, Brooks CF, Kugler EM, Kissinger JC, Hunter CA, Striepen A (2019). Natural Mouse Model of Cryptosporidiosis Offers Insights into Host Protective Immunity. Cell Host Microbe.

[34] Tyanova S, Temu T, Cox J (2016). The MaxQuant computational platform for mass spectrometry-based shotgun proteomics. Nat Protoc.

[35] Warrenfeltz S, Kissinger JC, Mead JR, Arrowood MJ (2020). Cryptosporidium: Methods and Protocols, Methods in Molecular Biology.

[36] Zaru R, Orchard S (2023). UniProt Tools: BLAST, Align, Peptide Search, and ID Mapping. Curr Protoc.

[37] Plubell DL, Wilmarth PA, Zhao Y, Fenton AM, Minnier J, Reddy AP, Klimek J, Yang X, David LL, Pamir N (2017). Extended multiplexing of tandem mass tags (TMT) labeling reveals age and high fat diet specific proteome changes in mouse epididymal adipose tissue. Molecular and Cellular Proteomics.

[38] Ritchie ME, Phipson B, Wu D, Hu Y, Law CW, Shi W, Smyth GK (2015). Limma powers differential expression analyses for RNA-sequencing and microarray studies. Nucleic Acids Res.

[39] Gilbert IH, Baragana B, Caldwell N, Taylor M, Forte B, Cocco M, Jansen C (2023). Anti-infective agents.

[40] Forte B, Norcross N, Jansen C, Baragana B, Gilbert I, Cleghorn L, Davis S, Walpole C (2017). Anti-infective agents.

[41] Kabsch W (2010). XDS. Acta Crystallogr D Biol Crystallogr.

[42] Winter G (2010). xia2: an expert system for macromolecular crystallography data reduction. J Appl Crystallogr.

[43] Evans PR, Murshudov GN (2013). How good are my data and what is the resolution?. Acta Crystallogr D Biol Crystallogr.

[44] McCoy AJ, Grosse-Kunstleve RW, Adams PD, Winn MD, Storoni LC, Read RJ (2007). Phaser crystallographic software. J Appl Crystallogr.

[45] Emsley P, Lohkamp B, Scott WG, Cowtan K (2010). Features and development of Coot. Acta Crystallogr D Biol Crystallogr.

[46] Murshudov GN, Skubák P, Lebedev AA, Pannu NS, Steiner RA, Nicholls RA, Winn MD, Long F, Vagin AA (2011). REFMAC5 for the refinement of macromolecular crystal structures. Acta Crystallogr D Biol Crystallogr.

[47] Winn MD, Ballard CC, Cowtan KD, Dodson EJ, Emsley P, Evans PR, Keegan RM, Krissinel EB, Leslie AGW, McCoy A, McNicholas SJ (2011). Overview of the CCP4 suite and current developments. Acta Crystallogr D Biol Crystallogr.

[48] Long F, Nicholls RA, Emsley P, Gražulis S, Merkys A, Vaitkus A, Murshudov GN (2017). AceDRG: A stereochemical description generator for ligands. Acta Crystallogr D Struct Biol.

[49] Williams CJ, Headd JJ, Moriarty NW, Prisant MG, Videau LL, Deis LN, Verma V, Keedy D, Hintze BJ, Chen VB, Jain S (2018). MolProbity: More and better reference data for improved all-atom structure validation. Protein Science.

