# Supplementary material for: Cryptosporidium lysyl-tRNA synthetase inhibitors define the interplay between solubility and permeability required to achieve efficacy
Source: Sci Transl Med. Author manuscript; Available in PMC 2025 Mar 4. (PMC7617456; doi:10.1126/scitranslmed.adm8631)
Supplement: Compound Spectra [file EMS199745-supplement-Compound_Spectra.pdf]

2-Amino-6-(cyclohexylmethyl)-7H-pyrrolo[3,4-d]pyrimidin-5-one (DDD212)

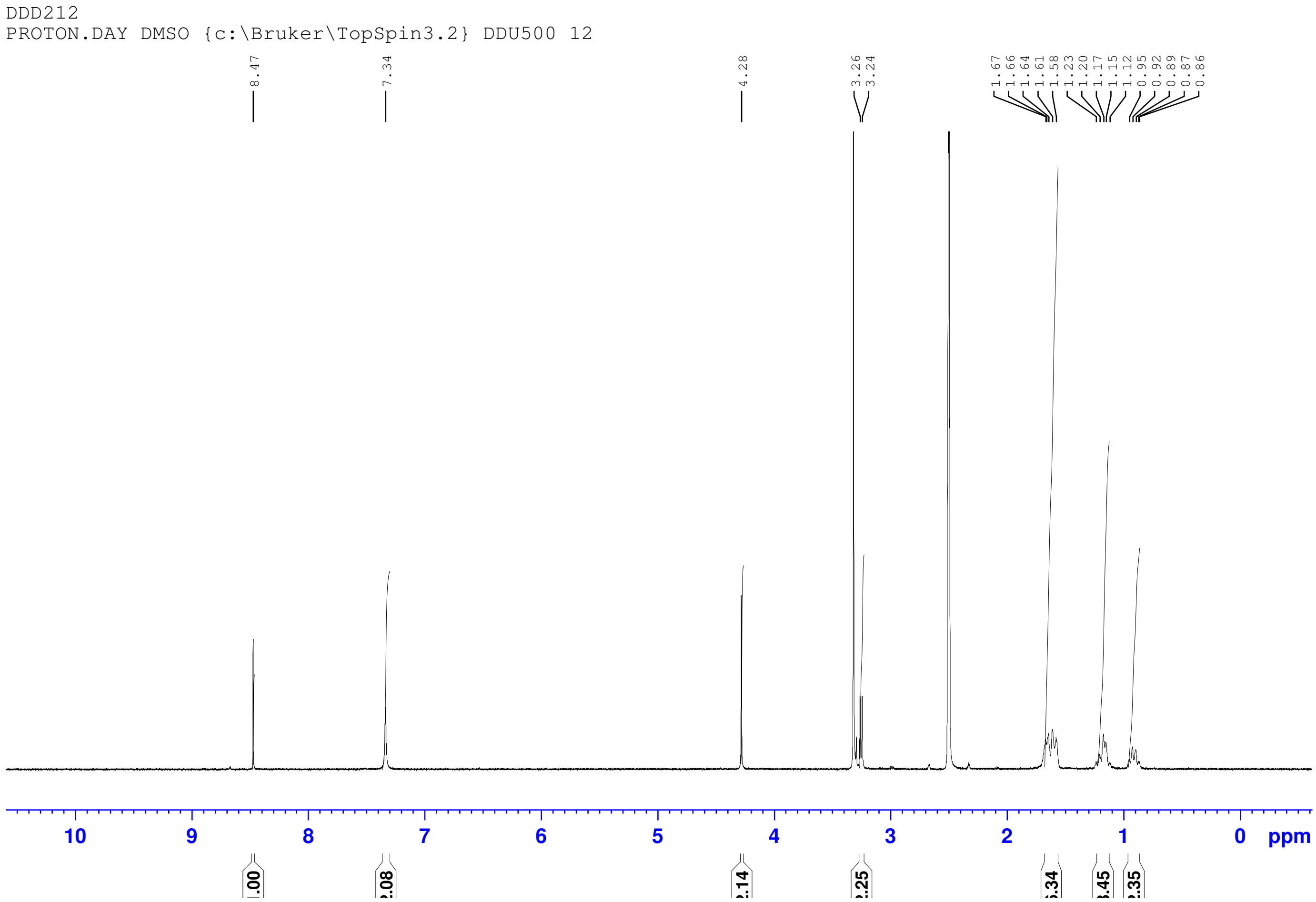

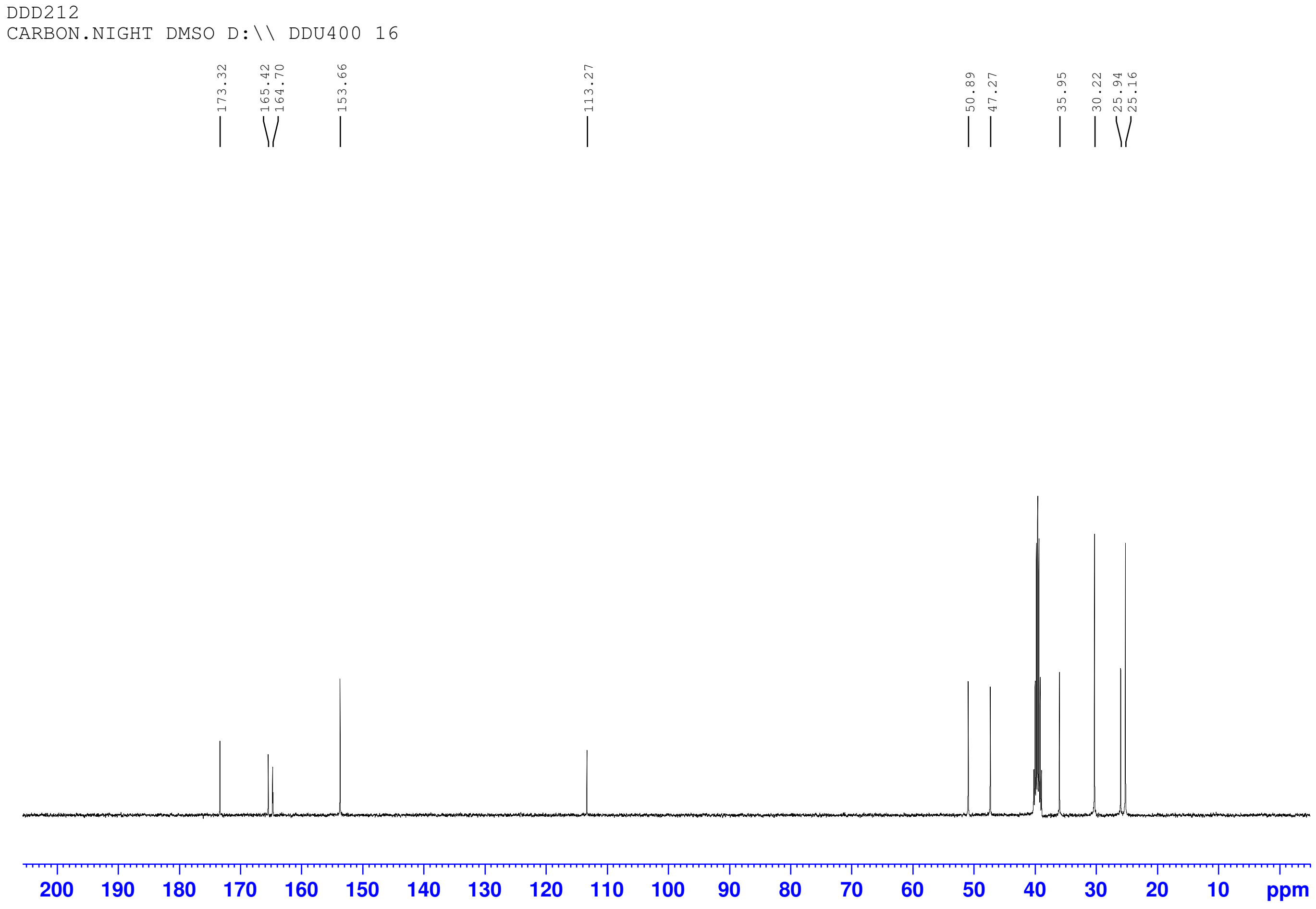

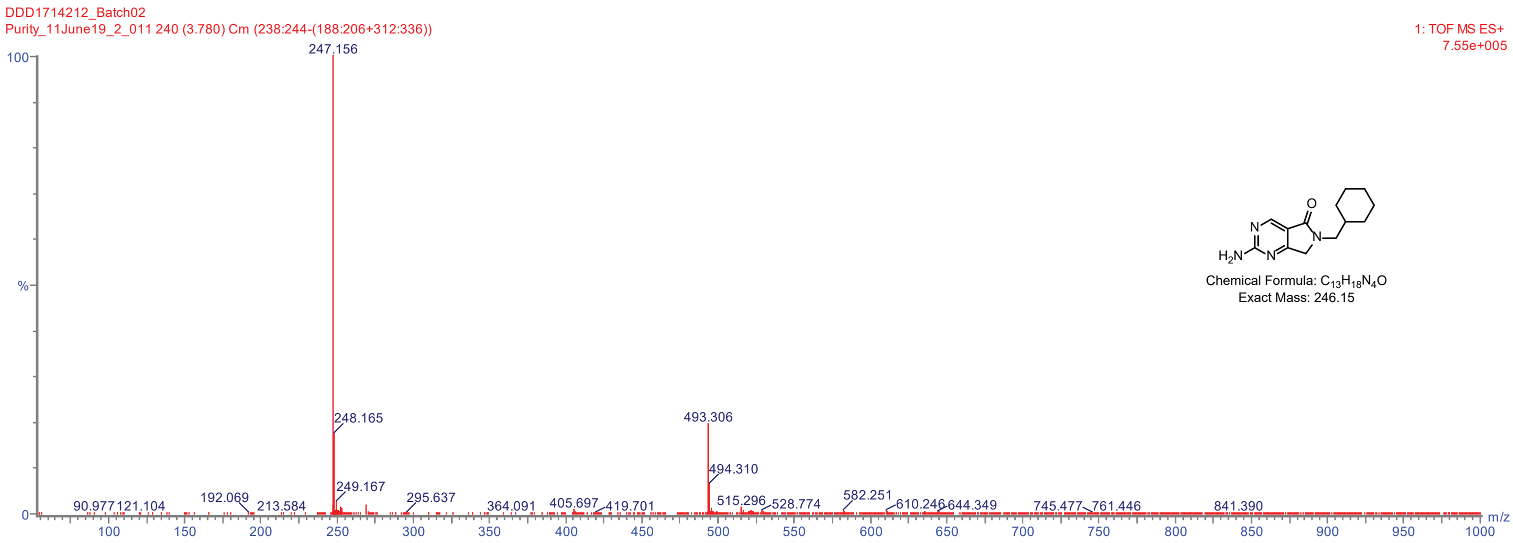

7-fluoro-N-((1-fluorocyclohexyl)methyl)-8-hydroxy-4-oxo-4H-chromene-2-carboxamide (DDD993)

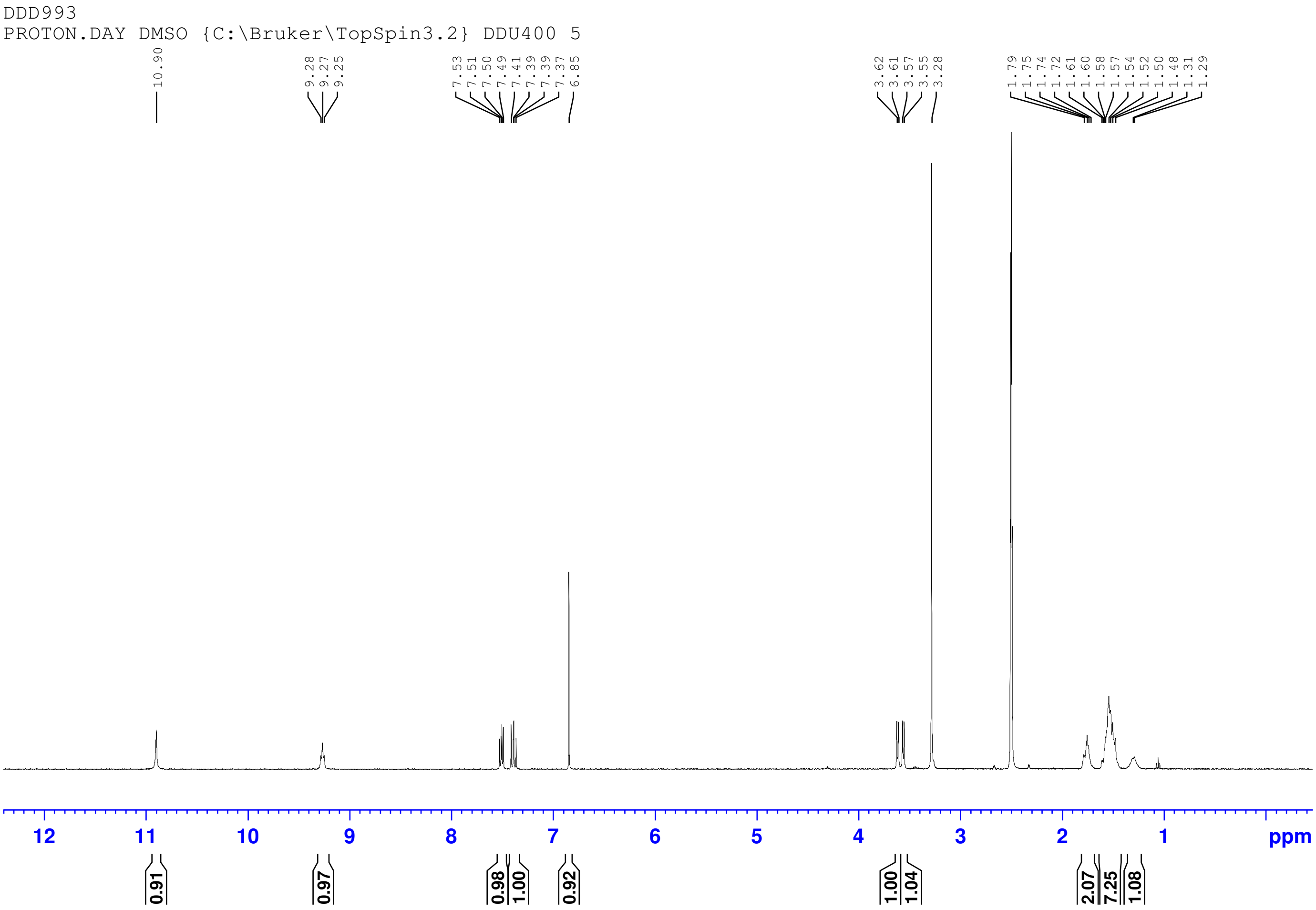

DDD993
CARBON.NIGHT DMSO {C:\Bruker\TopSpin3.2} DDU400 47

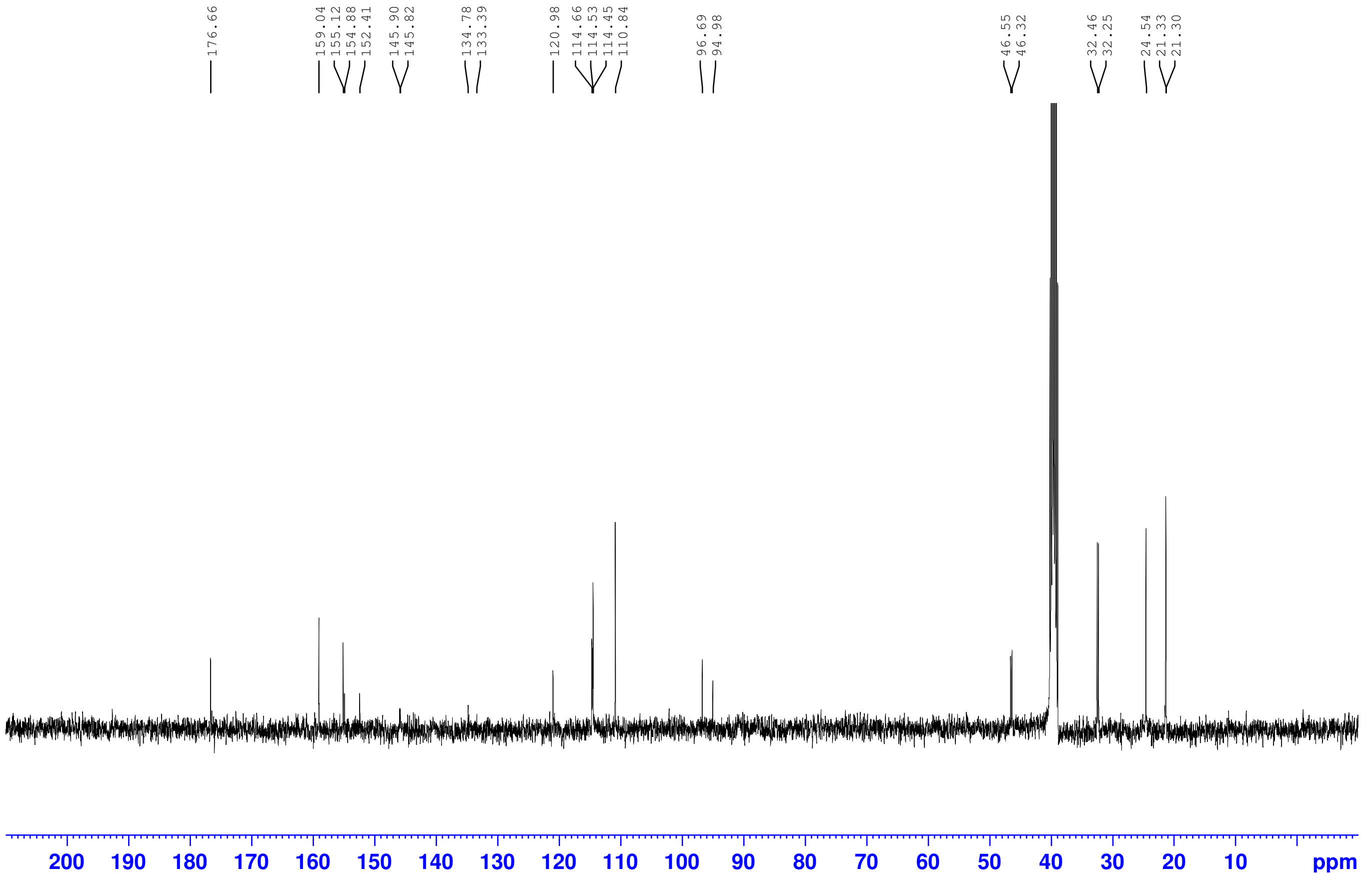

DDD993
F19CPD.DAY DMSO {D:\nmrdata} DDU500 2

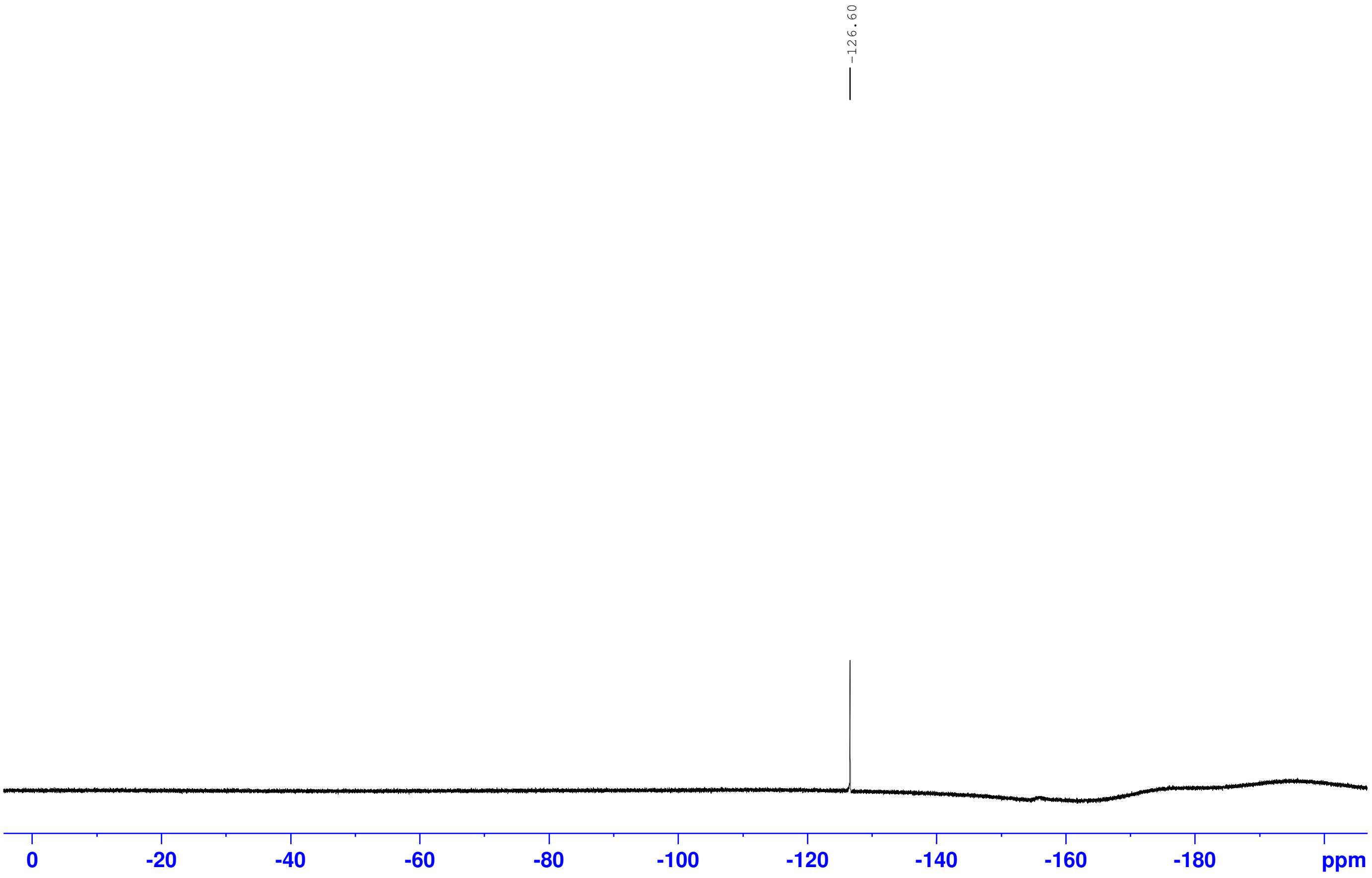

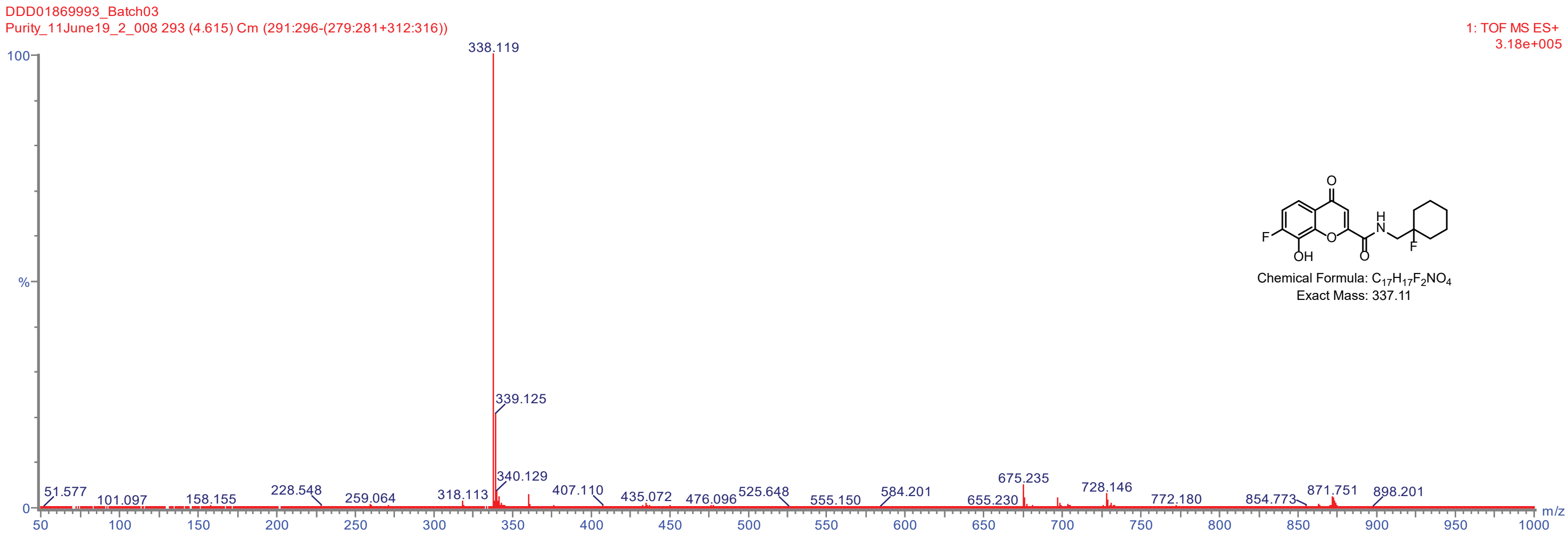

N-(cyclohexylmethyl)-7-fluoro-8-hydroxy-4-oxo-4H-chromene-2-carboxamide (DDD714)

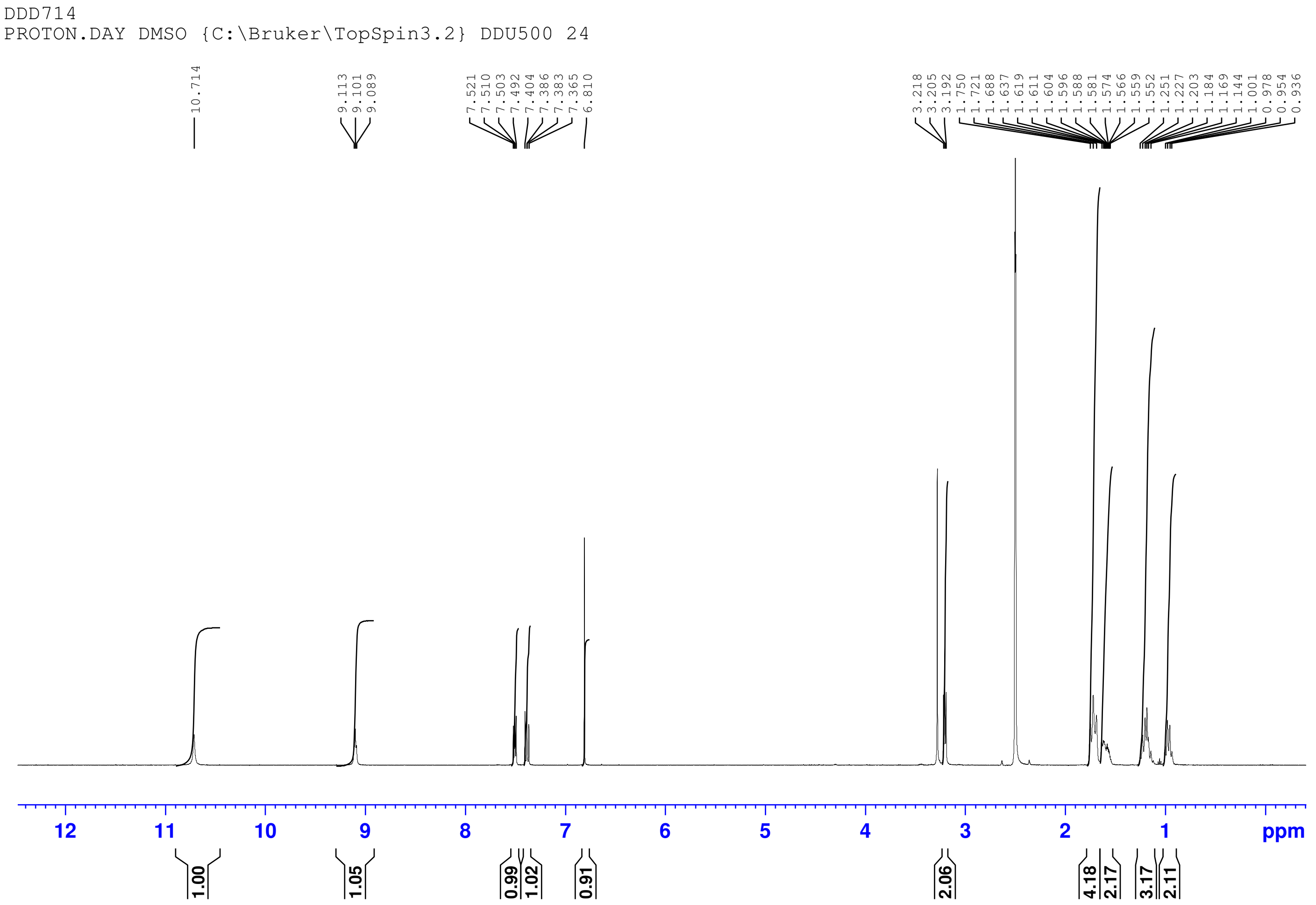

DDD714
CARBON.NIGHT DMSO {C:\Bruker\TopSpin3.2} DDU500 13

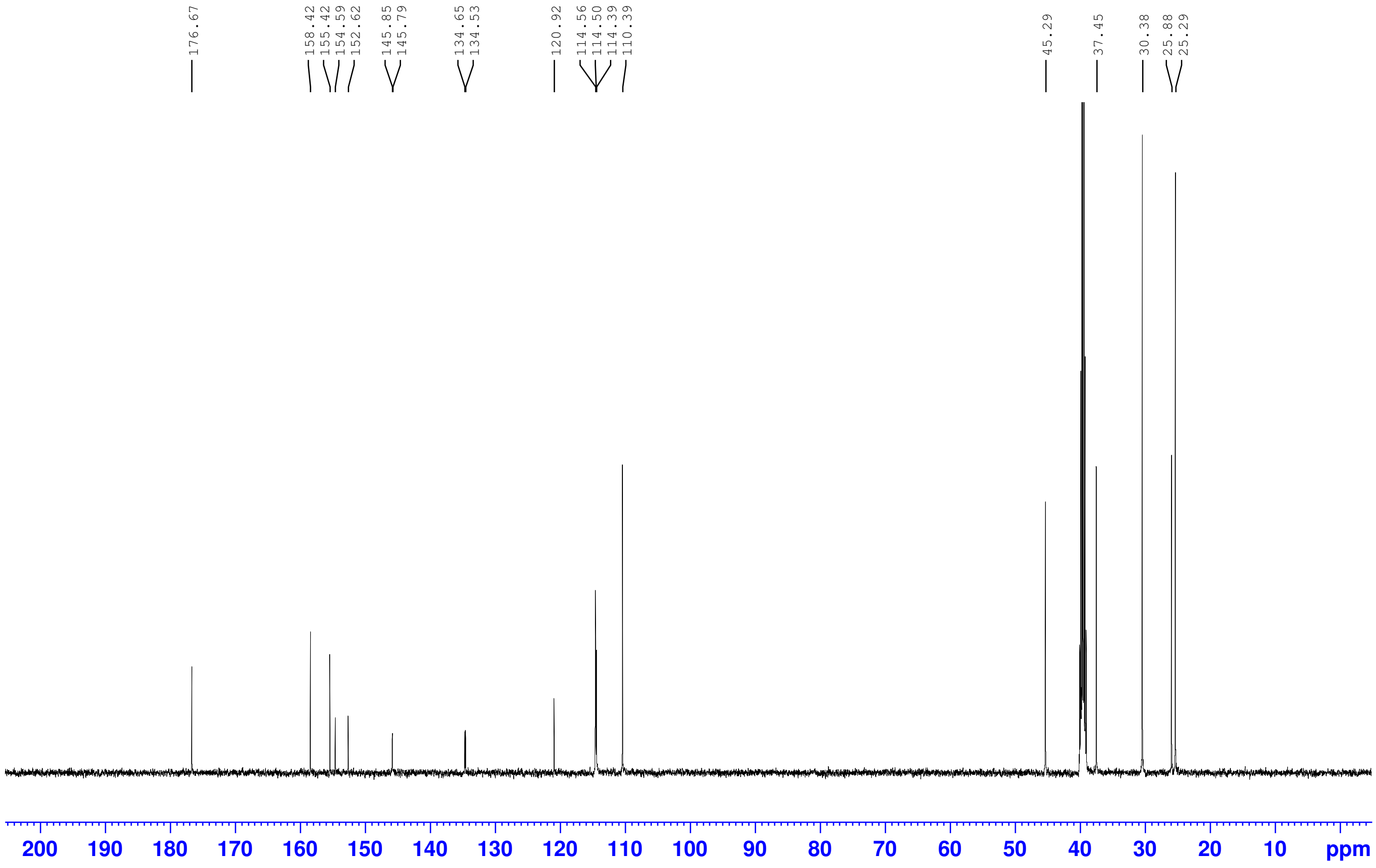

DDD714
F19.DAY DMSO {C:\Bruker\TopSpin3.2} DDU500 13

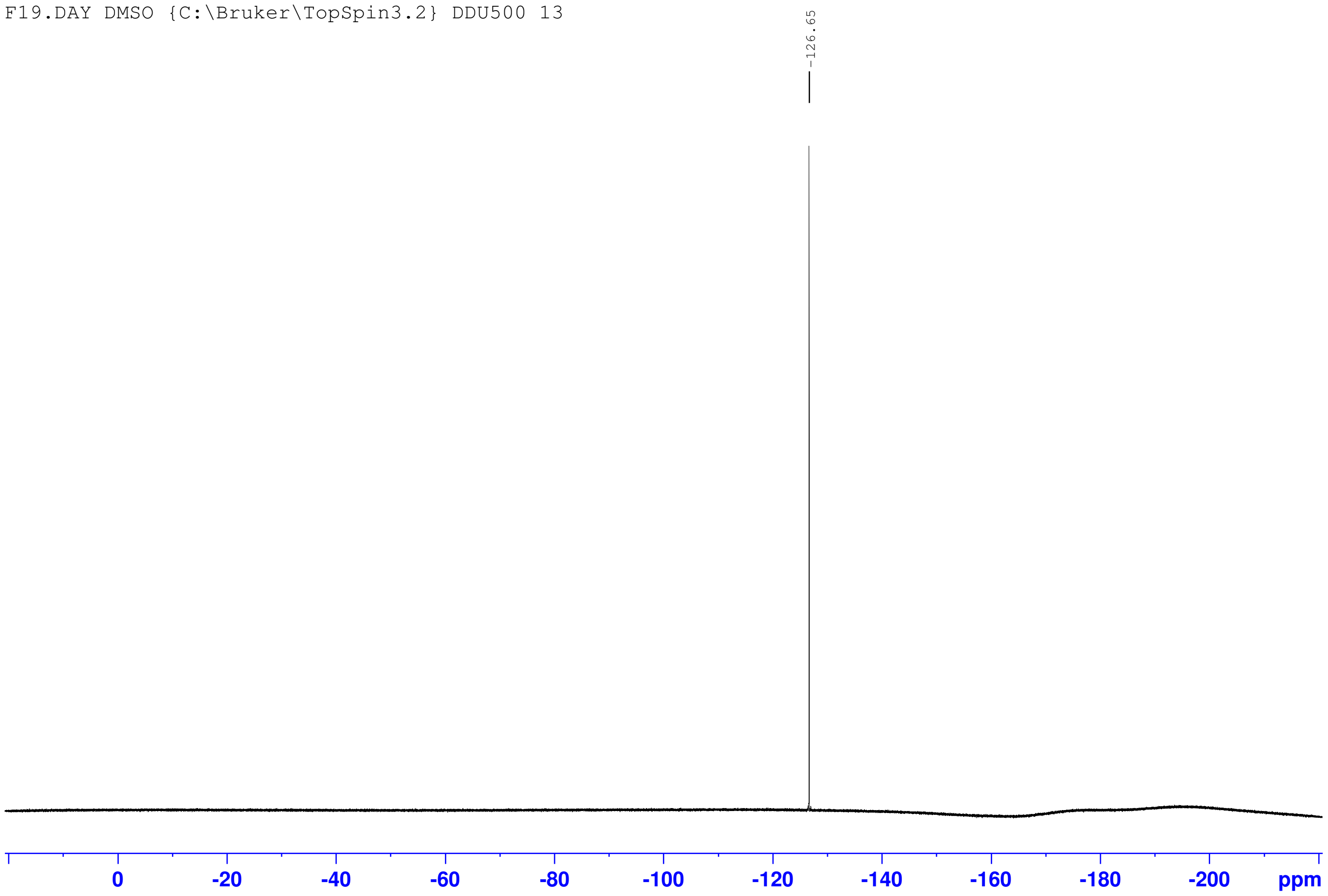

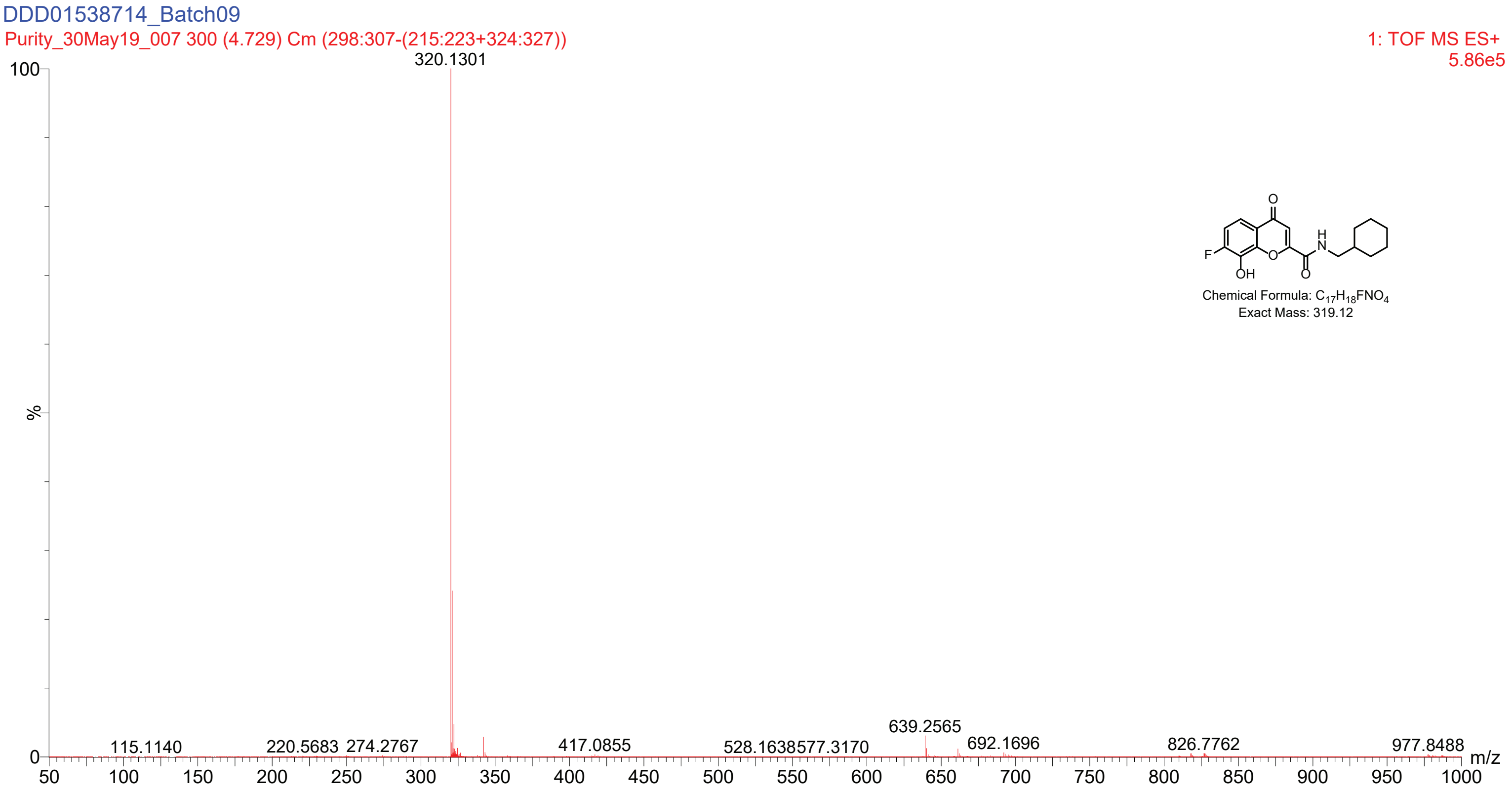

N-(cyclohexylmethyl)-6,7-difluoro-8-hydroxy-4-oxo-4H-chromene-2-carboxamide (DDD690)

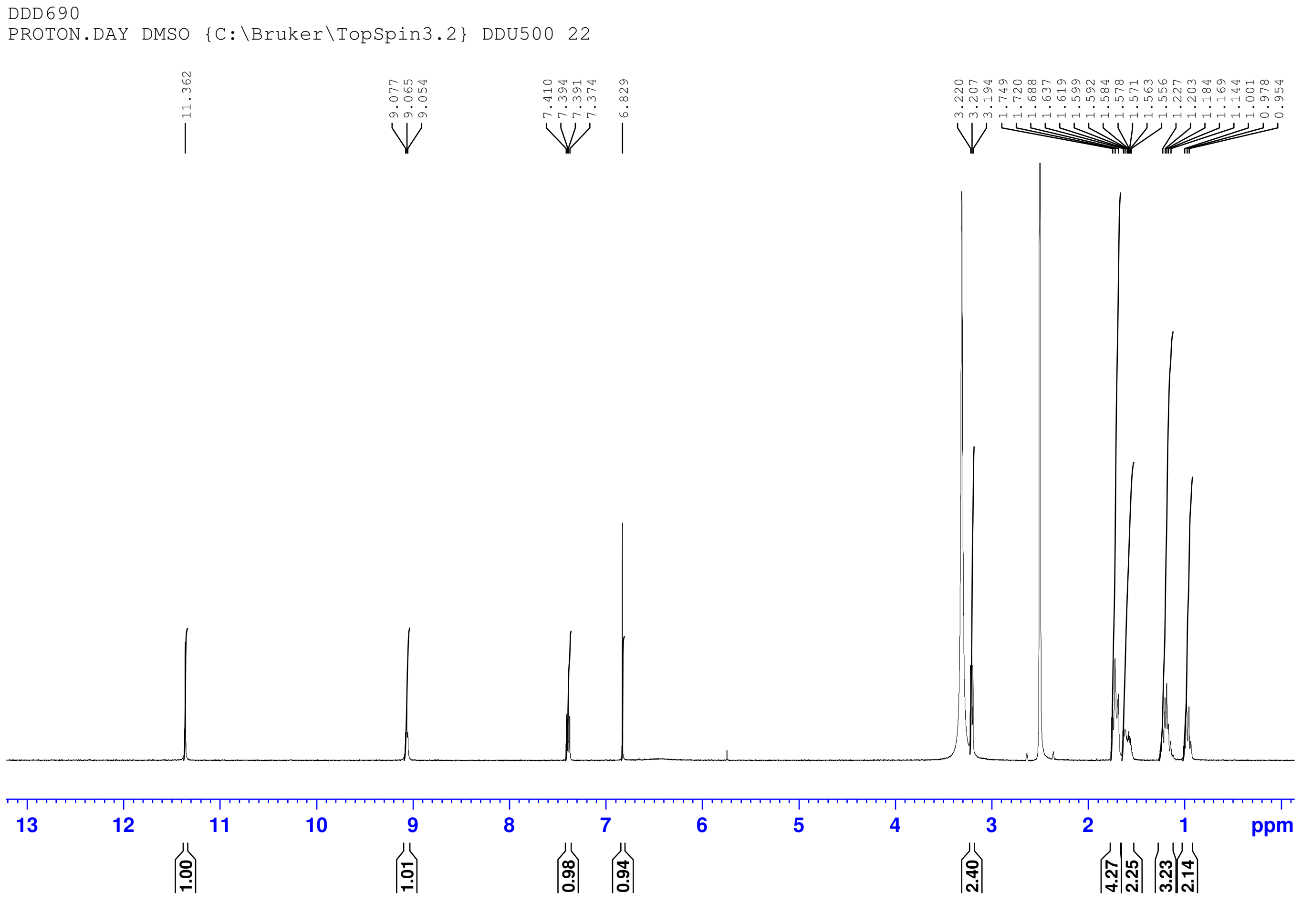

DDD690
CARBON.DAY MeOD {C:\Bruker\TopSpin3.2} DDU500 9

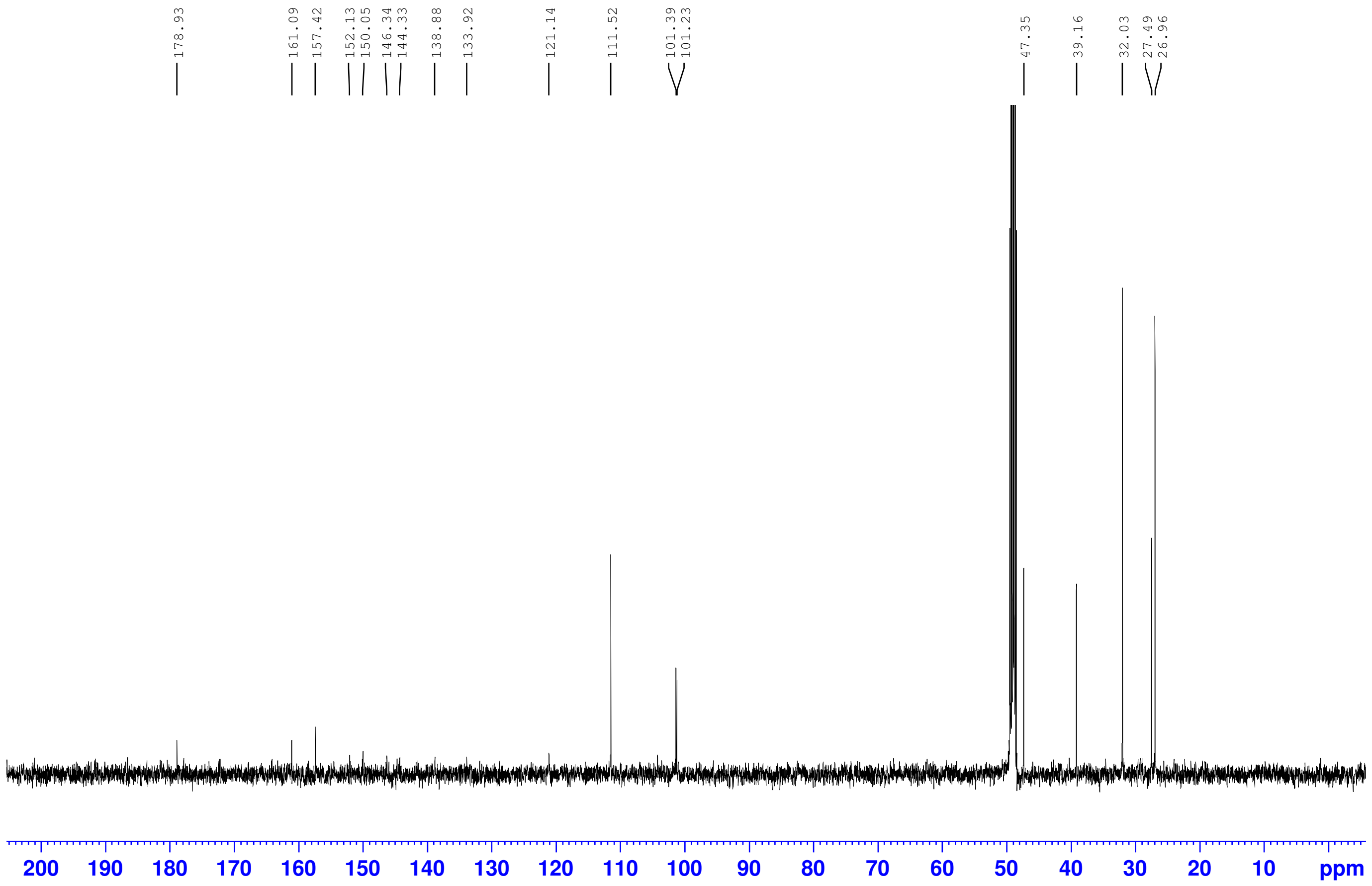

DDD690
F19CPD.DAY DMSO {D:\nmrdata} DDU500 5

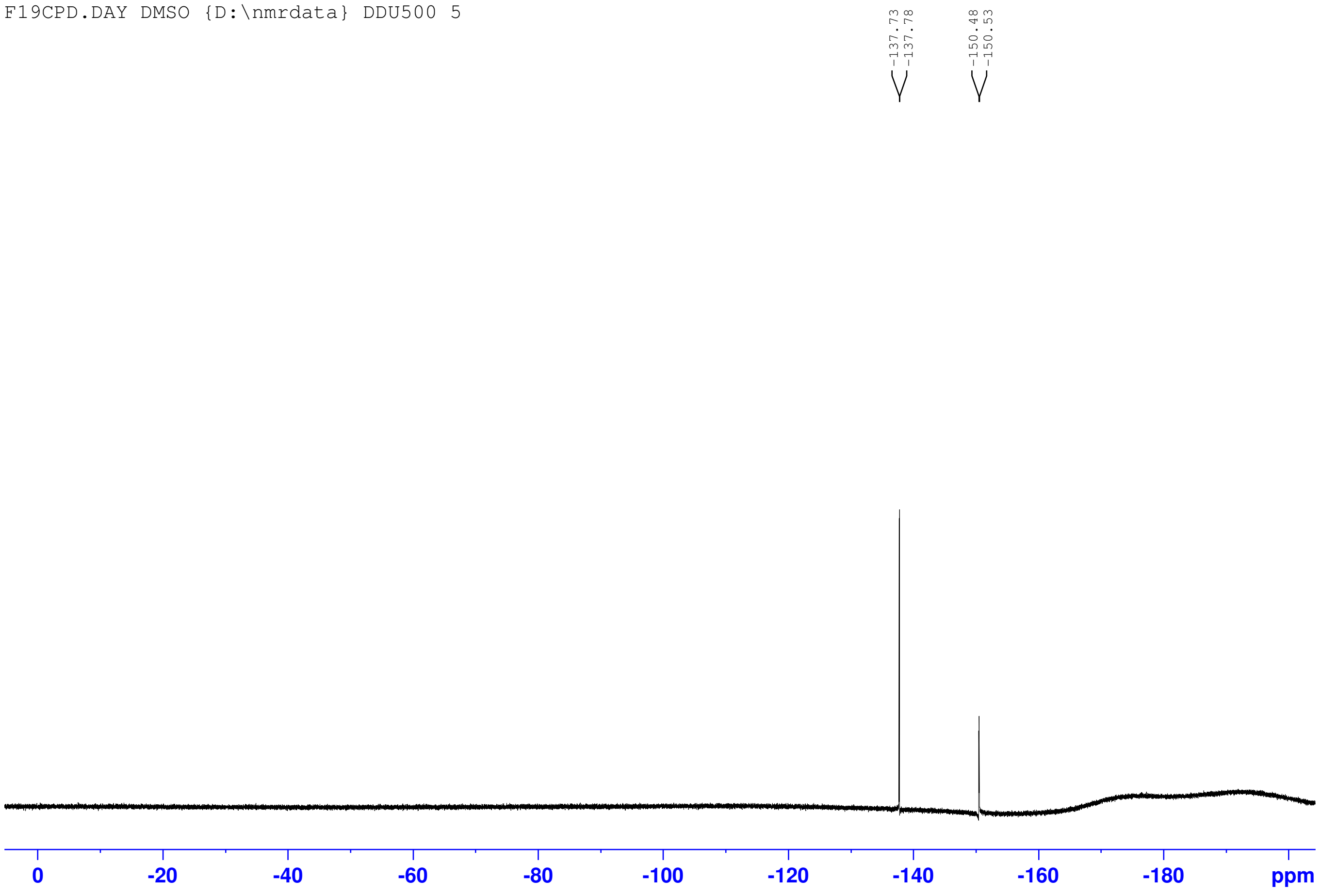

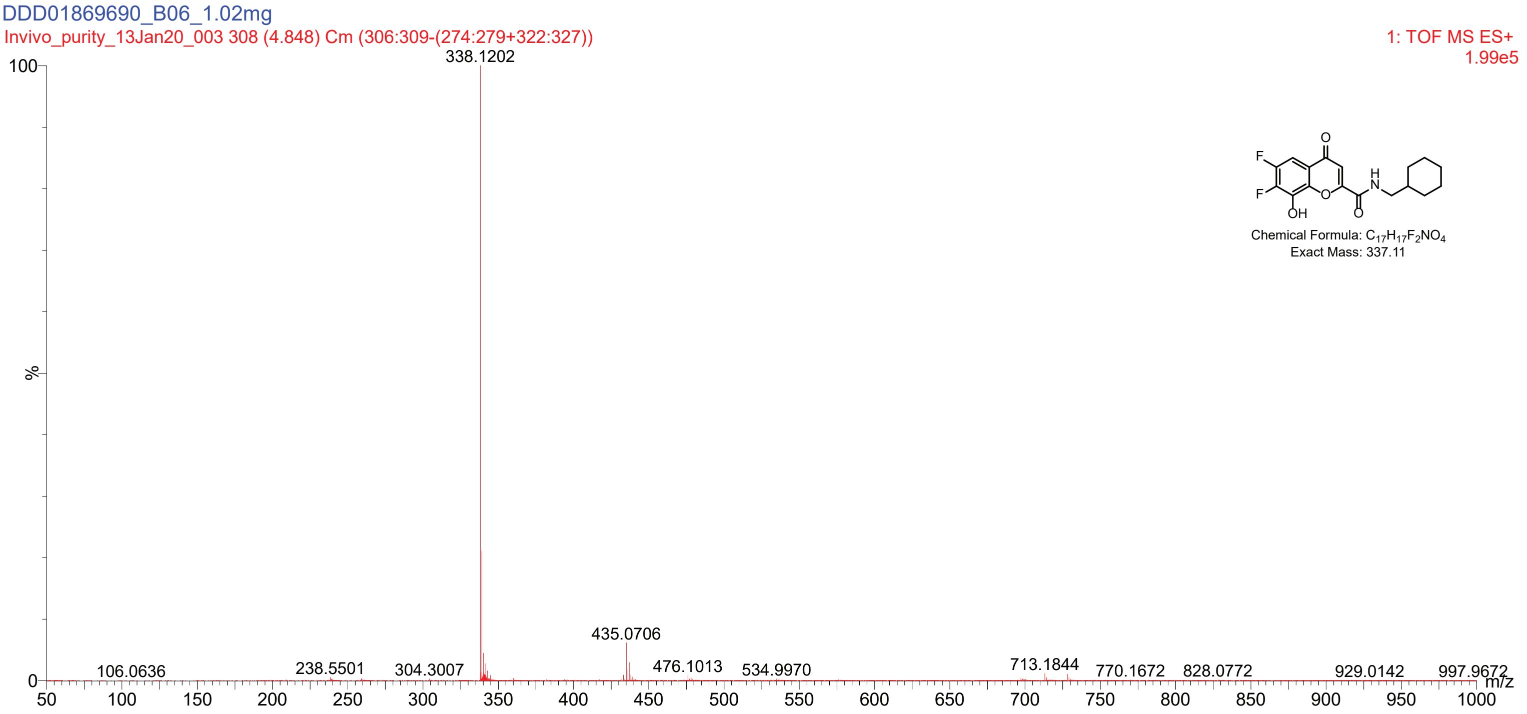

2-amino-6-(spiro[2.5]octan-5-ylmethyl)-4-(trifluoromethyl)-6,7-dihydro-5H-pyrrolo[3,4-d]pyrimidin-5-one (DDD909)

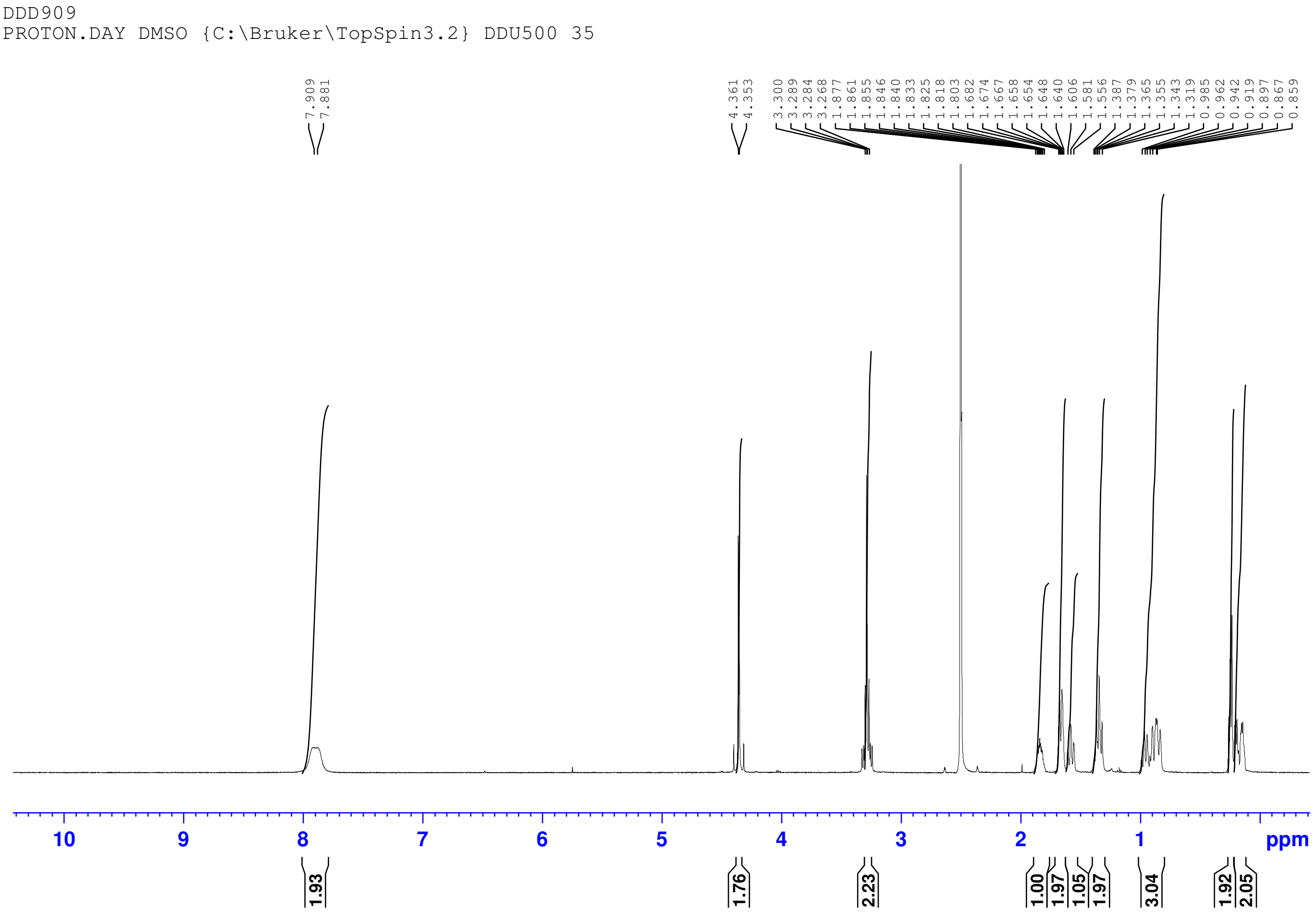

DDD909
CARBON.NIGHT DMSO D:\\ DDU400 15

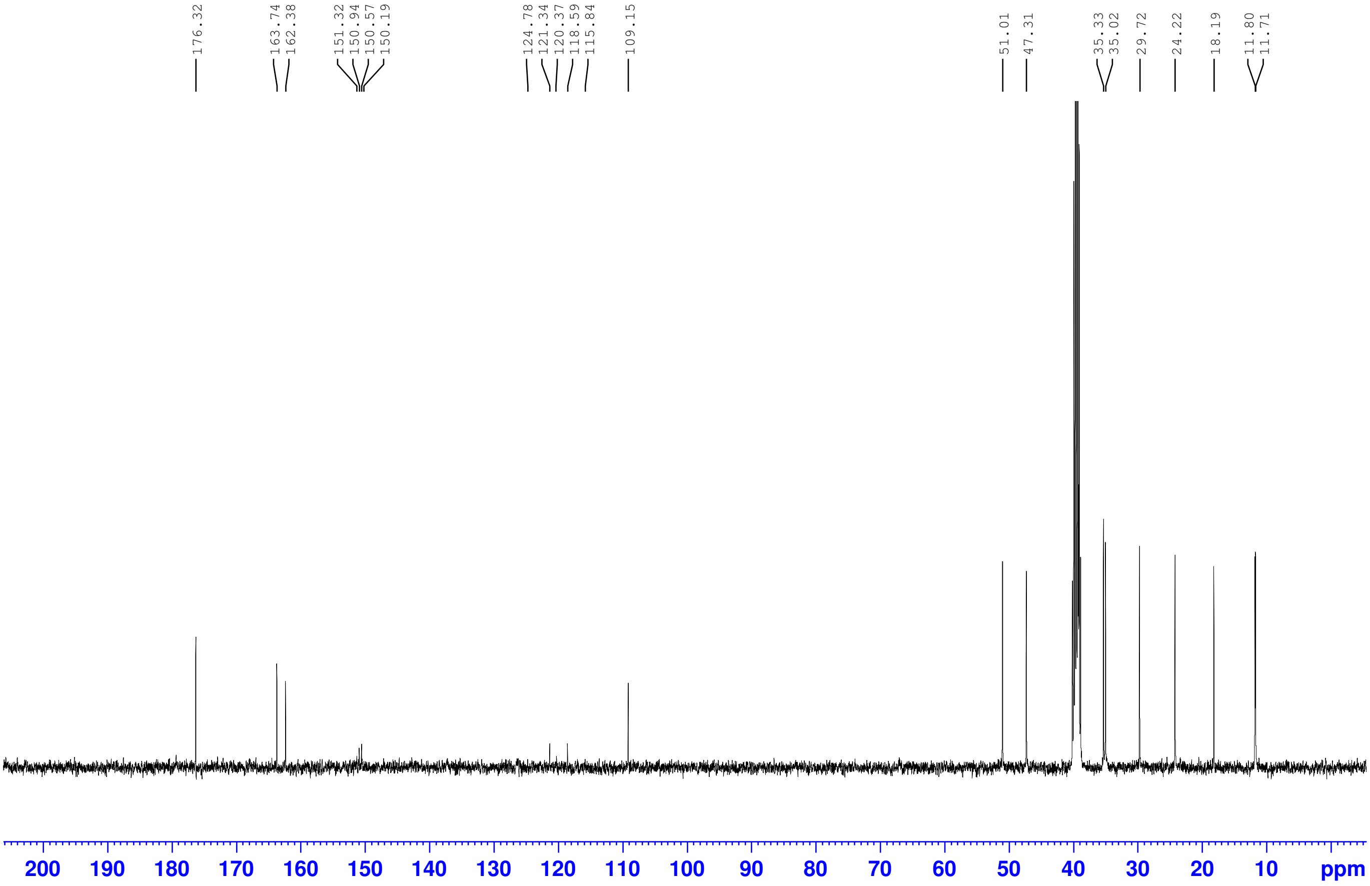

DDD909
F19.DAY DMSO {D:\nmrdata} DDU500 2

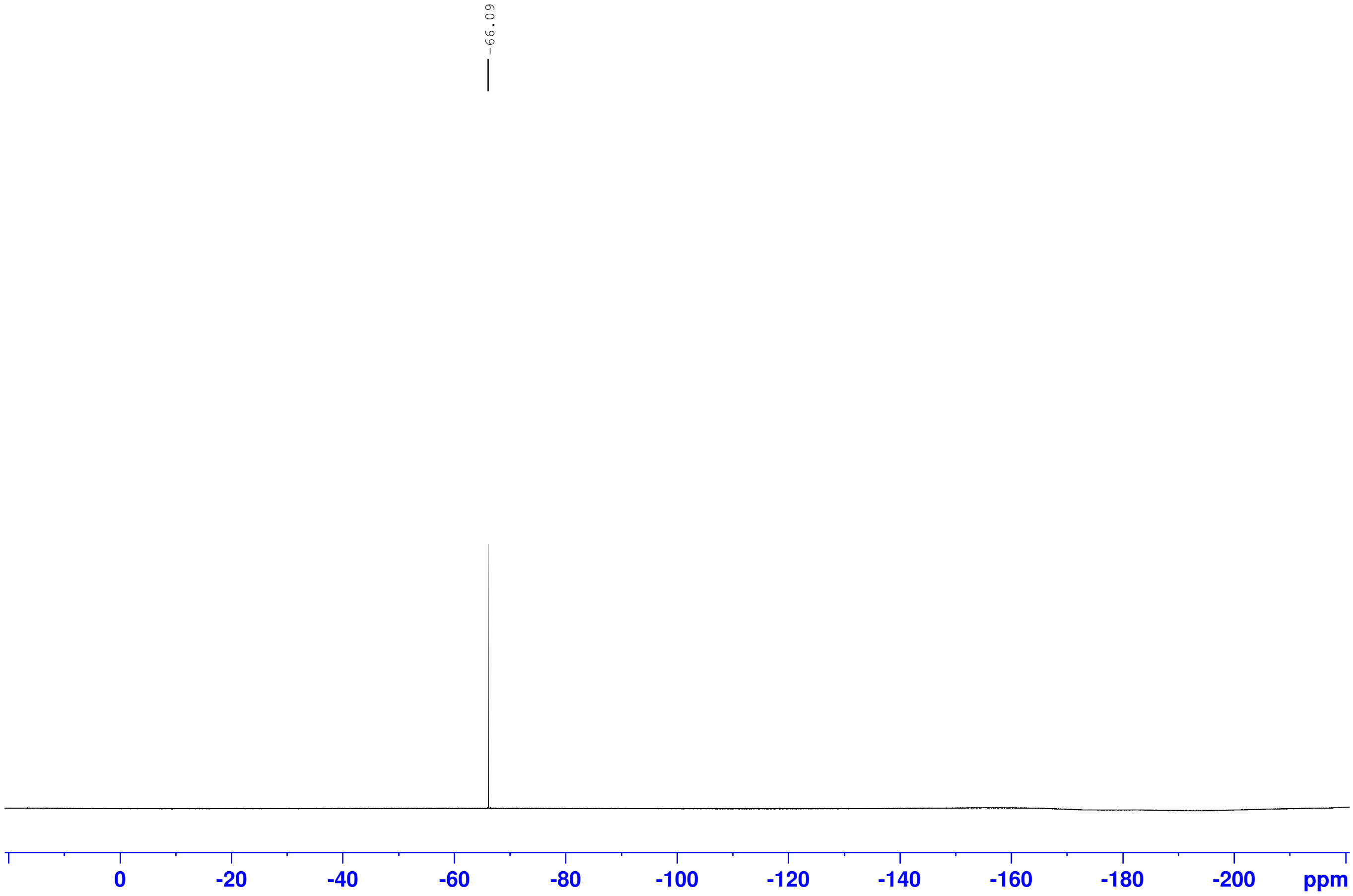

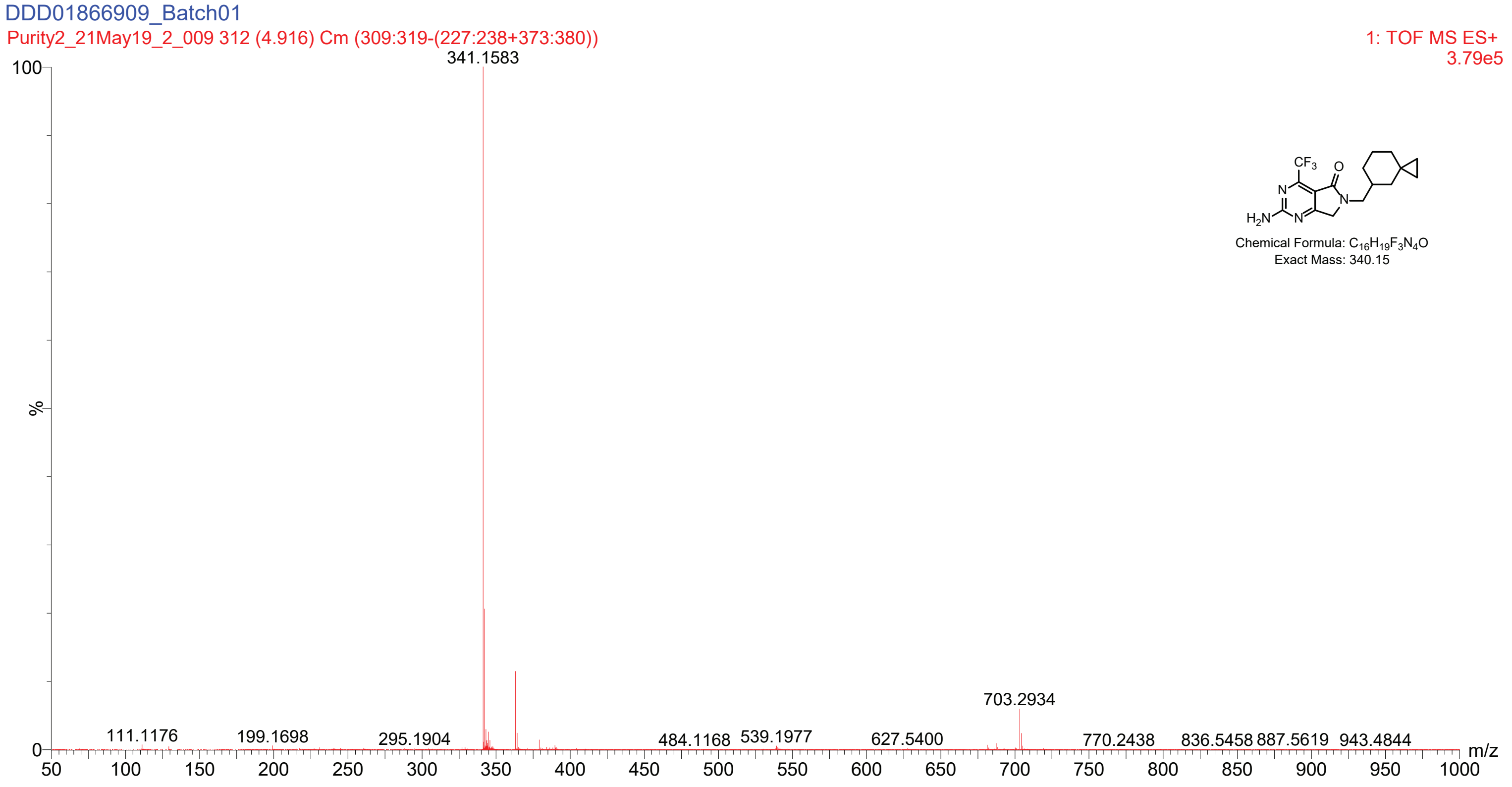

2-amino-4-(trifluoromethyl)-6-(((1R,3S)-3-(trifluoromethyl)cyclohexyl)methyl)-6,7-dihydro-5H-pyrrolo[3,4-d] pyrimidin-5-one (DDD489)

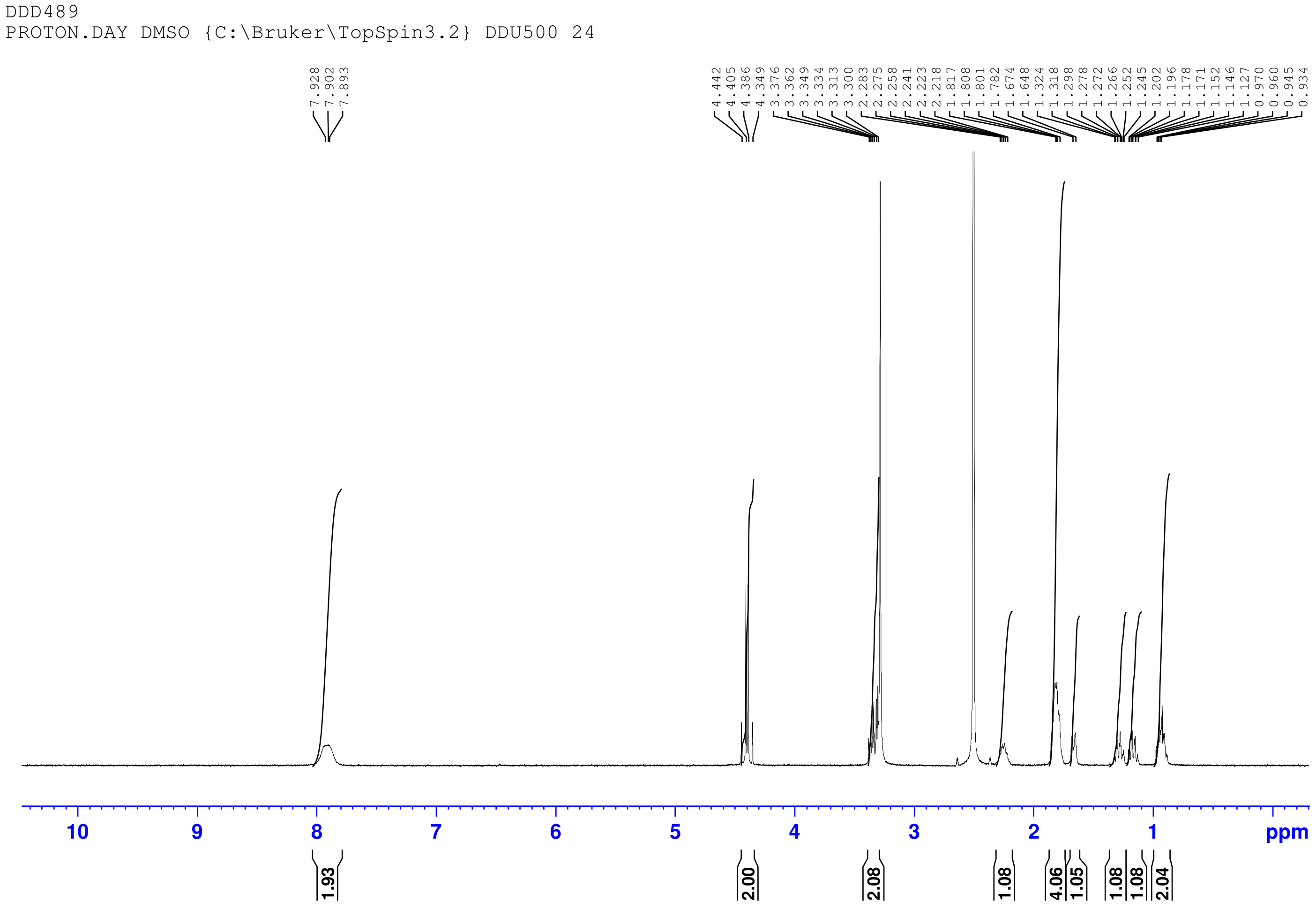

DDD489
CARBON.NIGHT DMSO {D:\nmrdata} DDU500 7

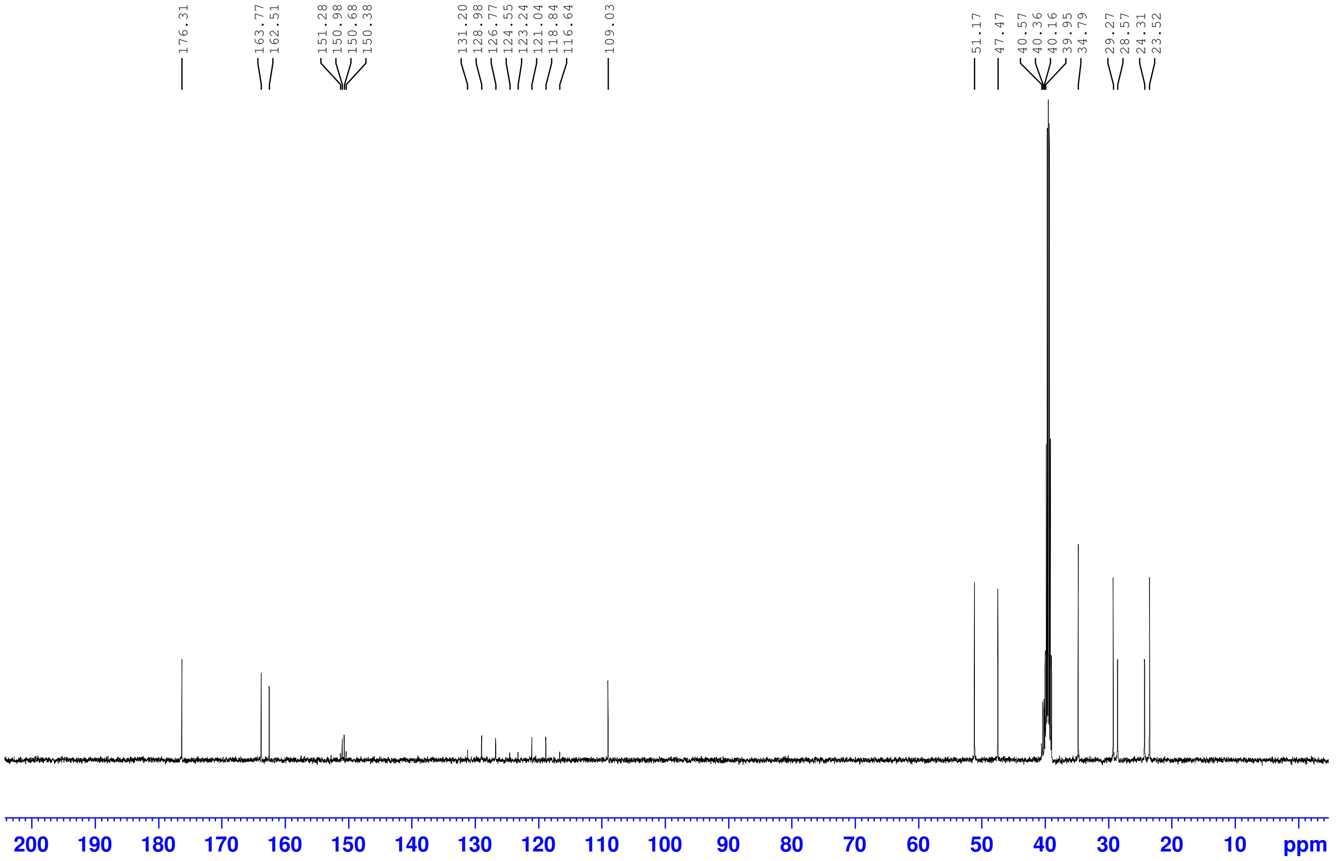

DDD489
F19CPD.DAY DMSO {D:\nmrdata} DDU500 7

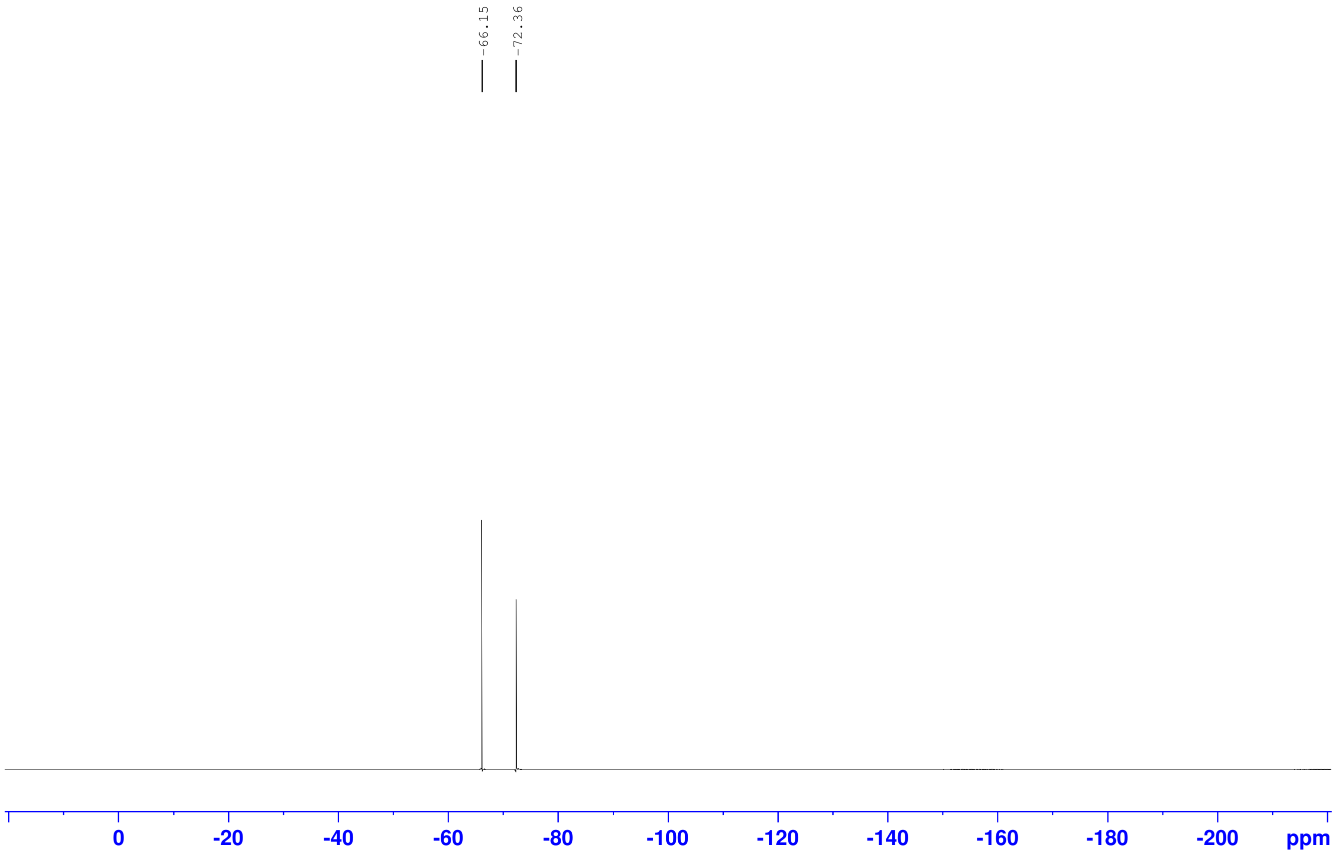

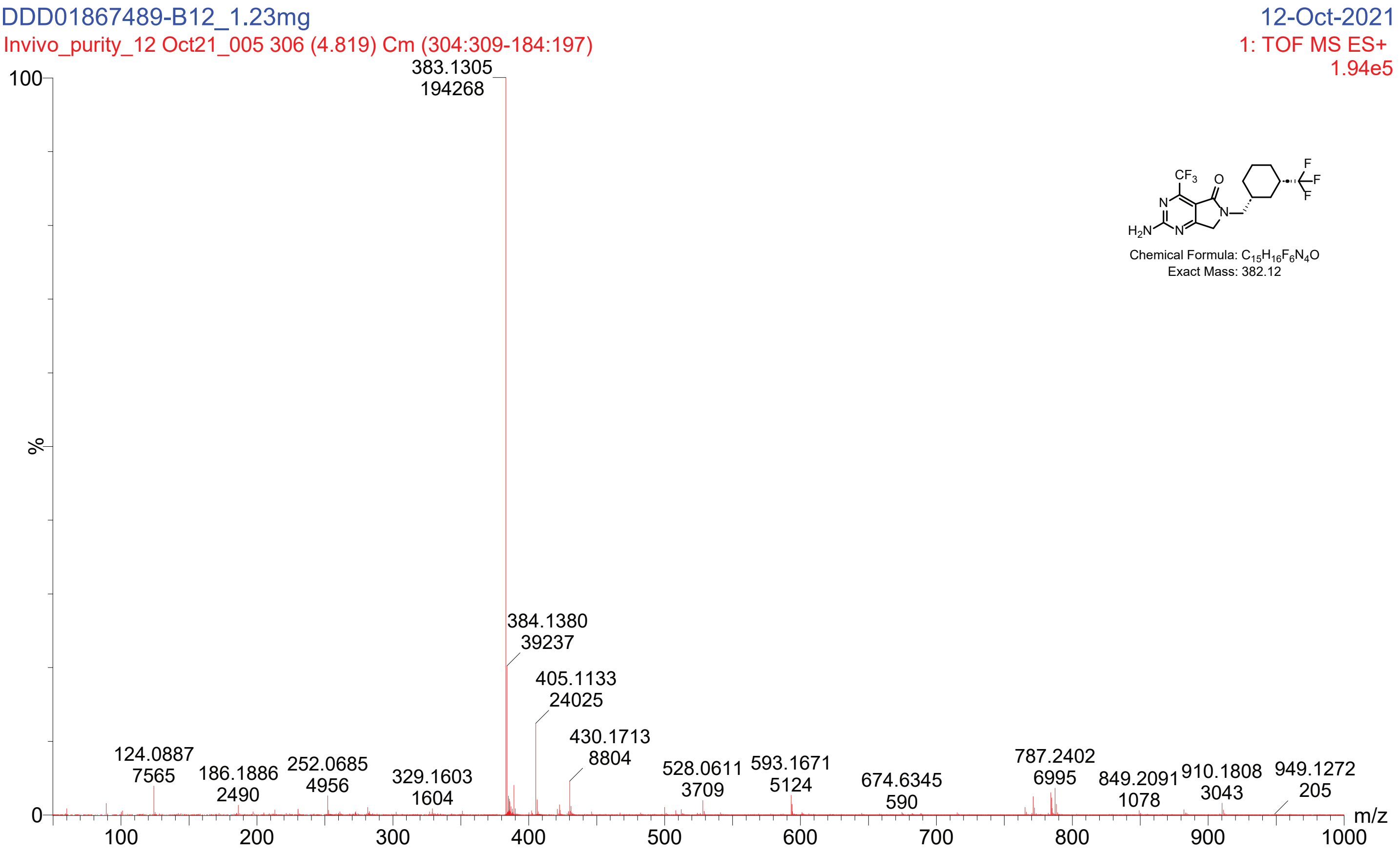

2-amino-4-(difluoromethyl)-6-(((1R,3S)-3-(trifluoromethyl)cyclohexyl)methyl)-6,7-dihydro-5H-pyrrolo[3,4-d] pyrimidin-5-one (DDD352)

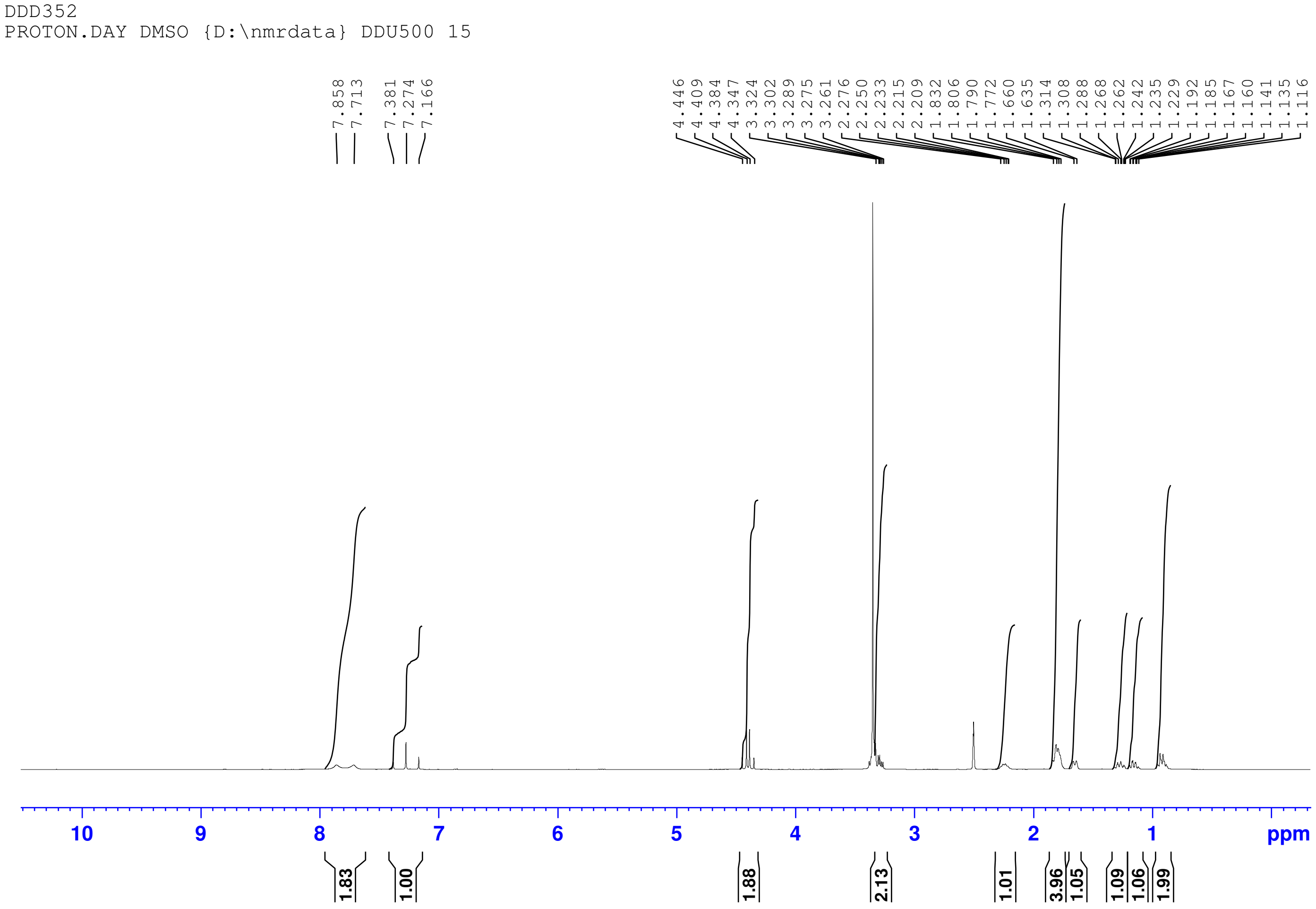

DDD352
CARBON.NIGHT DMSO D:\\ DDU400 18

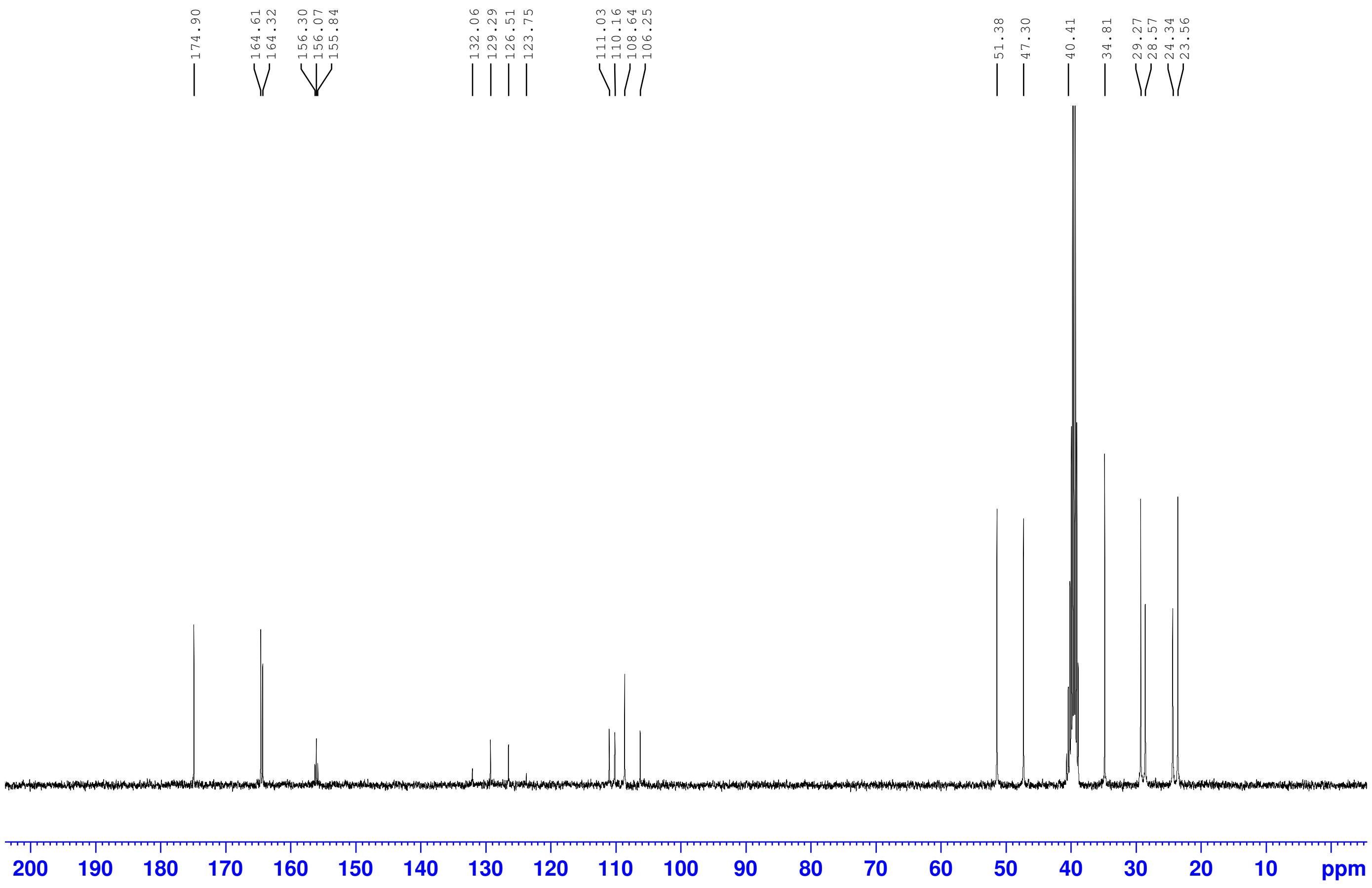

DDD352
F19CPD.DAY DMSO {D:\nmrdata} DDU500 3

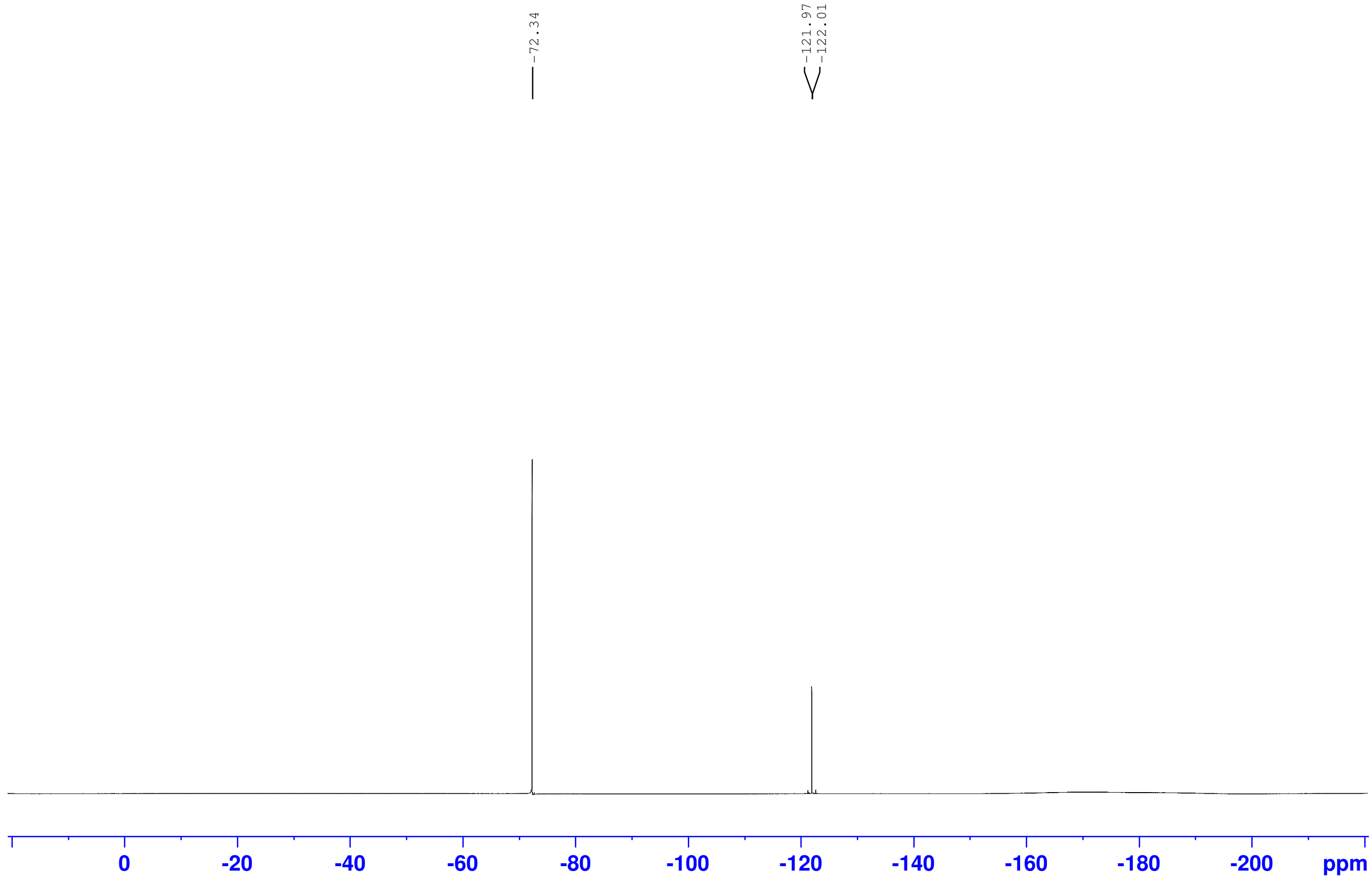

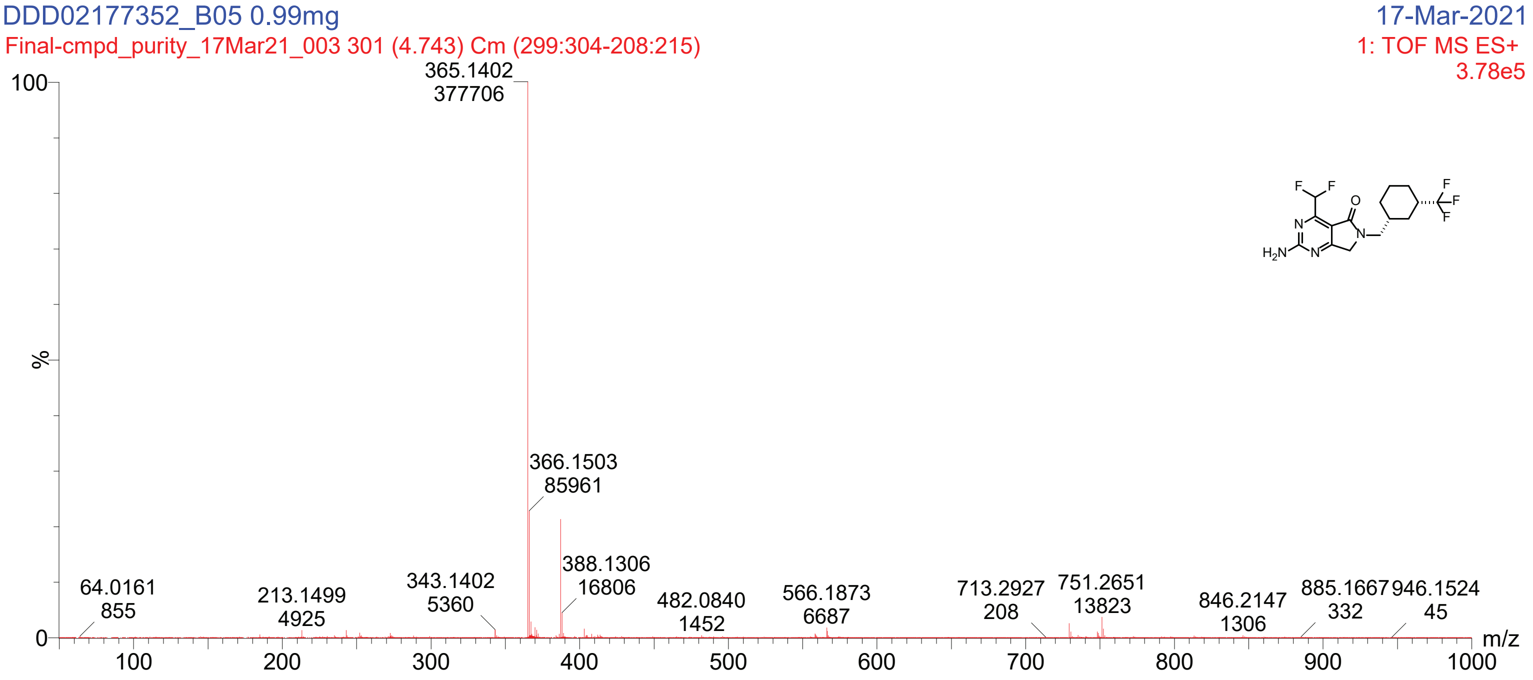

2-amino-4-methyl-6-(((1R,3S)-3-(trifluoromethyl)cyclohexyl)methyl)-6,7-dihydro-5H-pyrrolo[3,4-d] pyrimidin-5-one (DDD478)

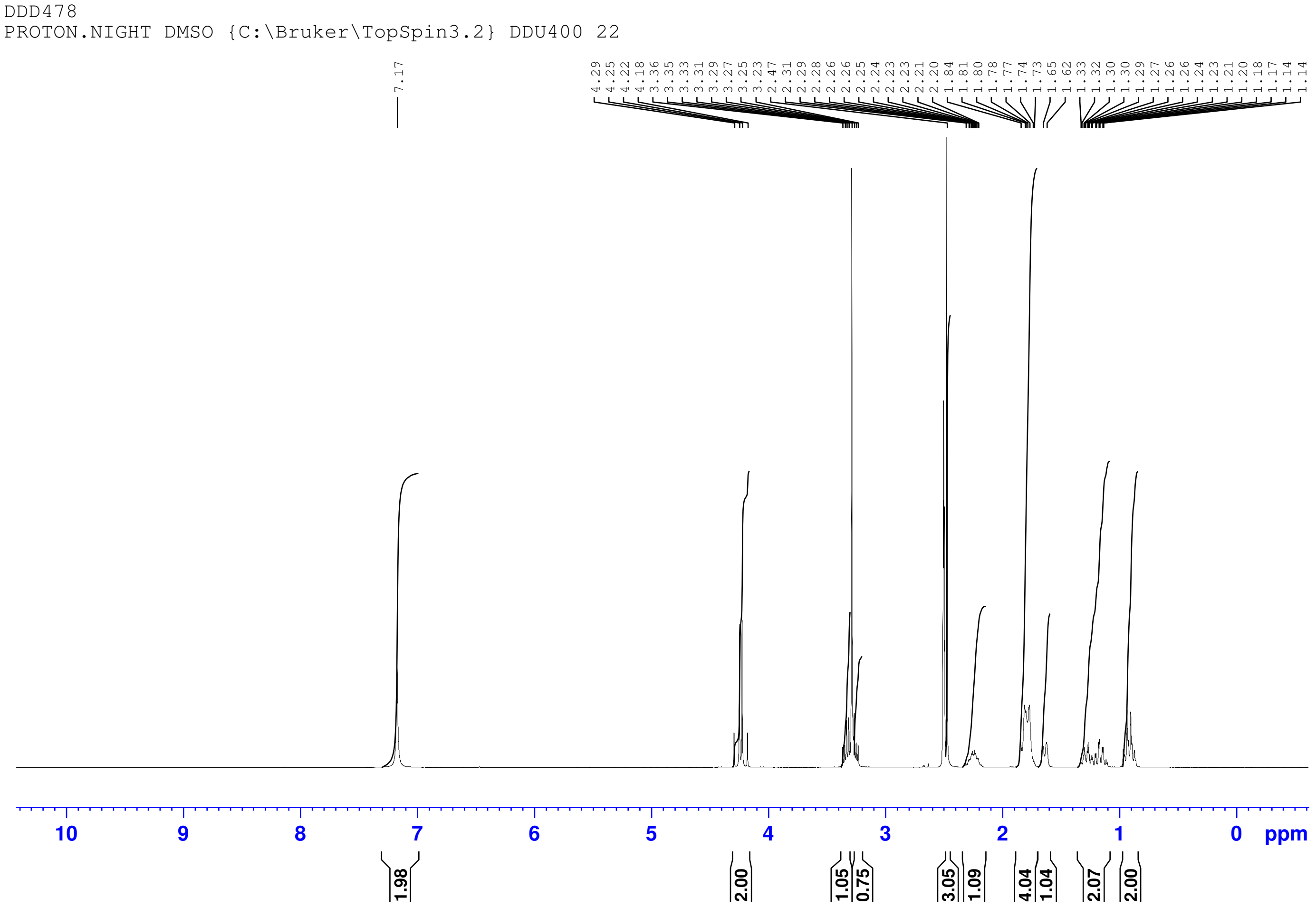

DDD478
CARBON.NIGHT DMSO {C:\Bruker\TopSpin3.2} DDU400 22

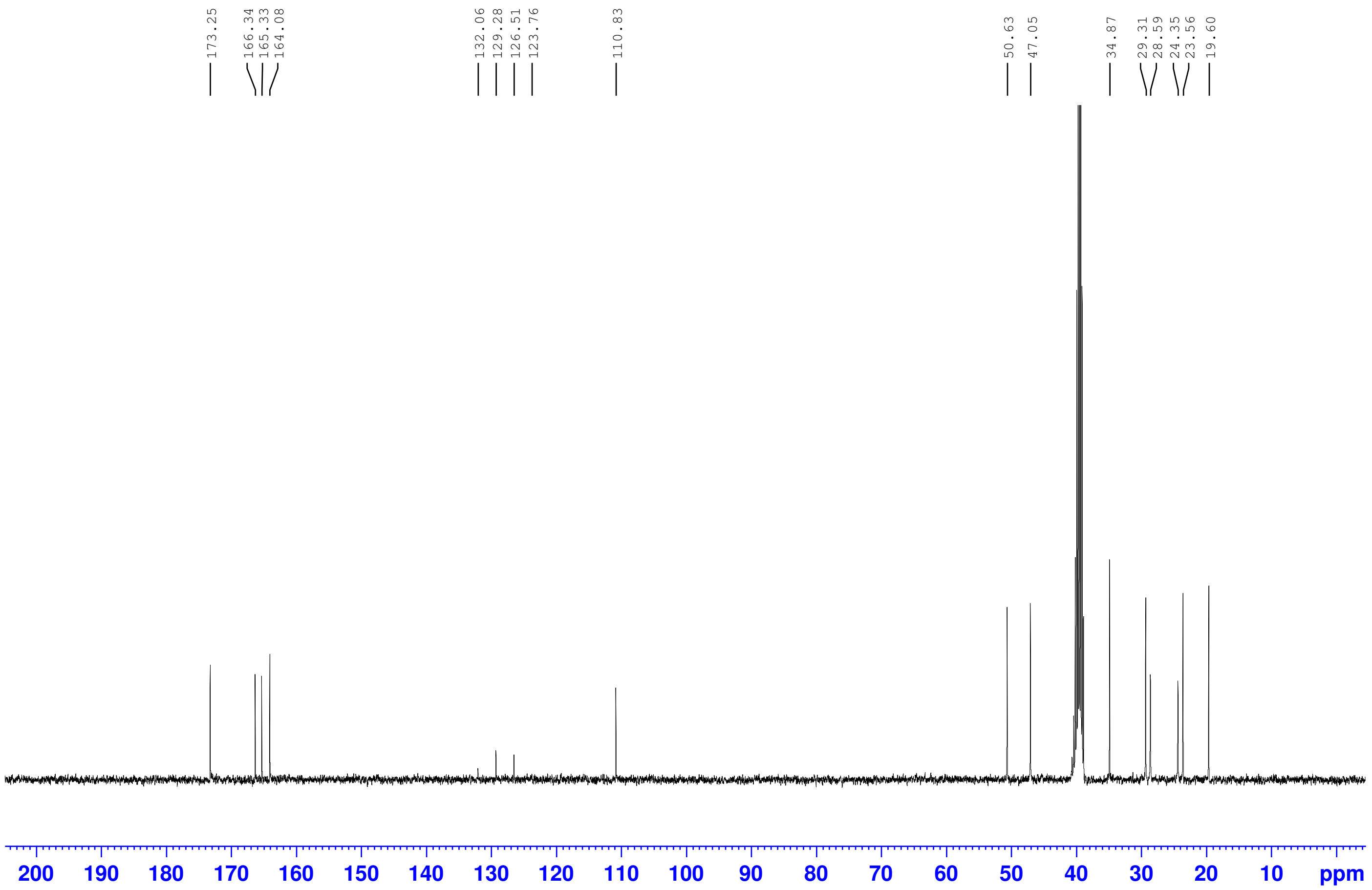

DDD478
F19CPD.DAY DMSO {D:\nmrdata} DDU500 4

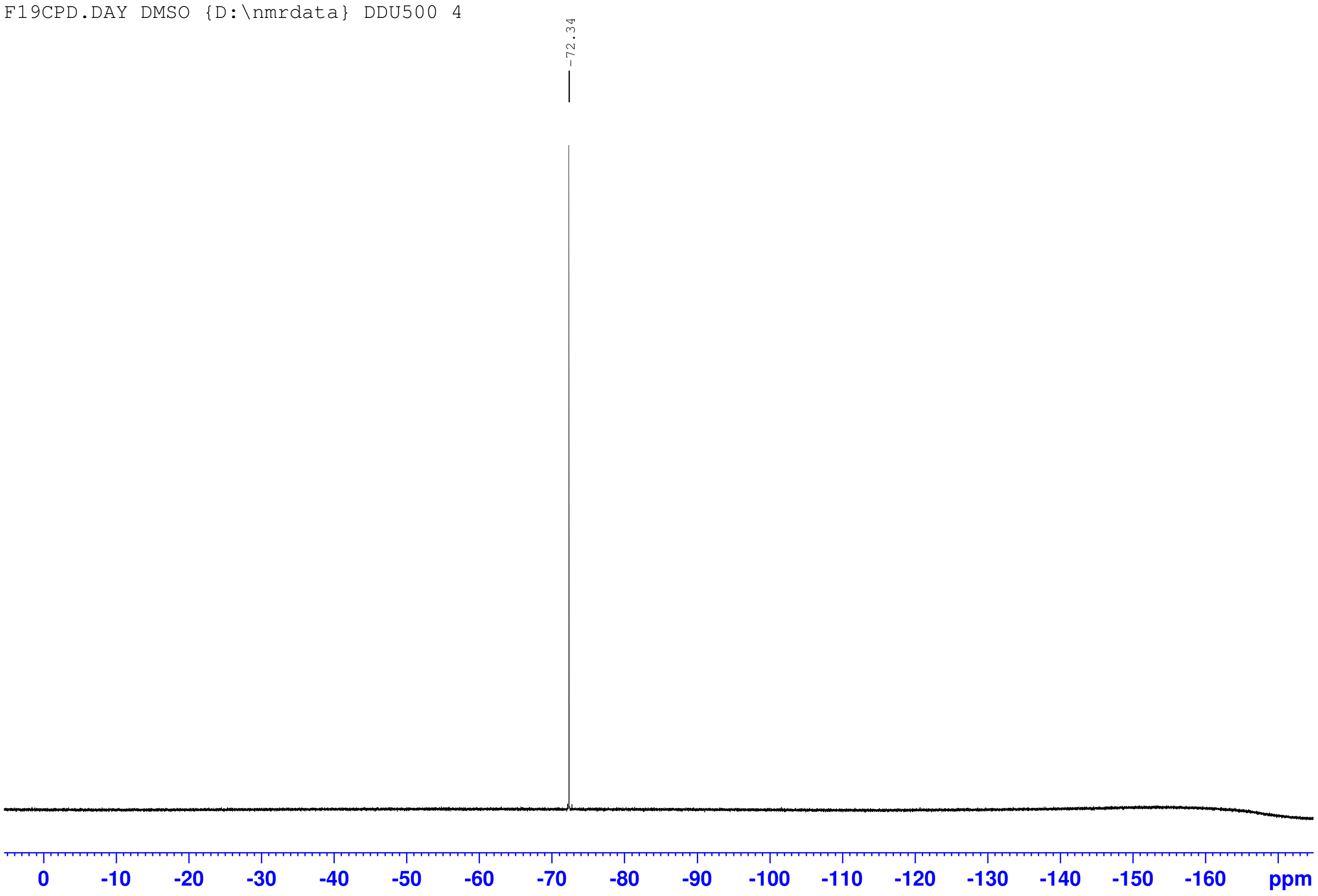

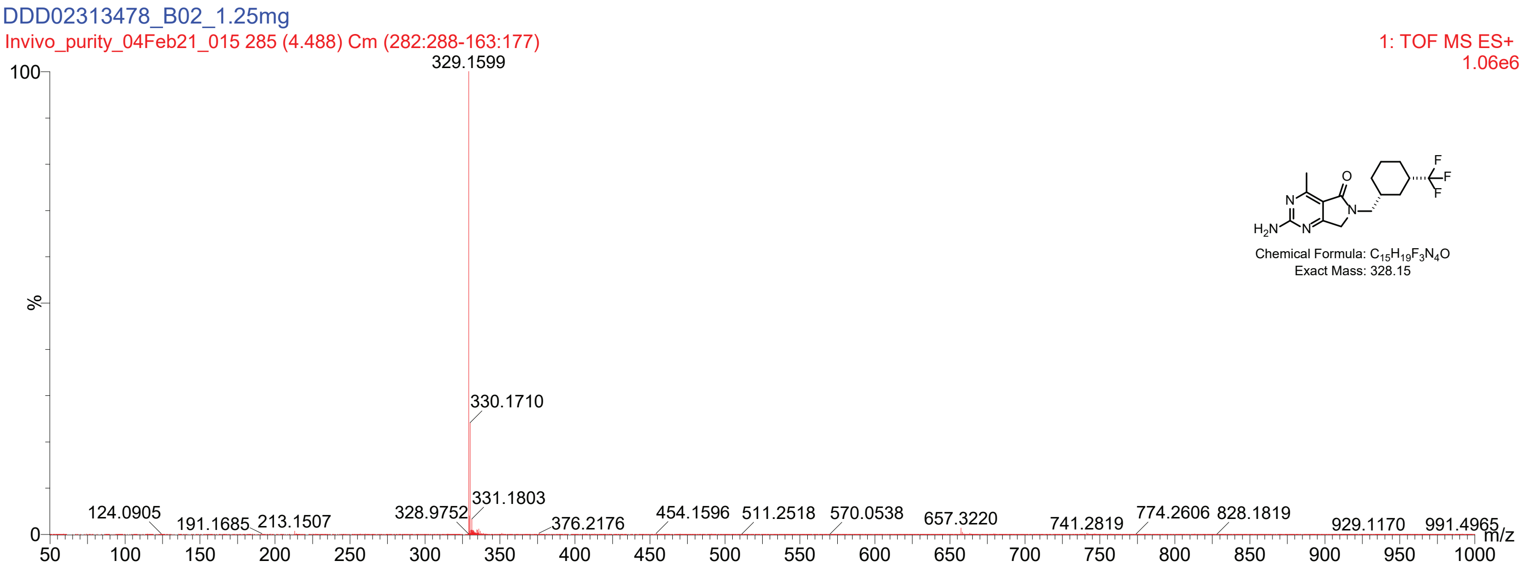

N-((1-hydroxycyclohexyl)methyl)-6-methoxy-4-oxo-4H-chromene-2-carboxamide (DDD583)

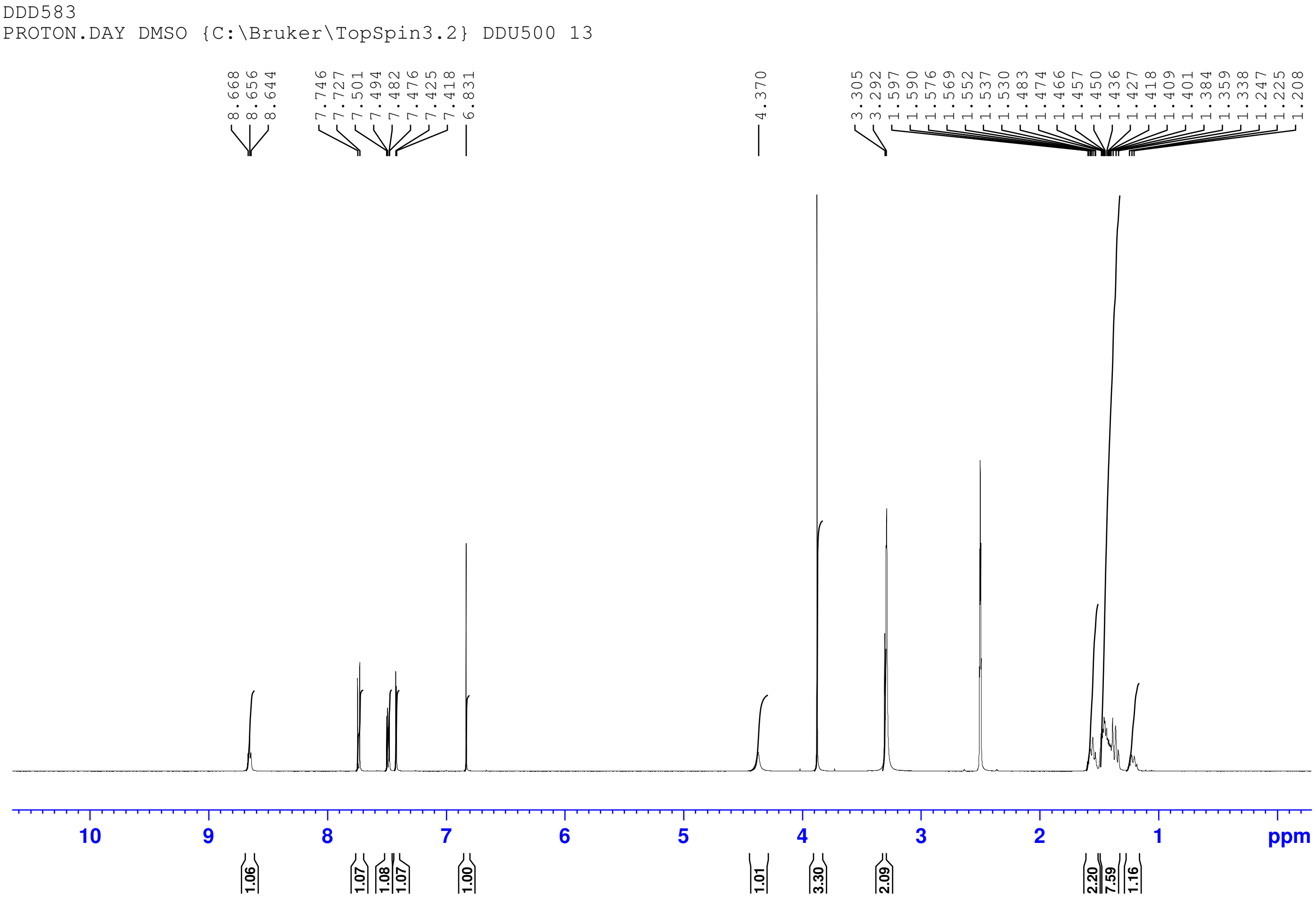

DDD583
CARBON.DAY DMSO {C:\Bruker\TopSpin3.2} DDU500 13

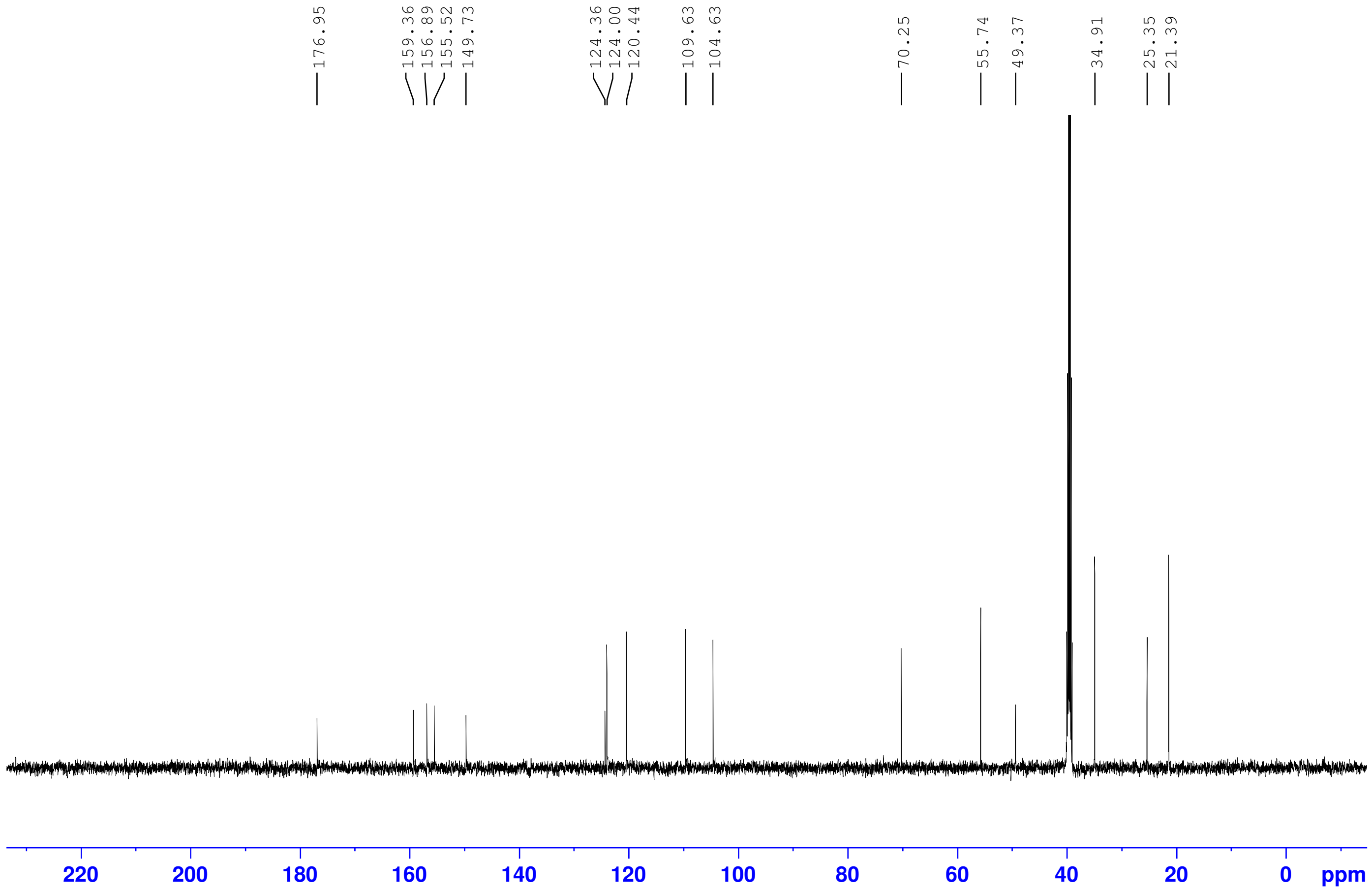

DDD02353583_B02_1.12mg
KW_23July21_006 267 (4.209) Cm (264:270-169:176)

22-Jul-2021
1: TOF MS ES+
3.17e5

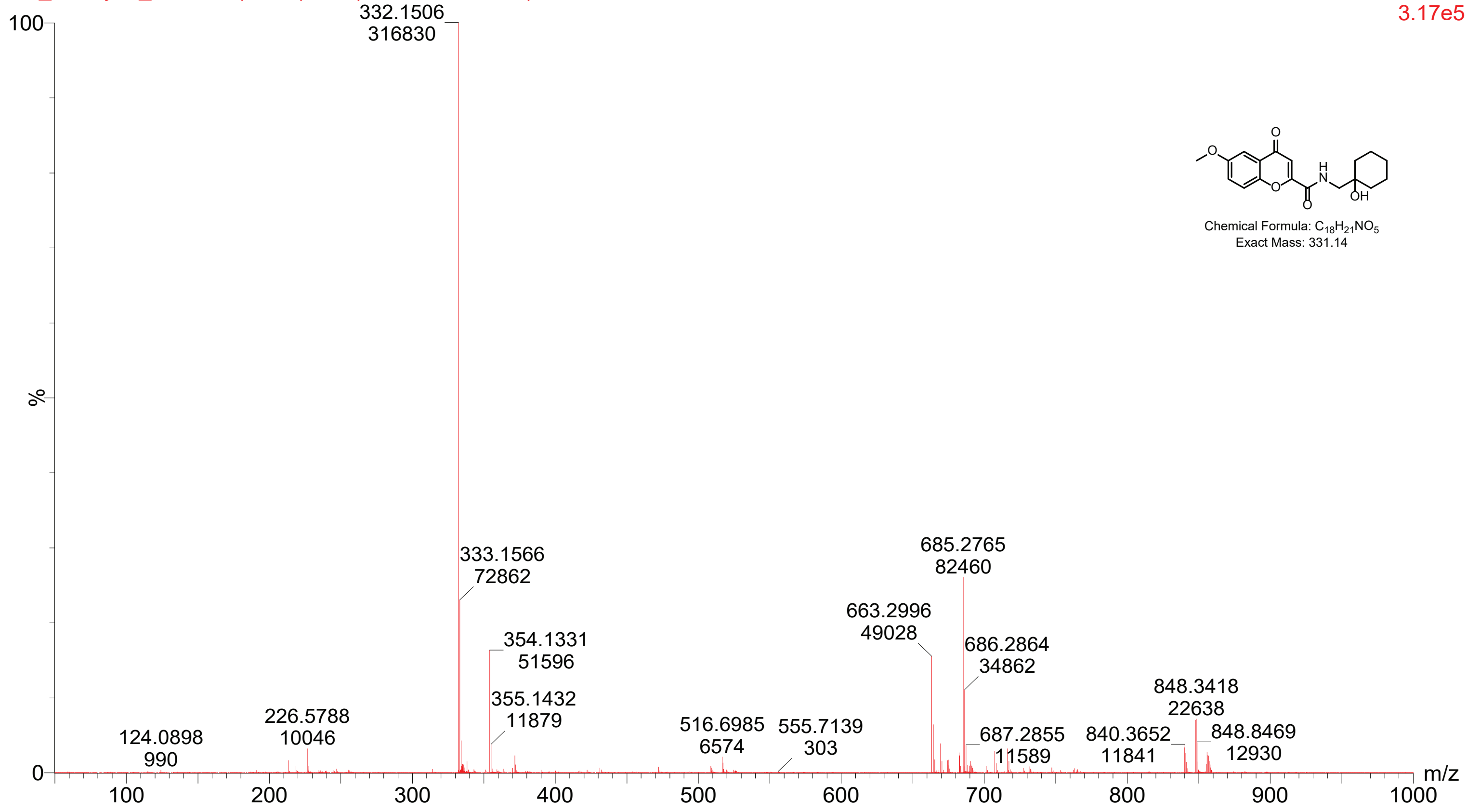

Chemical Formula: $C_{18}H_{21}NO_5$
Exact Mass: 331.14

6-hydroxy-N-((1-hydroxycyclohexyl)methyl)-4-oxo-4H-chromene-2-carboxamide (DDD508)

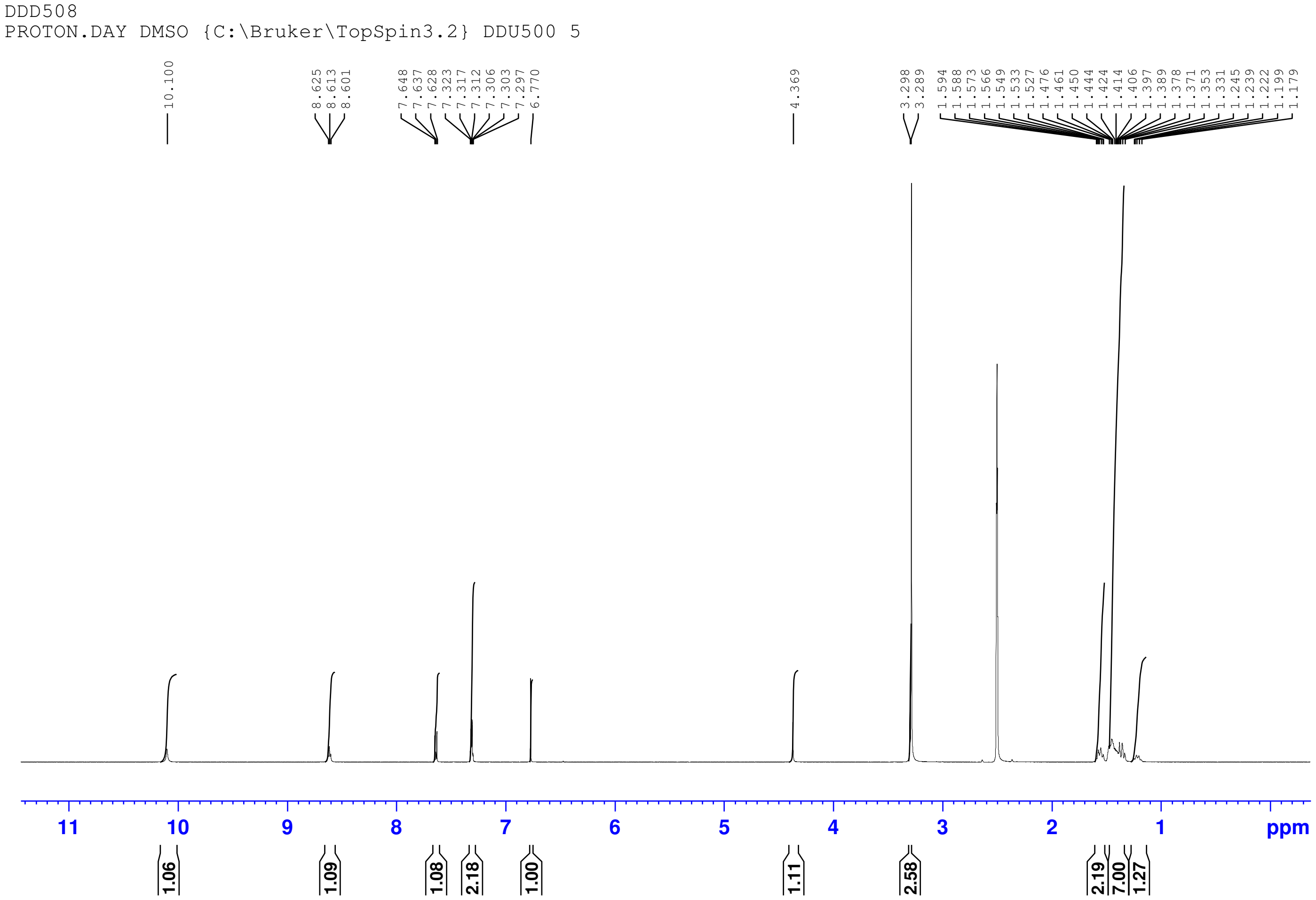

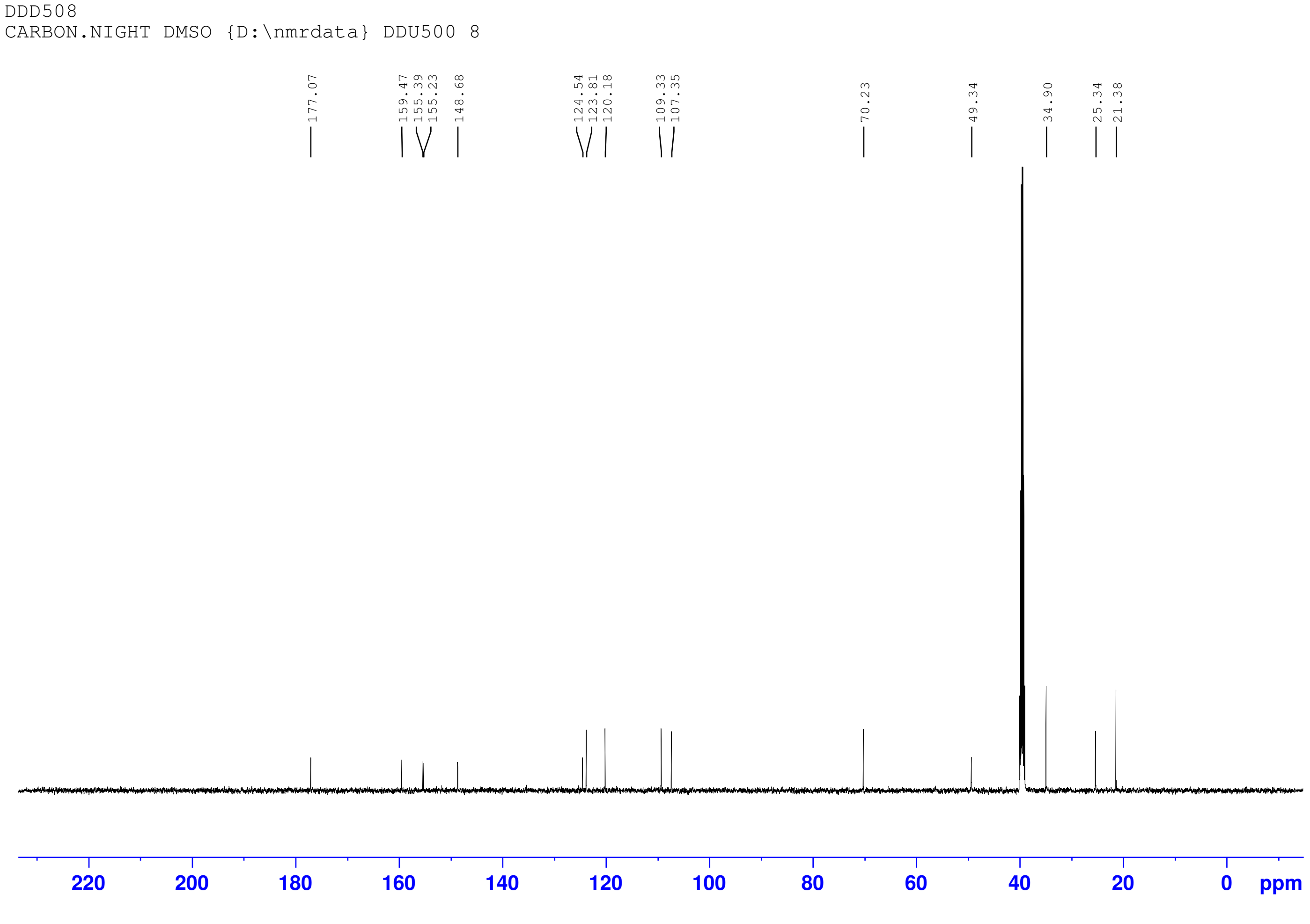

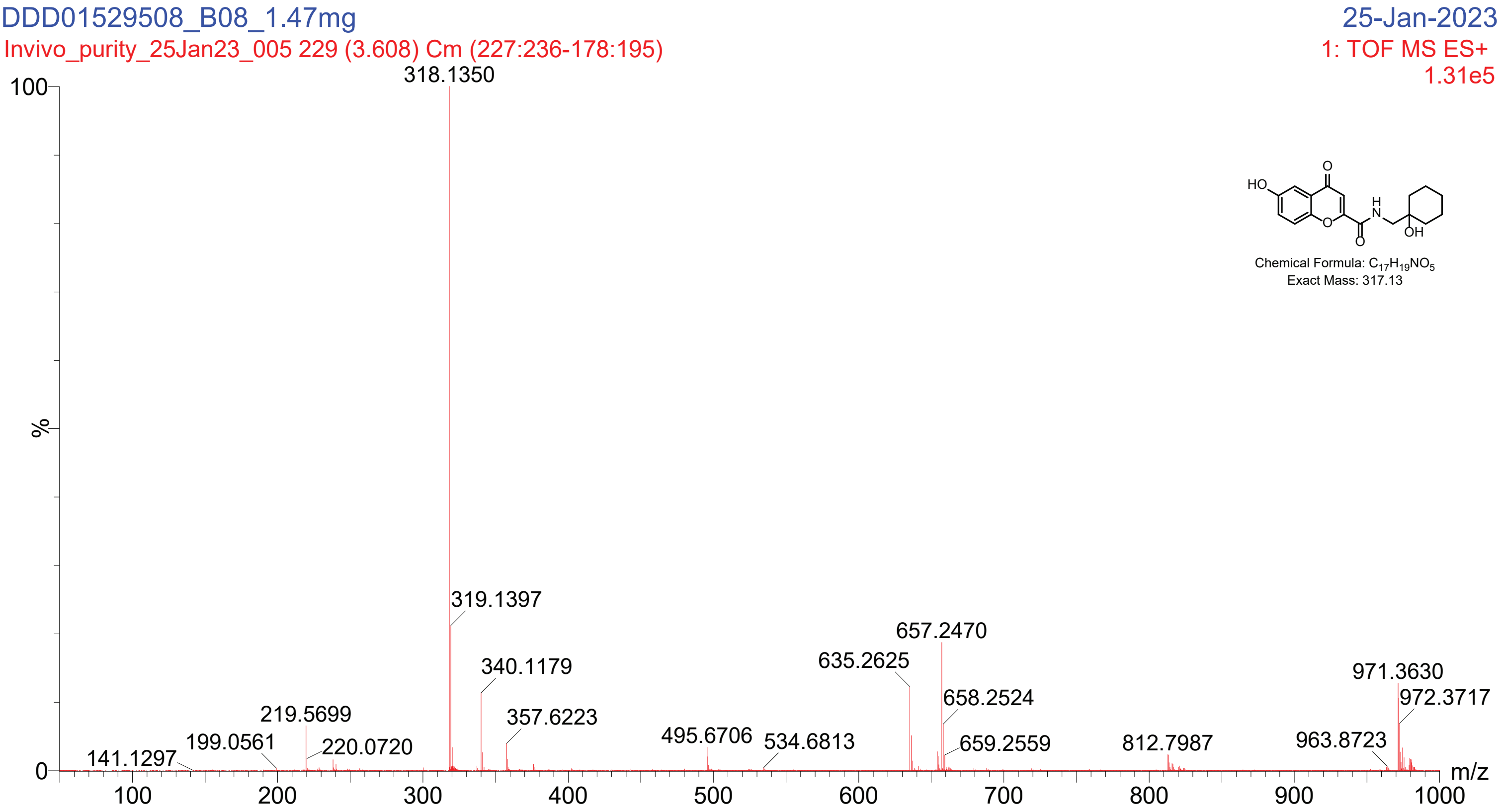

7-Fluoro-8-hydroxy-4-oxo-N-(tetrahydropyran-2-ylmethyl)chromene-2-carboxamide (DDD695)

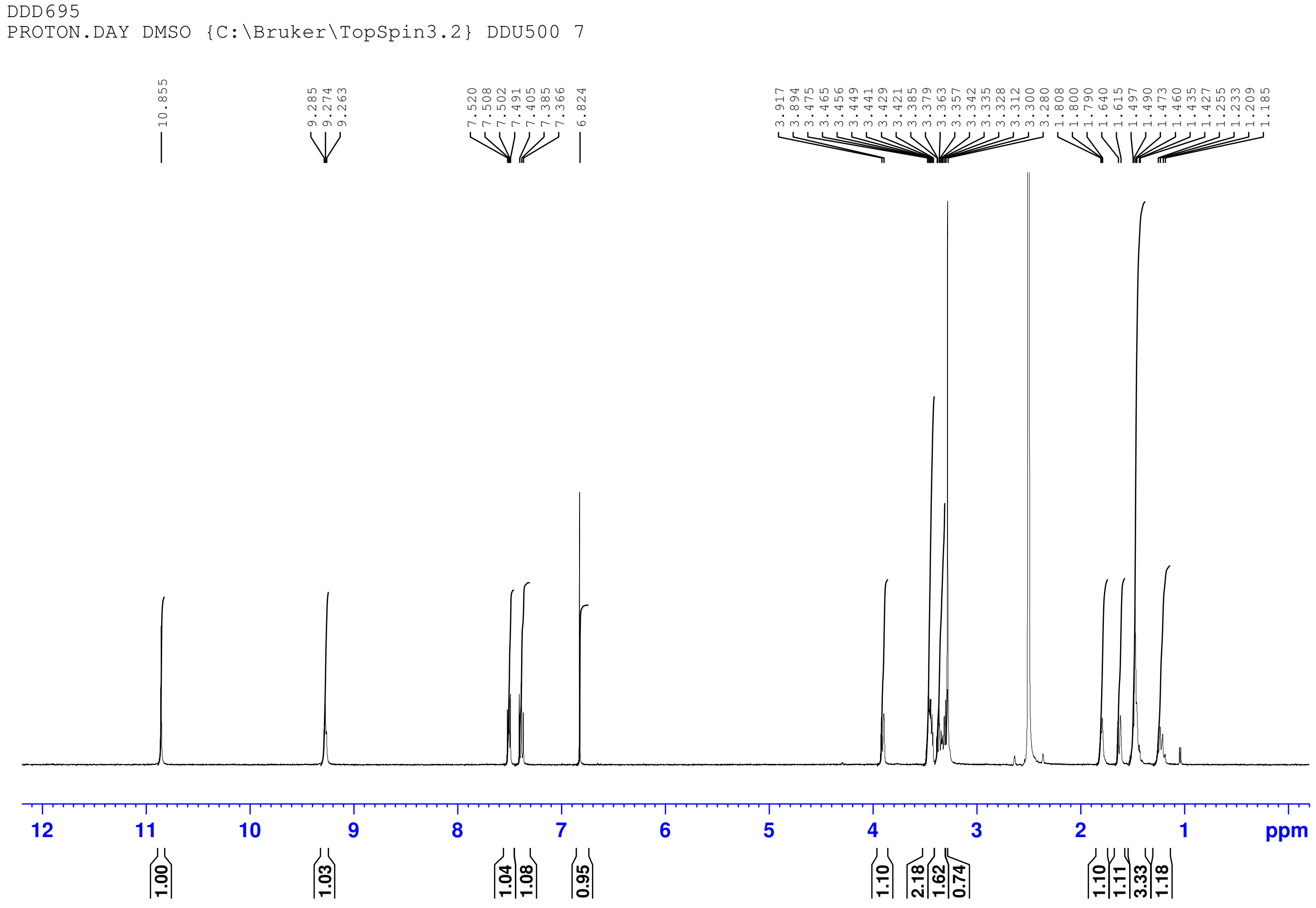

DDD695
CARBON.DAY DMSO {C:\Bruker\TopSpin3.2} DDU500 16

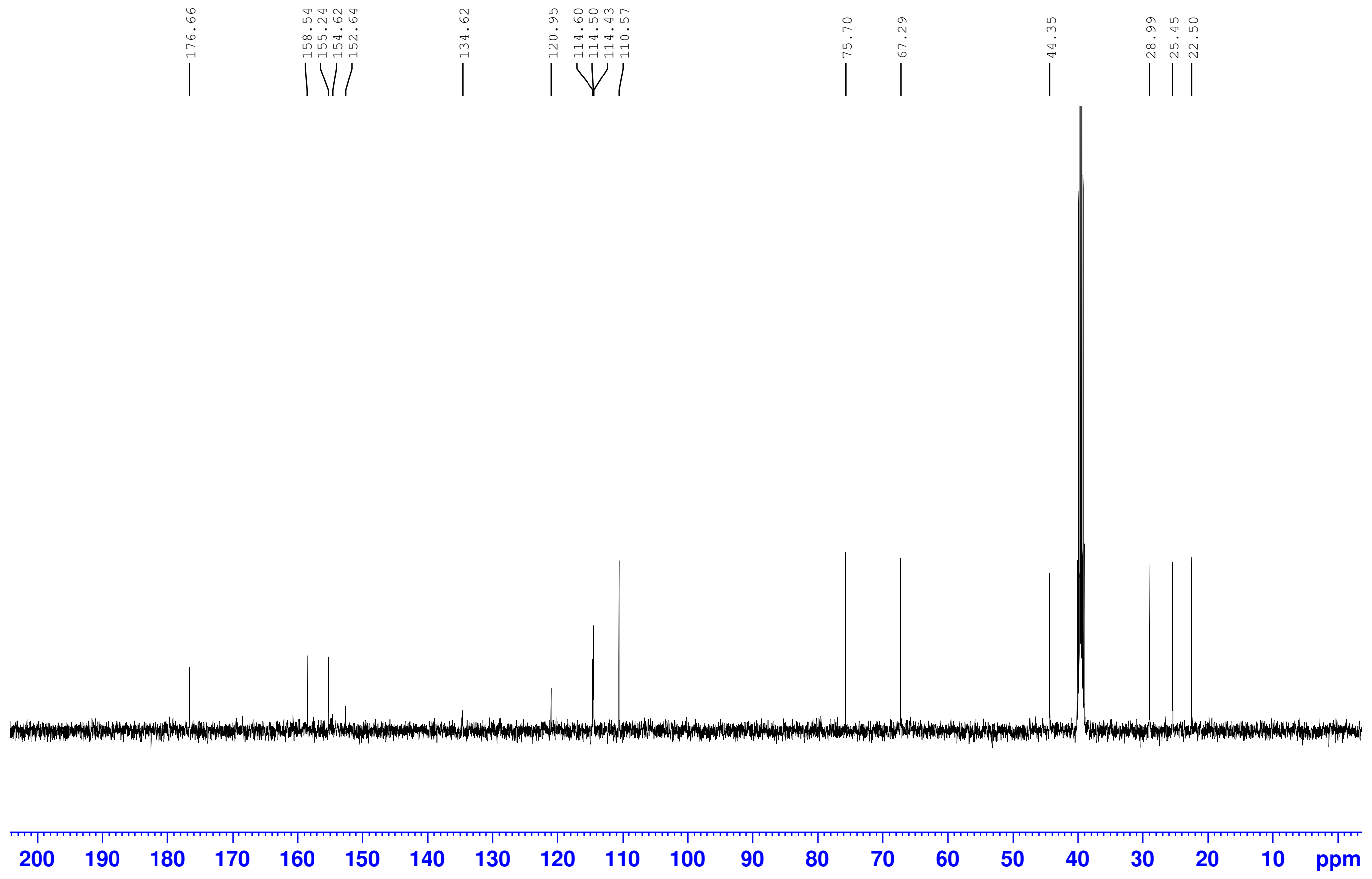

DDD695
F19CPD.DAY DMSO {D:\nmrdata} DDU500 6

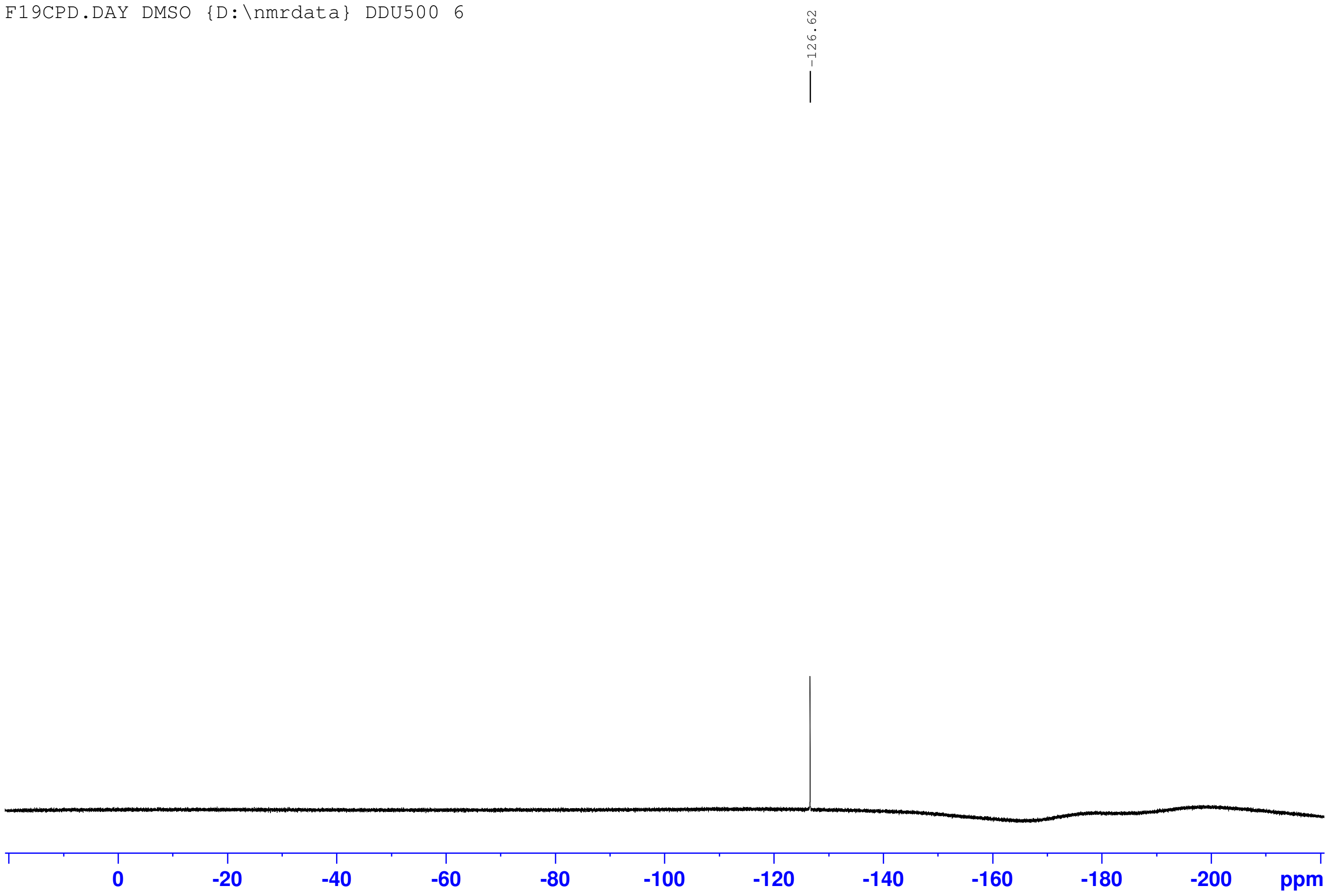

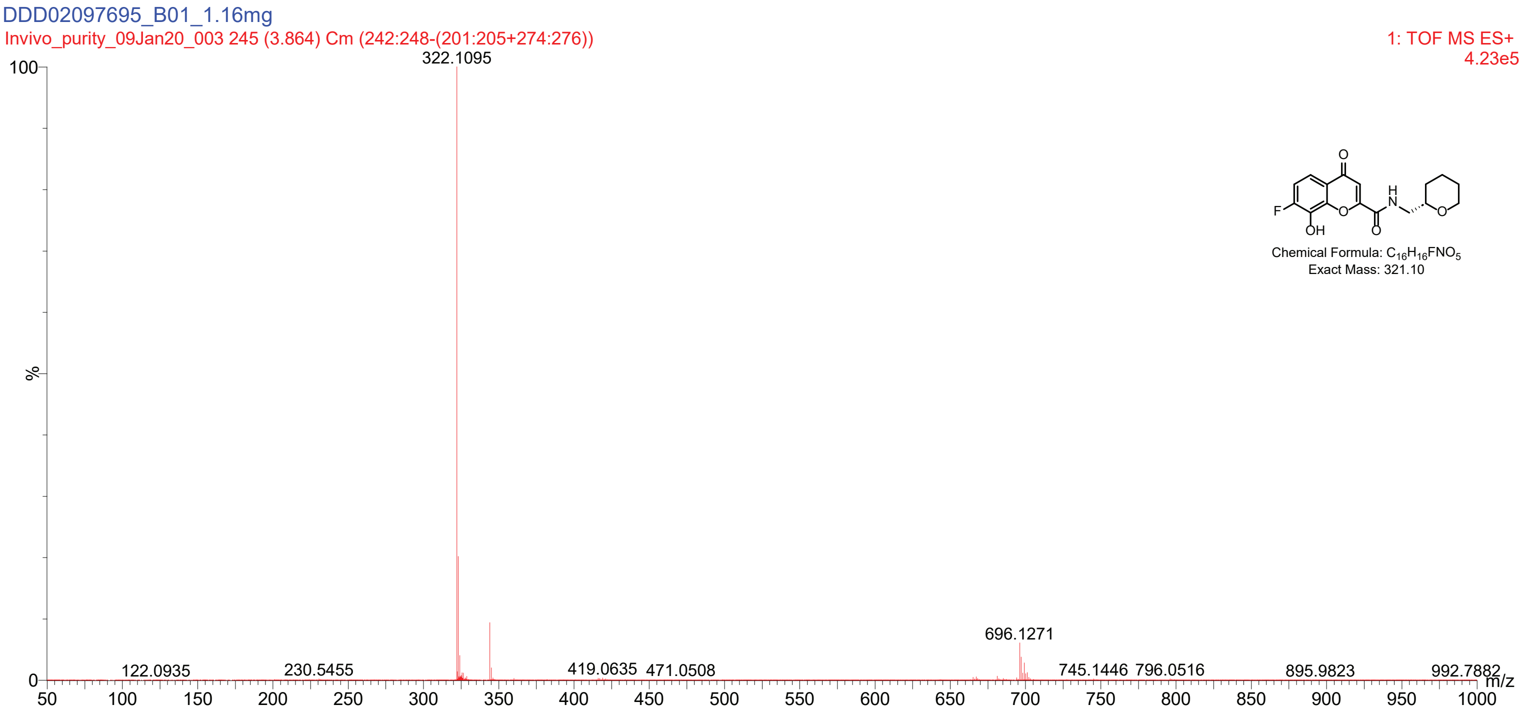

(S)-N-(1-cyclohexylethyl)-6-methoxy-4-oxo-8-((2-(pyrrolidin-1-yl)ethyl)sulfonamido)-4H-chromene-2-carboxamide (DDD582)

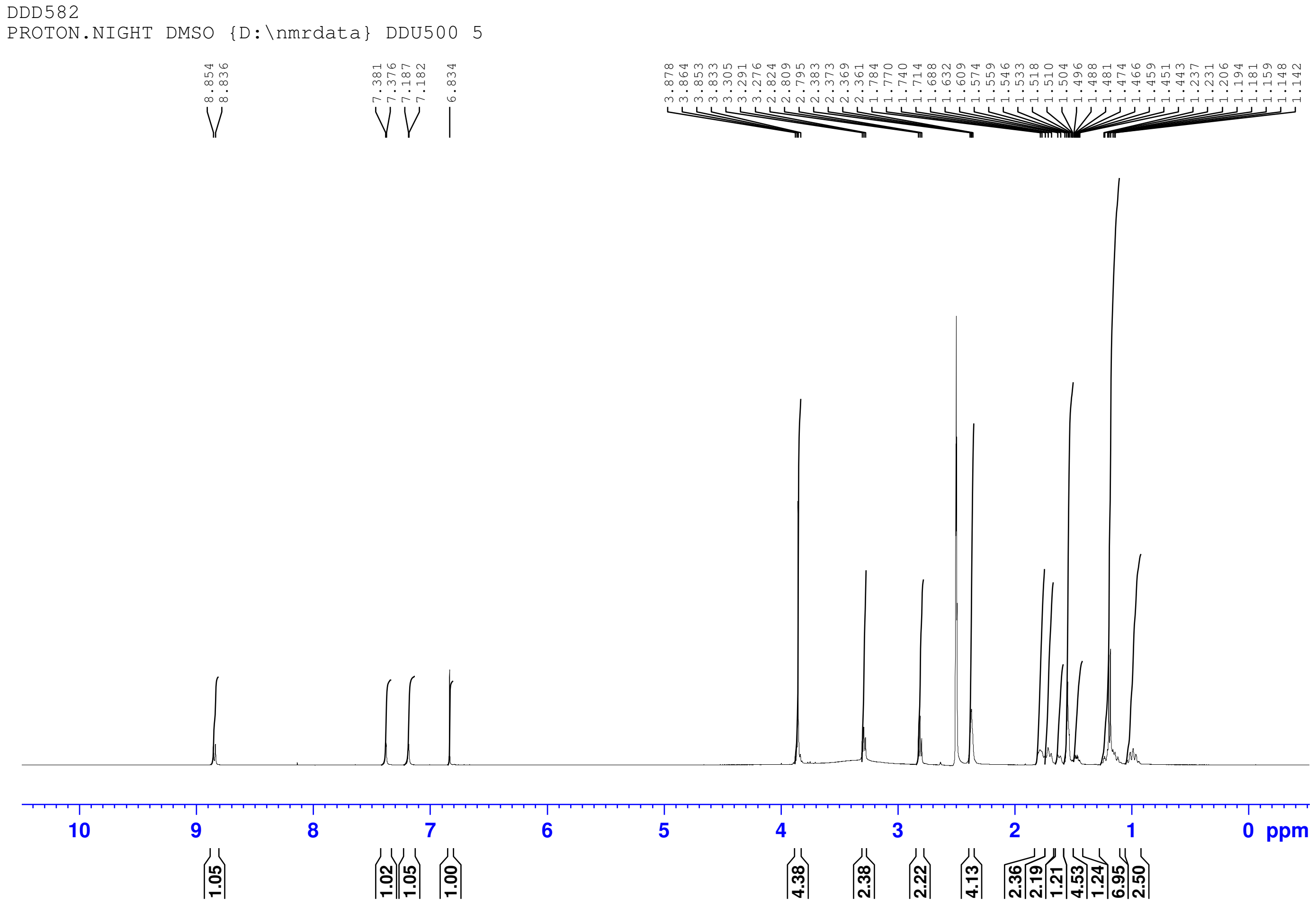

DDD582
CARBON.NIGHT DMSO {D:\nmrdata} DDU500 2

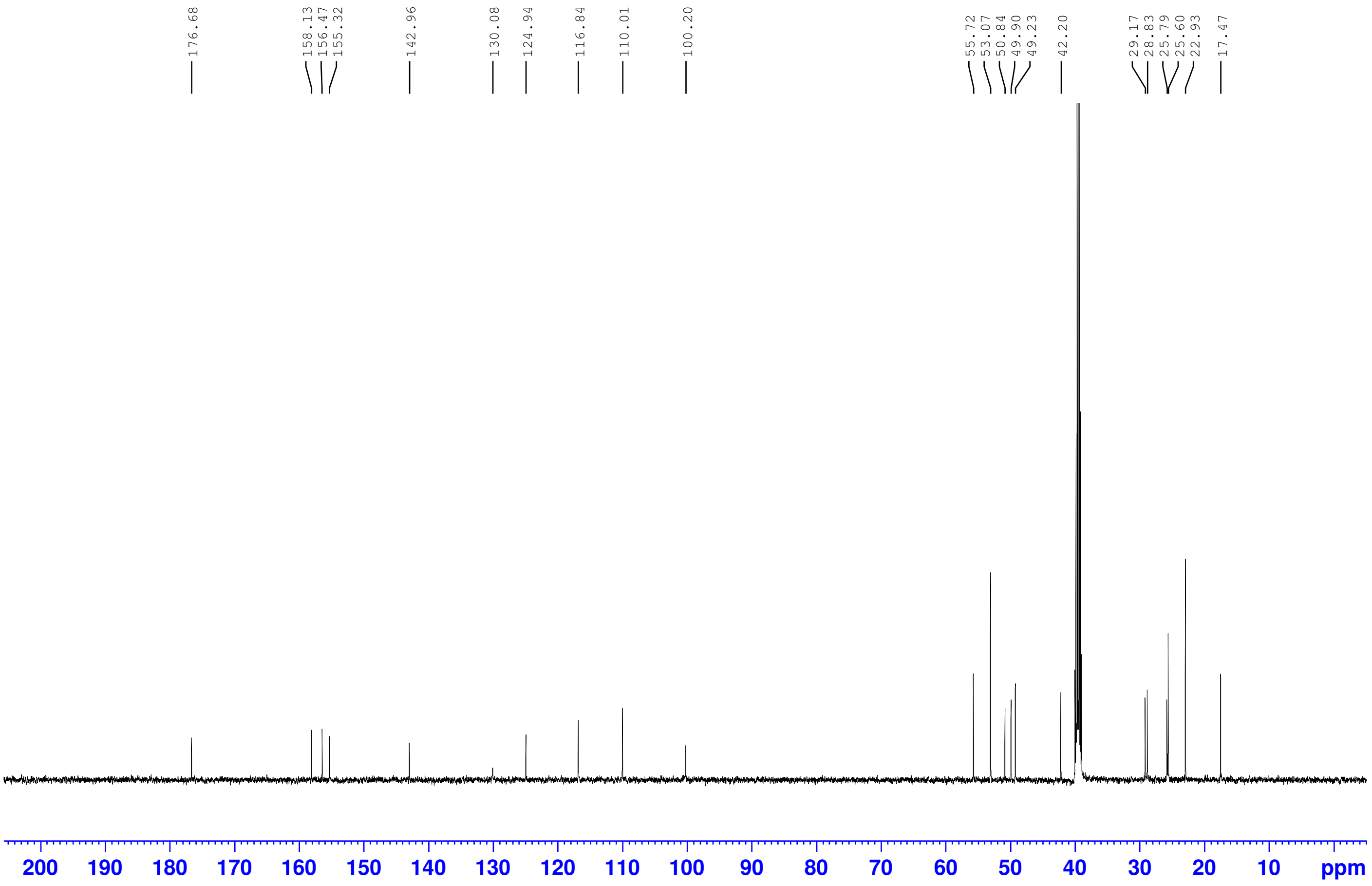

DDD02378582_B02_1.09mg
KW_16July21_018 263 (4.141) Cm (259:267-(198+159:165))

18-Jul-2021
1: TOF MS ES+
9.65e5

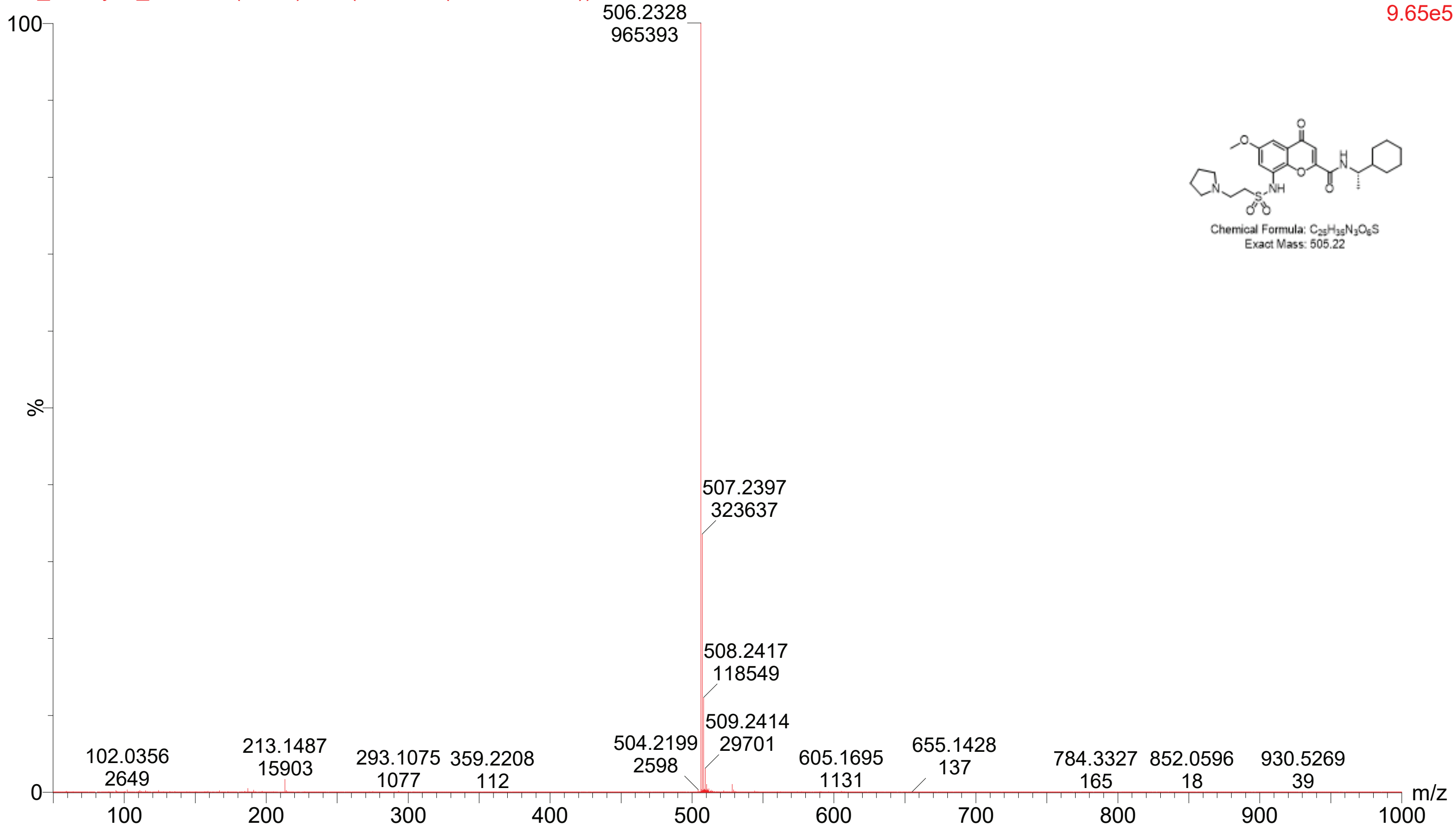

7-fluoro-8-hydroxy-N-((1-hydroxycyclohexyl)methyl)-4-oxo-4H-chromene-2-carboxamide (DDD844)

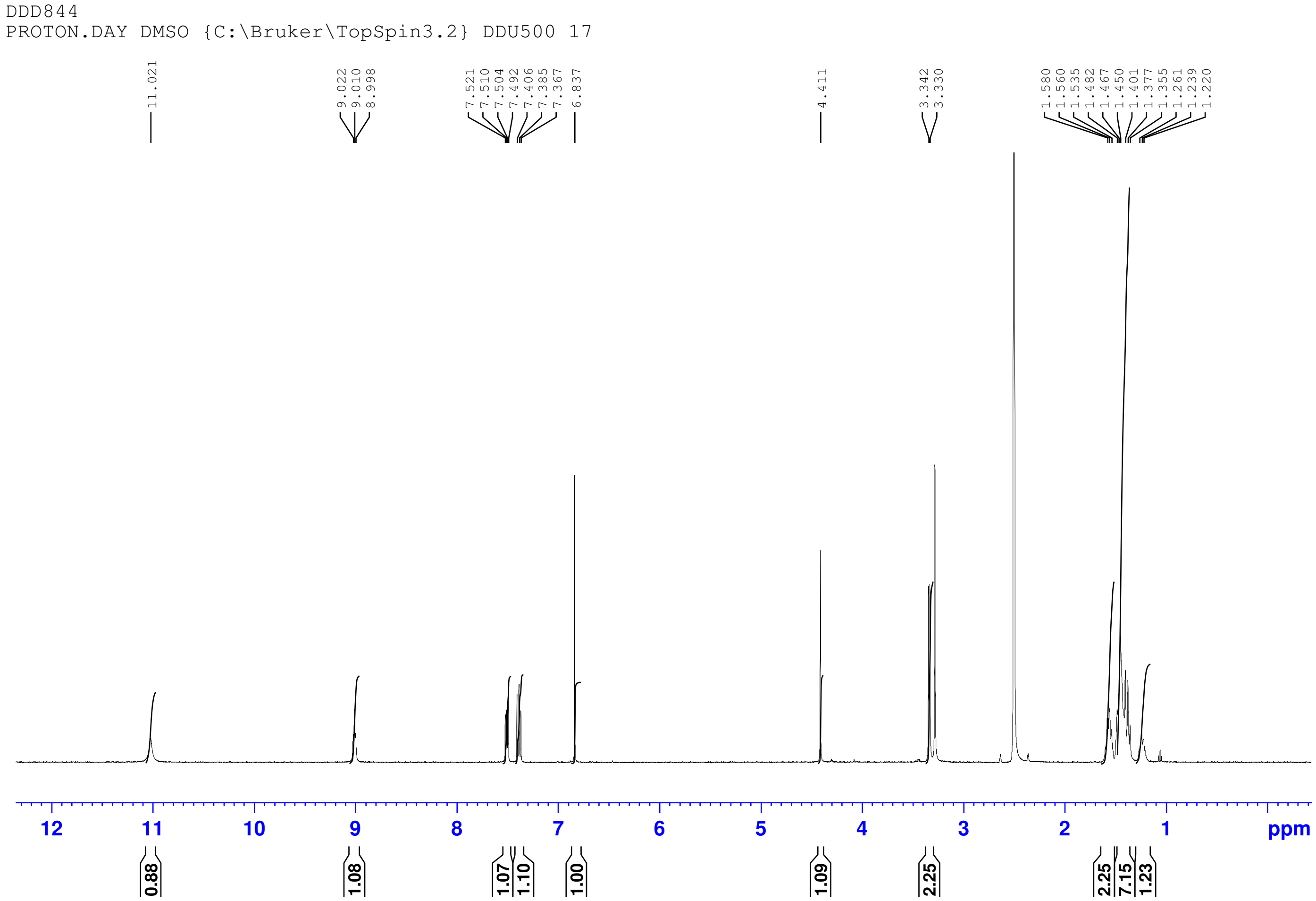

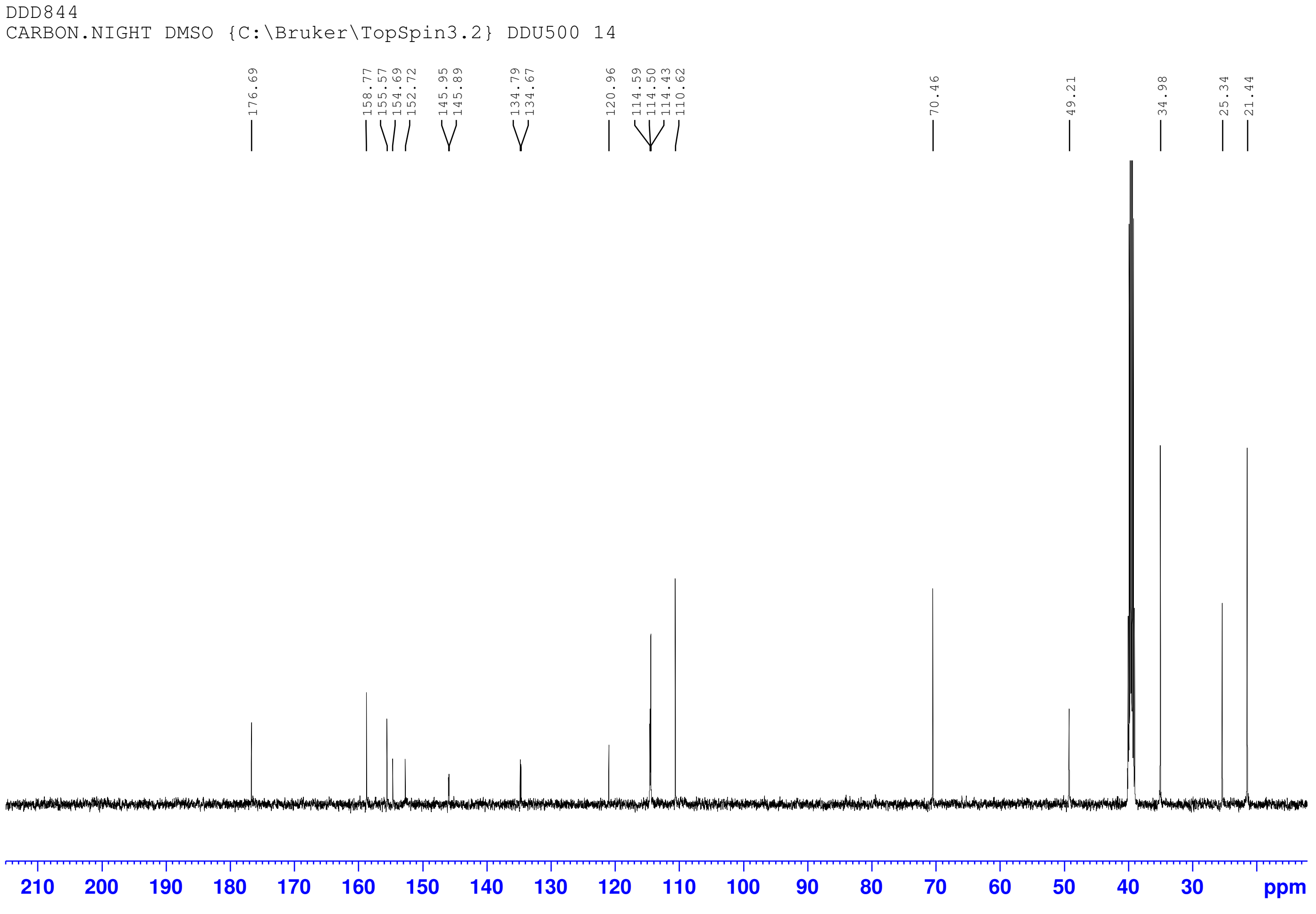

DDD844
F19.DAY DMSO {C:\Bruker\TopSpin3.2} DDU500 14

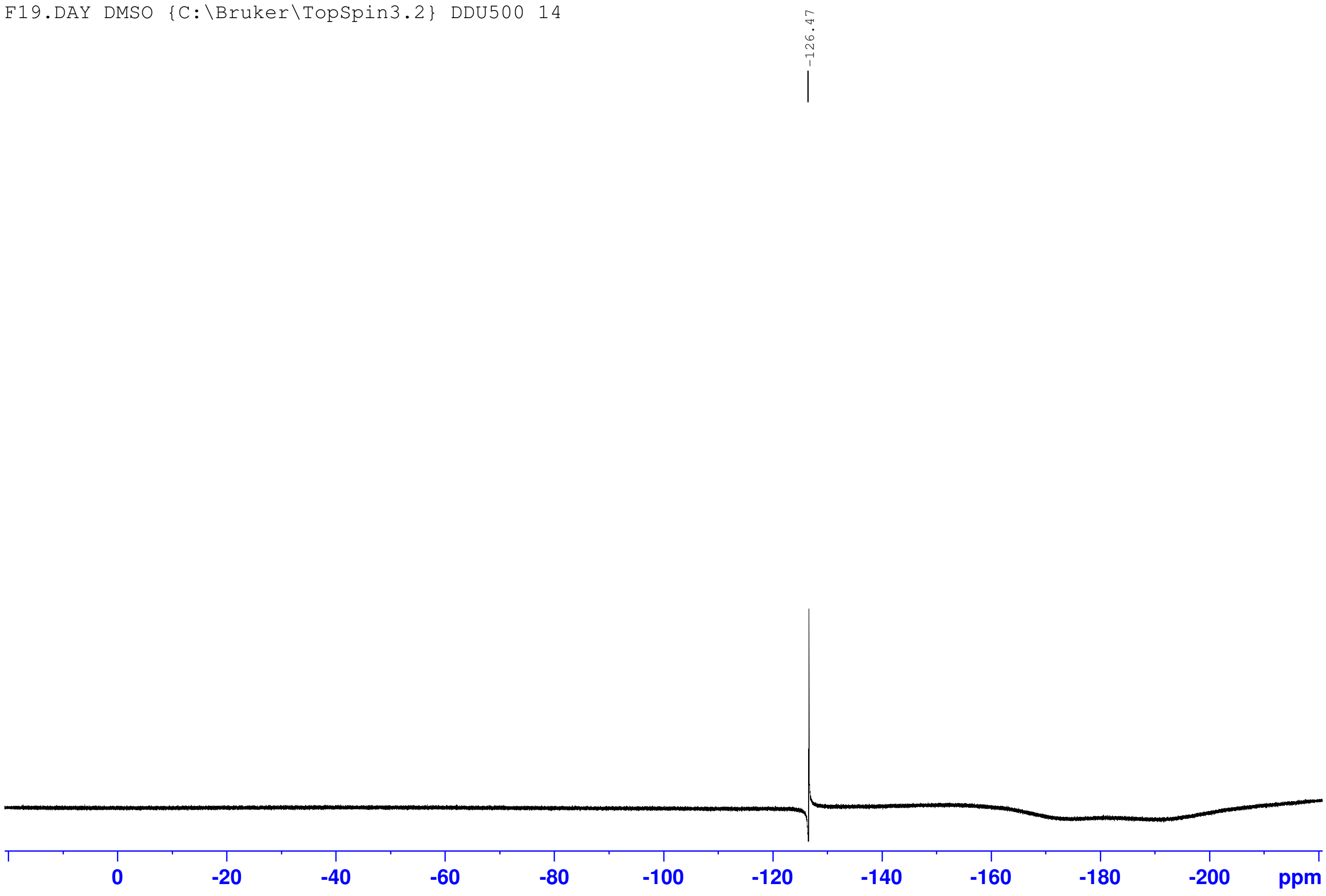

DDD01538844_B05_1.01mg
Purity_24Oct19_011 251 (3.953) Cm (247:254-(155:197+286:310))

1: TOF MS ES+
7.48e4

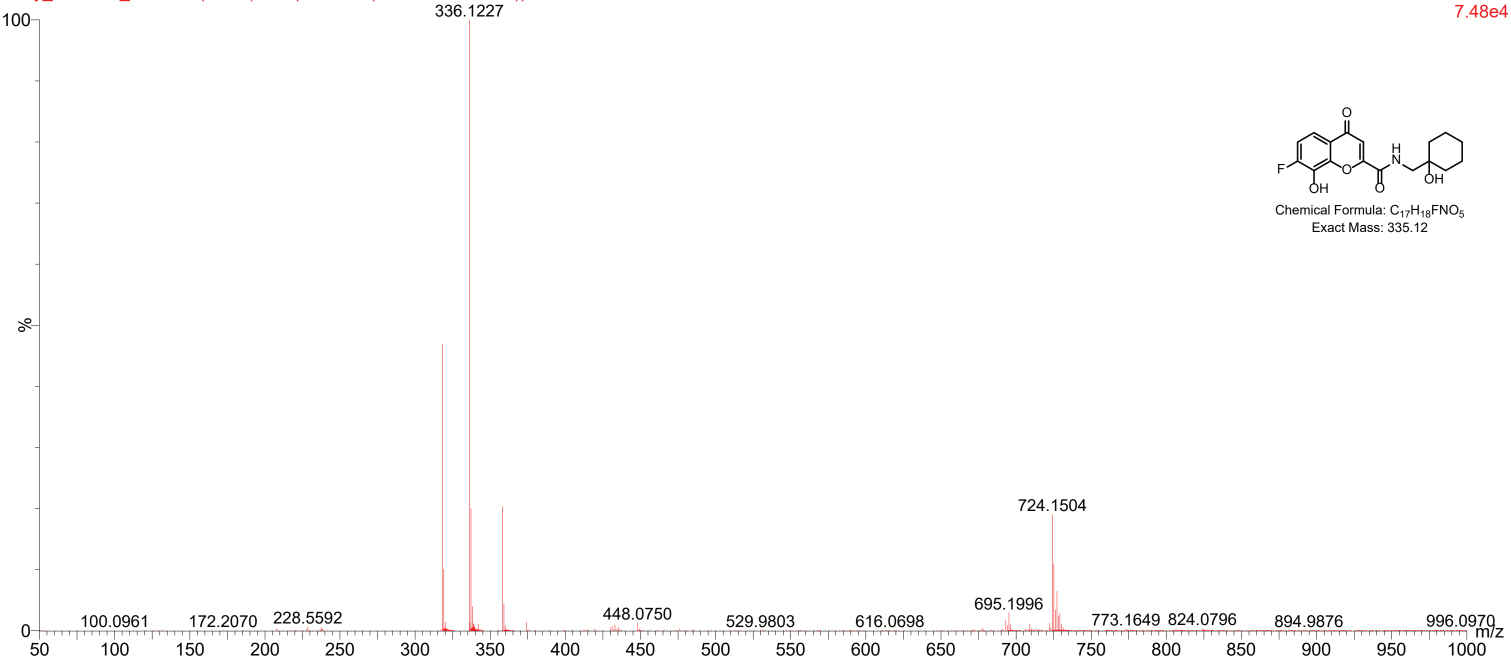

8-amino-6-fluoro-N-((1-hydroxycyclohexyl)methyl)-4-oxo-4H-chromene-2-carboxamide (DDD229)

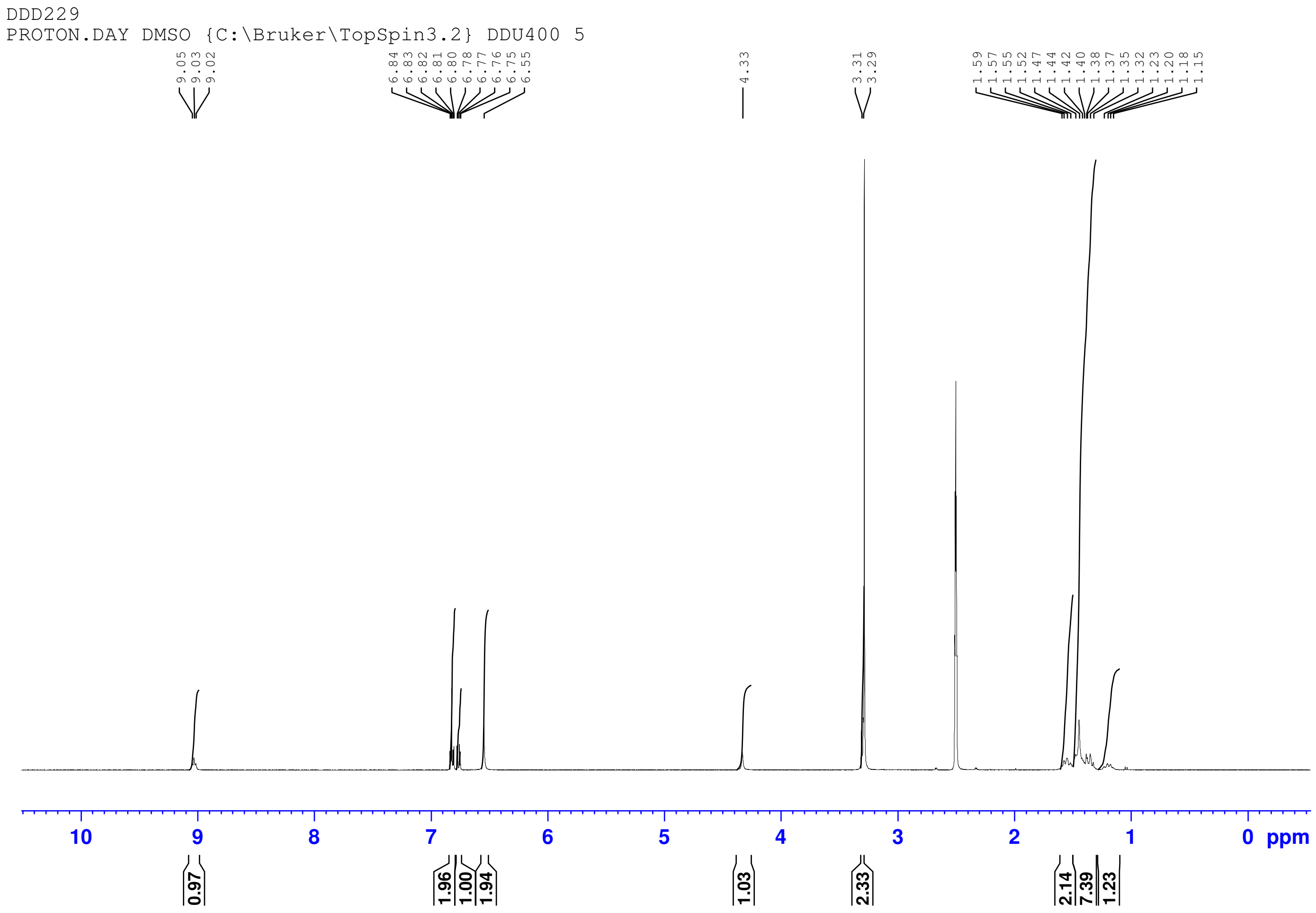

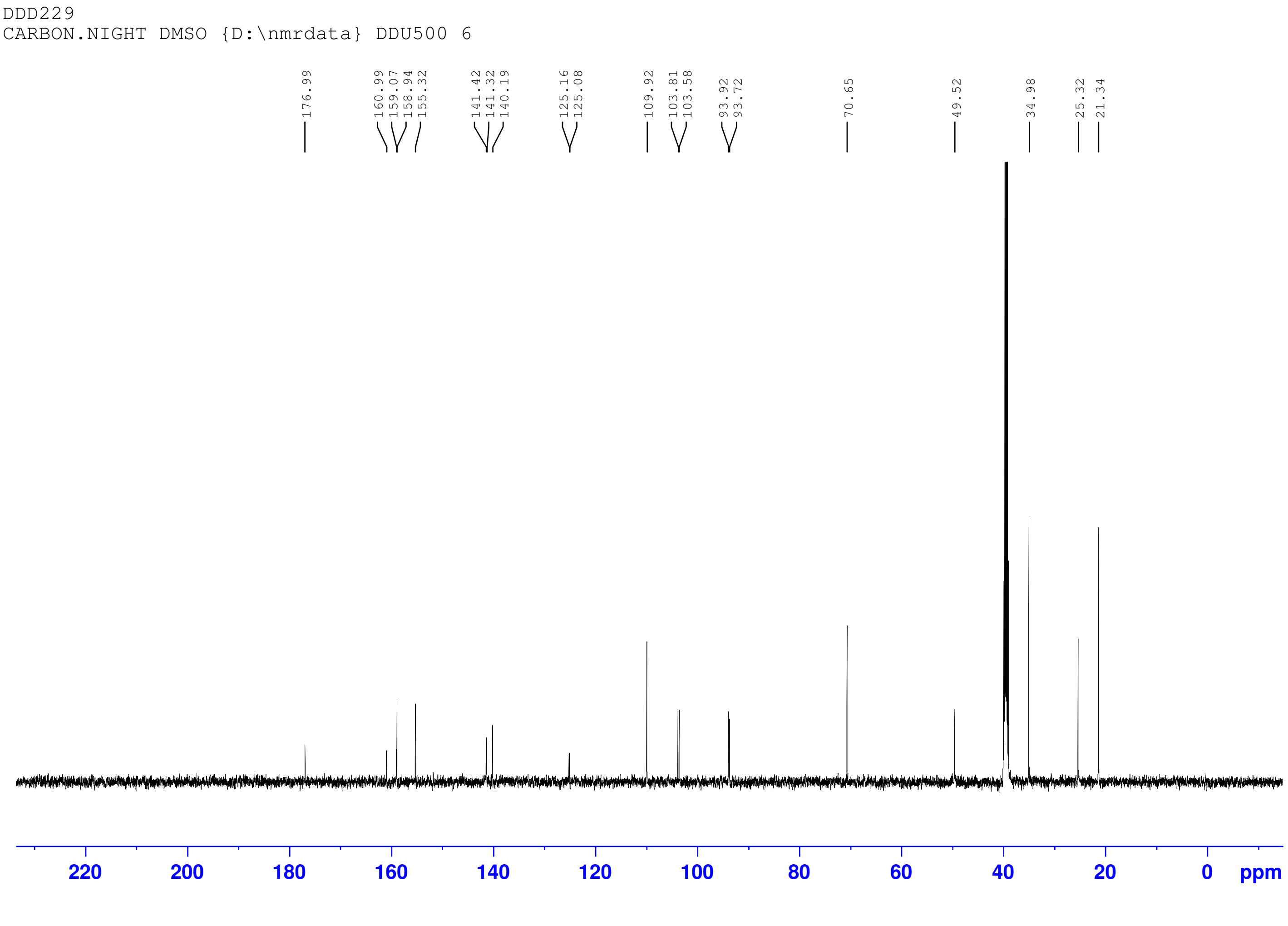

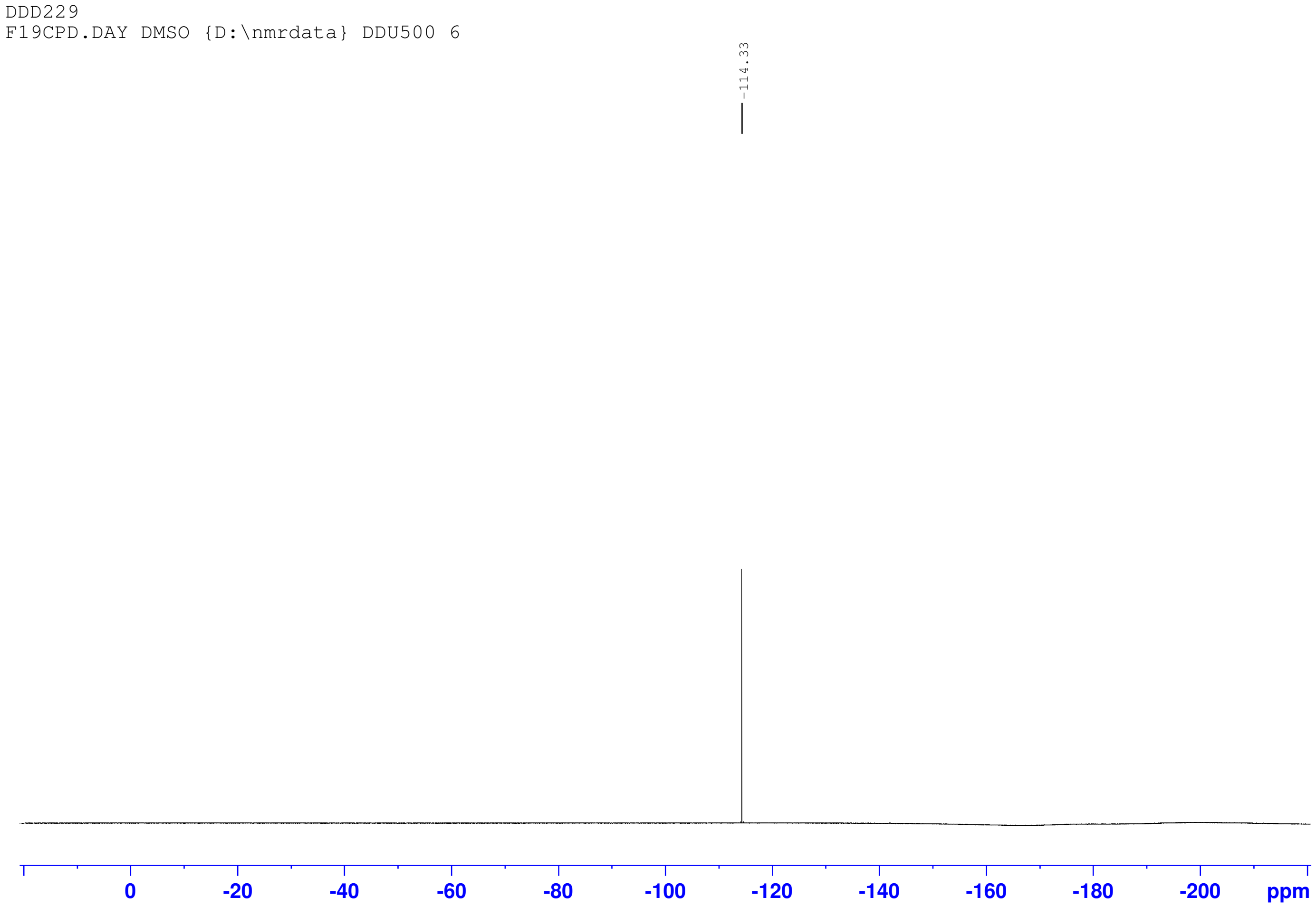
DDD229
F19CPD.DAY DMSO {D:\nmrdata} DDU500 6
-114.33
0
-20
-40
-60
-80
-100
-120
-140
-160
-180
-200
ppm

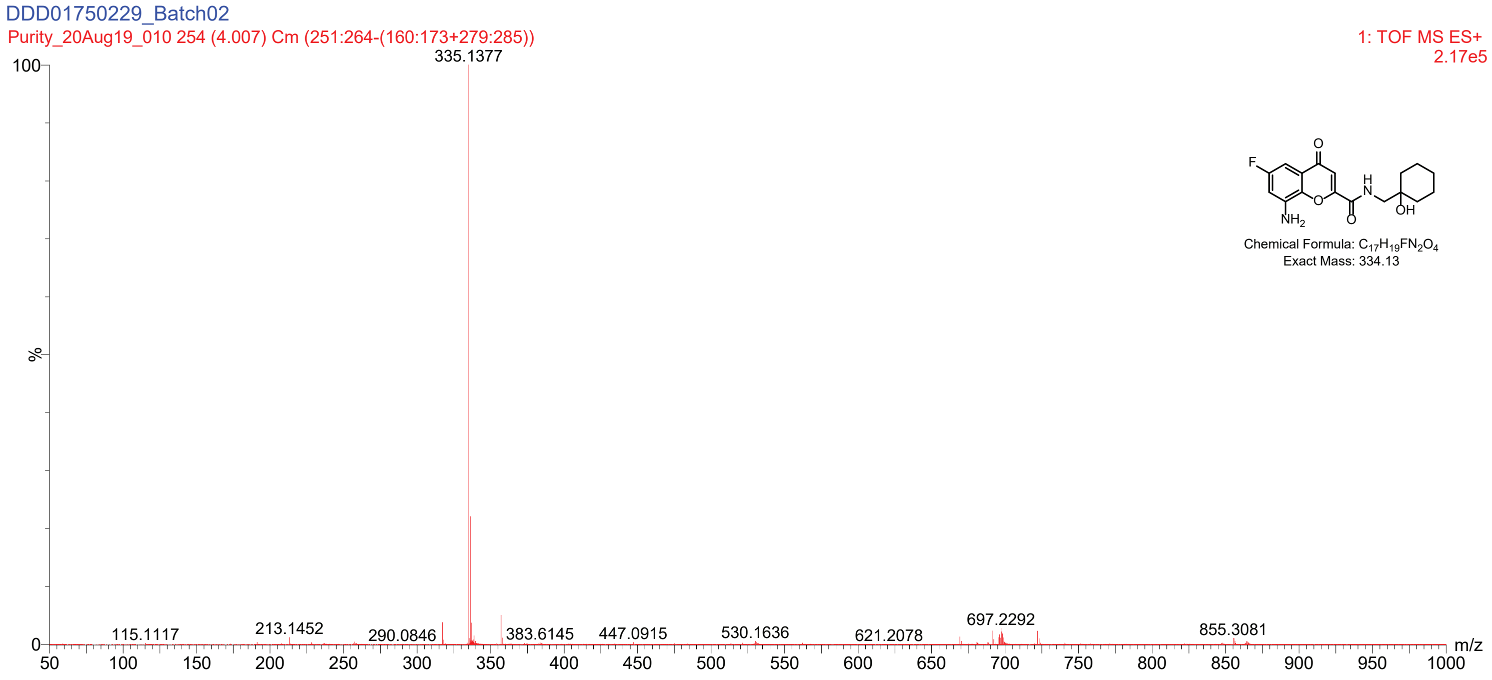
DDD01750229_Batch02
Purity_20Aug19_010 254 (4.007) Cm (251:264-(160:173+279:285))
1: TOF MS ES+
2.17e5
335.1377
100
%
0
115.1117
213.1452
290.0846
383.6145
447.0915
530.1636
621.2078
697.2292
855.3081
m/z
F
O
H
N
O
OH
NH2
Chemical Formula: C17H19FN2O4
Exact Mass: 334.13
