# Supplementary material for: Cryptosporidium lysyl-tRNA synthetase inhibitors define the interplay between solubility and permeability required to achieve efficacy
Source: Sci Transl Med. Author manuscript; Available in PMC 2025 Mar 4. (PMC7617456; doi:10.1126/scitranslmed.adm8631)
Supplement: Supplemental information [file EMS199745-supplement-Supplemental_information.pdf]

Materials and methods

Aqueous solubility

As previously described.*(27)*

Test compounds were dissolved in DMSO to give 10 mM stock solutions. Solubility test samples were prepared by adding 5 µL stock to PBS pH 7.4 (195 µL). This solution was mixed for 24 h (rotary mixing, 900 rpm, 25 °C) excluding light. The solubility test samples were vacuum filtered (Millipore Multiscreen HTS filter, 96-well format) to remove any undissolved material. The filtrate was analyzed for dissolved drug compound using a truncated UHPLC methodology. A calibration solution was prepared for each test compound. The 10 mM stock solution was diluted in DMSO to give a 500 µM solution. This was further diluted with 50:50 acetonitrile:water to give a 50 µM solution. Aliquots (0.2, 2.0, and 5.0 µL) of this 50 µM solution were injected onto the UHPLC system, and the areas of the resultant peaks were integrated to produce a calibration line. Aliquots of the test sample filtrate (0.4 and 5.0 µL) were injected onto the UHPLC system and the resultant test compound peak areas quantified using the calibration line.

PAMPA permeability

As previously described.*(28)*

The permeability assay was performed using a 96-well precoated BD Gentest PAMPA plate (BD Biosciences). Each well was divided into two chambers: donor and acceptor, separated by a lipid-oil-lipid trilayer constructed in a porous filter. Apparent permeability, Papp, of the compound was measured at pH 7.4. Stock solutions (5 mM) of the compound were prepared in DMSO. The compound was further diluted to 10 µM in phosphate-buffered saline at pH 7.4. The final DMSO concentration did not exceed 5% v/v. The compound dissolved in phosphate buffered saline was added to the donor side of the membrane, and phosphate-buffered saline without the compound was added to the acceptor side. The PAMPA plate was left at room temperature for 5 h then an aliquot (100 µL) was removed from both acceptor and donor compartments and mixed with acetonitrile (80 µL) containing an internal standard: donepezil at 50 ng/mL. The samples were centrifuged (10 min, 5 °C, 3270×g) to sediment lipids and sealed prior to UPLC-MS/MS analysis using a Quattro Premier XE (Waters Corp, USA). Recovery of the compound from donor and acceptor wells was calculated, and data were only accepted when recovery exceeded 70%.

Biochemistry

Protein expression and purification of recombinant *Cp*KRS (Dundee)

As previously described.*(8)*

Protein expression and purification of recombinant *Hs*KRS (Dundee)

As previously described.*(8)*

*Cp*KRS *in vitro* enzyme assay (Dundee)

Potency of compounds was determined in 10-point dose-response inhibition curves. Compounds were plated into 384-well clear-bottom plates (Greiner Bio-One, 781101) using an Echo 550 acoustic dispenser (Labcyte). KRS reactions were started by adding enzyme into wells containing substrate and compounds using a Thermo Scientific Matrix Wellmate. KRS assays were run in 50 µL reactions (Final concentration: 100 mM HEPES*K pH 7.4, 20 mM $MgCl_2$, 1 mM DTT, 0.5 mM ATP, 1.2 mM lysine, 250 nM *Cp*KRS and 0.5 U/mL Pyrophosphatase) at room temperature for 7 h. Reactions were stopped by the addition of Biomol green (50 µL: Enzo Life Sciences) with the amount of free phosphate detected by measuring absorbance (650 nm) after 15 min further incubation (BMG Pherastar plate reader). Samples were all run in duplicate and data was processed and analyzed using ActivityBase (IDBS).

*Hs*KRS *in vitro* enzyme assay (Dundee)

As for the *Cp*KRS assay except the final assay conditions were 30 mM Tris-HCl 8.0, 40 mM $MgCl_2$, 140 mM NaCl, 30 mM KCl, 0.01% Brij-35, 1 mM DTT, 3.5 µM ATP, 6 µM lysine, 400 nM *Hs*KRS and 0.5 U/mL Pyrophosphatase, incubations were at room temperature for 5 h.

Cell biology

Cryptosporidium in vitro inhibition (Vermont and Dundee)

HCT-8 cells (ATCC catalogue #CCL-244; RRID: *CVCL_2478*) were cultured with *C. parvum* at 37°C, 5% CO_2 in 96-well plates (transgenic parasites measured by NLuc assay as described previously)*(12)* or 384-well plates (wild type *C. parvum* Iowa isolate from Bunch Grass Farms, USA; or wild type *C. hominis* strain Tu502 from Tufts University) measured by image-based assay.*(29)*

Compounds were prepared in 100% DMSO and added at the time of infection (NLuc) or 3 hours post infection (imaging assays). Compounds were prepared at 1% final concentration DMSO in culture. Parasites were quantified at 48 hours post infection. NanoLuciferase was measured using a GloMax Discovery, Promega.

A Nikon Eclipse Ti2000 epifluorescence microscope with an automated stage was programmed to focus on the center of each well and take a 3×3 composite image using an EXi blue fluorescence microscopy camera (QImaging) with a 20X objective (NA = 0.45). Nuclei and parasite images were separately exported as .tif files, and parasites and host cells were counted using custom NIH ImageJ macros as reported previously.*(29)* Dose−response curves were plotted, and half maximal effective concentrations (EC_{50}) and 90% effective concentrations (EC_{90}) were calculated using GraphPad Prism software version 6.01.

HepG2 *in vitro* cytotoxicity (Dundee)

As previously described.*(27)*

In vitro DMPK

FaSSIF solubility (Dundee)

As previously described.*(30)*

MDCK-mdr1 permeability (Dundee)

CHIlogD (Dundee)

As previously described.*(8)*

Microsomal clearance (Dundee)

As previously described.*(31)*

Hepatocyte clearance (Dundee)

As previously described.*(31)*

Plasma protein binding (Dundee)

As previously described.*(31)*

In vitro toxicity

CYP450 inhibition (Dundee)

As previously described.*(31)*

Mini-Ames (WuXi App Tec)

DDD489 and DDD508 were evaluated for their ability to induce reverse mutations both in the presence and absence of S9 mix at the histidine locus in the genome of four strains of *Salmonella typhimurium* (TA98, TA100, TA1535, and TA97a) and at the tryptophan locus in the genome of *Escherichia coli* WP2 *uvr*A in a mini-Ames study conducted at WuXi App Tec (Suzhou).

hERG inhibition (WuXi App Tec)

DDD489 and DDD508 were evaluated for their effects on cloned hERG potassium channels stably expressed in Chinese Hamster Ovary (CHO) cells using manual patch-clamp technique in a hERG inhibition assay conducted at WuXi App Tec (Suzhou).

Mitochondrial toxicity (Cyprotex)

DDD489 and DDD508 were evaluated for their effects on mitochondrial function in a functional mitochondrial toxicity assay using Seahorse XFe96 flux analyser conducted at Cyprotex Discovery Ltd.

44 screen safety panel (Eurofins)

DDD489 was evaluated for off-target effects in a SafetyScreen44 panel at Eurofins Cerep.

In vivo DMPK

Ethics statements

All regulated procedures, at the University of Dundee, on living animals were carried out under the authority of a project license issued by the Home Office under the Animals (Scientific Procedures) Act 1986, as amended in 2012 (and in compliance with EU Directive EU/2010/63). License applications will have been approved by the University's Ethical Review Committee (ERC) before submission to the Home Office.

All mouse efficacy studies were performed at the University of Vermont in compliance with animal care guidelines and were approved by the University of Vermont Institutional Animal Care and Use Committee.

All animal experiments conducted at Moredun Research Institute (MRI) are subject to review by the Moredun Research Institute Animal Welfare and Ethical Review Body (AWERB) in accordance with UK Home Office legislation.

The protocol and any amendments or procedures involving the care or use of animals on this study had been reviewed and approved by WuXi AppTec Institutional Animal Care and Use Committee (IACUC) prior to the initiation of such procedures. A staff veterinarian monitored the study for animal welfare issues.

Accurate purity measurement of *in vivo* compounds (Dundee)

Compounds were dissolved in LCMS grade DMSO to give a 1.0 mg/mL solution. A volume (50 μL) of this solution was then diluted to 1 mL with 50/50 ACN/H_2O (both LCMS grade) to give a 50 μg/mL final solution. A 2 μL volume of this sample solution was injected to obtain an estimate of the purity by UV and accurate mass data.

To determine the purity by UV, measurements were made using the full diode array trace (λ = 210-400 nm), λ = 254 nm ('standard' wavelength for purity measurements) and the peak of maximum wavelength in each UV spectrum. Purity (%) was calculated as the average peak area of these three measurements and accurate mass data was used to confirm the correct molecular formula. Compounds were suitable for *in vivo* dosing if they had an average purity $\geq$ 98% with no impurities $\geq$ 0.5%.

Mouse pharmacokinetics (Dundee)

As previously described.*(27)*

Test compound was dosed as a bolus solution intravenously (IV) at 3mg/kg (dose volume: 5 mL/kg; dose vehicle: 10% DMSO; 40% PEG 400; 50% Milli-Q water) or dosed orally (PO) by gavage as a suspension at 10mg/kg (dose volume: 10 mL/kg; dose vehicle: 1% CMC to female BALB/C mice (n = 3/dose route). Blood samples were taken from each mouse at 5, 15, 30 min, 1, 2, 4, 6, 8 h mixed with nine volumes of Milli-Q water and stored frozen until UPLC/MS/MS analysis. Pharmacokinetic parameters were derived from the blood concentration-time curve using PK solutions software v2.0 (Summit Research Services, USA).

Rat pharmacokinetics (Dundee)

As for mice except using male Hans Wistar rats (IV dose volume 1 mL/kg; PO dose volume: 5mL/kg).

Dog pharmacokinetics (WuXi App Tec)

As for mouse except using male beagle dogs (IV dose volume: 3 mg/mL; dose vehicle: 10% DMSO, 40% PEG400, 50% (40%HP-β-CD in water); PO dose volume: 2 mg/mL; dose vehicle: 1% CMC-Na(800-1200)+0.2% Tween 80 in water). Pharmacokinetic parameters were derived from the blood concentration-time curve using WinNonLin Professional Software v 6.3 (Pharsight Corporation).

Calf efficacy and tolerability study

Calf tolerability study (Moredun Scientific)

A tolerability study was conducted on uninfected calves prior to efficacy studies to confirm palatability and tolerance. Starting at day 2 post birth ("Day 1"), DDD489 or DDD508 were administered to four male calves per treatment group. The test compound was dosed at 15 mg/kg (maximum volume of 20 ml per occasion; prepared in vehicle: 0.5% HPMC, 0.4% Tween 80, 0.5% Benzyl alcohol in sterile water) for a total of 7 days. Administration was by oral gavage twice daily immediately prior to milk feeds, and at least 8 hours apart (± 30 minutes). Body weight was measured on day 0 and day 7, and clinical observations were recorded twice a day during dosing. Blood samples were collected from all animals prior to the morning treatment on Day 1, then 5 (± 2) minutes, 15 (± 2) minutes, 30 (± 5) minutes, 1, 2, 4, 8 and 24 hours (± 10 minutes) post first daily dose. This sample schedule was repeated on Day 7 for the same animals. Blood samples were processed to plasma. Both test compounds were determined to be well tolerated by calves.

Randomisation and blinding in calf efficacy study (Moredun Scientific)

For efficacy testing, assignment to control or treatment groups was determined by random number generator software (www.random.org) in advance of the study and applied in sequence upon arrival of calves at Moredun Scientific. Fecal samples were similarly assigned random numbers blinded to treatment group in advance of the study. Control and treatment groups were housed in separate barns; within the barn calves were housed in adjacent, individual pens in straw. To minimize contamination between control and treatment groups, access to the facility

was limited to staff involved in the study, and separate PPE was dedicated for use with each group.

Specified staff were involved in preparing the vehicle and treatment material and were not involved in its administration. These staff were not involved in taking and recording clinical observations or collecting samples. Staff that administered the vehicle and treatment materials were blinded to the material identity. If staff involved in administration of materials or sample collection became "unblinded" at any point in the study, they were immediately removed from the study.

Quantification of *Cryptosporidium* shedding from calf samples (Moredun Scientific and Dundee)

Oocyst per gram from calf fecal samples was determined by microscopy (performed at MRI) and by qPCR (Pawlowic Lab at the University of Dundee). For microscopy 0.1 g of fecal material was resuspended in 10 ml PBS. 100 microliters of diluted fecal material was incubated with 15 microliters *Cryptosporidium* FITC Stain (CellLabs) and incubated at 37˚ C for 15 minutes. Samples were then mounted on a grided slide and counted using a fluorescent microscope. For qPCR, fecal samples were continuously stored in 4°C and transported to the University of Dundee. DNA was extracted from 100mg of fecal material (unless otherwise noted) using the Zymo Quick-DNA Fecal/Soil Microbe Miniprep Kit (Cambridge Bioscience, catalog #D6010). Once material was suspended in Bashing Bead Buffer we modified the protocol slightly by adding 5 freeze/thaw cycles.*(32)* DNA was stored in 4°C to await qPCR. For the standard curve, known amounts of wild-type oocysts were added to 100 mg of uninfected murine fecal sample and DNA was extracted using the protocol detailed above. We used qPCR primers and probe designed to selectively amplify the 18s region of *C. parvum* (see Table S5 for primers*(33)*). We used the Luna Universal Probe qPCR Master Mix (NEB, catalog #M3004S) and the QuantStudio 3 Real-Time PCR System (Thermo Fisher) to measure amplification as compared to standard curve.

DNA sequencing of calf efficacy study samples (Dundee)

Three of the 7 treated cows had positive *C. parvum* amplification from day 15 onwards via qPCR. To verify that the *C. parvum* was not resistant to treatment, we amplified the KRS ORF from the extracted fecal DNA (see Table S5 for primers). Purified PCR product was sent for Sanger Sequencing (Azenta) and analyzed with Benchling [Biology Software]. (2022). Retrieved from https://benchling.com.

Target validation studies

Sample preparation and protein extraction (Dundee)

Samples of wild type oocysts and OE-KRS oocysts were prepared as two biological repeats (with two technical repeats each) and analyzed using quantitative proteomics. 40 million oocysts per technical repeat were bleached, washed three times in PBS, and excysted at 37 ºC in 0.2 mM sodium taurochlorate for 2 hours. Excysted material was resuspended in 25 µl lysis buffer (4% SDS, 10 mM DTT, 1x protease inhibitor cocktail, all prepared in water). Sample was subjected to five cycles of freeze thaw to excyst remaining oocysts. Samples were incubated at 56 ºC for 1 hour while shaking (800 rpm) to solubilize proteins. Samples were centrifuged at 20,000xg for 40 minutes at 4 ºC. Added TCEP (tris(2-carboxyethyl) phosphine) to a final concentration of 25 mM and incubated at 37 ºC for 10 minutes. Added IAA (iodoacetamide) to a final concentration of 25 mM and incubated at room temperature in the dark for 1 hour. Added 13% volume ice-cold TCA and store at -20 C. Sample was centrifuged at 16,000 xg for 4 ºC for 5 minutes. Supernatant was removed and pellet was washed with 0.5 ml cold acetone. Acetone wash repeated a total of five times and then the sample was air dried. Protein extract was resuspended in 400 µl TEAB, sonicated to resuspend the material. Sample was digested with Trypsin/LysC (Promega) overnight at 37 ºC with shaking, and then dried.

TMT labelling and high pH reverse phase fractionation (Dundee)

Tryptic peptides (11.6µg, from each sample) were dissolved in 100 µl of 150 mM TEAB. TMT labelling was performed according to the manufacturer's instructions (Thermo-Fisher Scientific). The different TMT-10 plex labels (0.8mg) (Thermo Fisher Scientific) were dissolved in 41µL of anhydrous acetonitrile, and each label is added to a different sample. The mixture was incubated for 1 hour at room temperature, an equivalent of 0.75 µg of peptides from each sample were mixed with 20 µl 1% formic acid and used to check labelling efficiency. The remaining samples were kept at -80ºc, until the labelling efficiency was checked. Samples were pooled, desalted, and dried in a speed-vac at 30°C. Samples were re-dissolved in 200 µl ammonium formate (10mM, pH 9.5) and peptides were fractionated using High pH RP Chromatography. A C18 Column from Waters (XBridge peptide BEH, 130Å, 3.5 µm 2.1 X 150 mm, Waters, Ireland) with a guard column (XBridge, C18, 3.5 µm, 2.1X10mm, Waters) were used on an Ultimate 3000 HPLC (Thermo-Scientific). Buffers A and B used for fractionation consist, respectively, of (A) 10 mM ammonium formate in milliQ water pH 9.5 and (B) 10 mM ammonium formate, pH 9.5 in 90% acetonitrile. Fractions were collected using a WPS-3000FC

auto-sampler (Thermo-Scientific) at 1-minute intervals. Column and guard column were equilibrated with 2% Buffer B for twenty minutes at a constant flow rate of 0.2ml/min. Fractionation of TMT labelled peptides was performed as fellows; 190 µl aliquot were injected onto the column, and the separation gradient was started 1 minute after the sample was loaded onto the column. Peptides were eluted from the column with a gradient of 2% Buffer B to 20% Buffer B in 6 minutes, then from 20% Buffer B to 45% Buffer B in 51 minutes and finally from 45% buffer B to 100% Buffer B within 1 min. The Column was washed for 15 minutes in 100% Buffer B. The fraction collection started 1 minute after injection and stopped after 80 minutes (total 80 fractions, 200µl each). Formic acid (30 µl of 10% stock) was added to each fraction and concatenated in groups of 20 fractions.

LC-MS analysis (Dundee)

Analysis of peptides was performed on a Q-exactive-HF (Thermo Scientific) mass spectrometer coupled with a Dionex Ultimate 3000 RS (Thermo Scientific). LC buffers were the following: buffer A (0.1% formic acid in Milli-Q water (v/v), buffer B (80% acetonitrile and 0.1% formic acid in Milli-Q water (v/v) and loading buffer (0.1% TFA).

Peptides from each fraction were resuspended in 50 µl 1% formic acid and aliquots of 5 µL were loaded at 10 µL/min onto a trap column (100 µm × 2 cm, PepMap nanoViper C18 column, 5 µm, 100 Å, Thermo Scientific) equilibrated in 0.1% TFA. The trap column was washed for 5 min at the same flow rate with 0.1% TFA and then switched in-line with a Thermo Scientific, resolving C18 column (75 µm × 50 cm, PepMap RSLC C18 column, 2 µm, 100 Å) equilibrated in 5% buffer B for 17 min. The peptides were eluted from the column at a constant flow rate of 300 nl/min with a linear gradient from 5% buffer B (for Fractions 1-10, 7% for Fractions 11-20) to 35% buffer B in 125 min, and then from 35% buffer B to 98% buffer B in 2 min. The column was then washed with 98% buffer B for 20 min and re-equilibrated in 5% or 7% buffer B for 17 min. The column was maintained at a constant temperature of 50ºC.

Q-exactive HF was operated in data dependent positive ionisation mode. The source voltage was set to 2.85 Kv and the capillary temperature was 250ºC. A scan cycle comprised MS1 scan (m/z range from 335-1600, with a maximum ion injection time of 50 ms, a resolution of 120 000 and automatic gain control (AGC) value of $3x10^6$) followed by 15 sequential dependant MS2 scans (resolution 60000) of the most intense ions fulfilling predefined selection criteria (AGC $1x10^5$, maximum ion injection time 200 ms, isolation window of 0.7 m/z, fixed first mass of 100 m/z, spectrum data type: centroid, intensity threshold 5×10^4, exclusion of

unassigned, singly and >6 charged precursors, peptide match preferred, exclude isotopes on, dynamic exclusion time of 45 s). The HCD collision energy was set to 32% of the normalized collision energy. Mass accuracy is checked before the start of samples analysis.

Quantitative proteomics analysis of transgenic *Cryptosporidium* (Dundee)

Proteomic data were processed through MaxQuant software (version 2.4.10.0), leveraging its integrated Andromeda search engine engine.*(34)* The search database was specifically constructed for *Cryptosporidium parvum* Iowa II, with annotated protein sequences obtained from CryptoDB,*(35)* release 61. This was augmented with sequences for the reporter proteins mScarletI and Nluc-Neo. Additionally, a murine protein database, retrieved from Uniprot*(36)* on January 20, 2023, was concatenated with the *C. parvum* database to account for potential host protein contamination. The analysis encompassed both TMT 6-plex and TMT 10-plex labeling, processed in parallel within a single MaxQuant instance. Each TMT experiment was treated as a distinct analytical batch, with no normalization applied between them. Trypsin was designated as the proteolytic enzyme, with a specification for up to two missed cleavages per peptide allowed. Carbamidomethylation of cysteine residues was set as a constant post-translational modification (PTM), whereas N-terminal acetylation of proteins and methionine oxidation were configured as variable PTMs. Default settings were retained for all other parameters within MaxQuant, except for TMT label correction factors. These were adjusted according to the manufacturer's instructions (Thermo Fisher Scientific) and are detailed within the MaxQuant parameter files. These files, alongside the raw data, have been submitted to the PRIDE database (PRIDE submission: PXD050089).

Pre-normalization, we filtered the MaxQuant output to exclude potential confounders. Data were refined by removing entries solely identified by peptide modification sites (Only identified by site), entries marked as reverse database matches (Reverse), and proteins classified as potential contaminants (Potential contaminant). For our analysis of *Cryptosporidium parvum* lysyl-tRNA synthetase (*Cp*KRS) across wild-type (WT) and knockout (KO) specimens, we used a robust normalization technique tailored specifically for TMT proteomic data. Our experimental set consisted of one biological replicate with two technical replicates integrated within a TMT 6-plex configuration, along with an additional biological replicate paired with two technical replicates in a TMT 10-plex setup. To normalize the signal of the two TMT batches, we adopted a modified version of the Internal Reference Standard (IRS) normalization method, as initially delineated by Plubell et al.*(37)* The IRS normalization was applied by taking the raw mean intensity of each plex to serve as the

reference channel. We computed the sum of the intensities for each TMT channel row-wise and then calculated the geometric mean of these sums to establish a stable reference point. This method was chosen to replace the reference channel utilized in *(37)*, which was not generated from our datasets. The scaling factors for normalization were ascertained by dividing this geometric mean by the sum intensities of each individual experiment. Subsequently, each plex underwent individual scaling to ensure uniformity in signal intensities. Upon normalizing the data, we employed Principal Component Analysis (PCA) to verify the efficacy of the IRS process between the TMT 6-plex and 10-plex setups. The differential expression analysis was performed with the limma package*(38)* using the WT samples versus the KO samples, with log2 values. FDR values were computed with the toptable function in limma. The output table of the analysis is available online as **data file S1** (csv file).

Chemistry experimental

General information: equipment and methods

Solvents and reagents were purchased from commercial suppliers and used without further purification. Dry solvents were purchased in sure sealed bottles stored over molecular sieves. Unless otherwise stated herein, reactions have not been optimised. Yields refer to chromatographically and spectroscopically pure compounds. Column chromatography was performed using Combiflash Companion Rf (commercially available from Teledyne ISCO) and prepacked silica gel columns purchased from Teledyne ISCO. Mass-directed preparative HPLC separations were performed using a Waters HPLC (2545 binary gradient pumps, 515 HPLC make up pump, 2767 sample manager) connected to a Waters 2998 photodiode array and a Waters 3100 mass detector. Preparative HPLC separations were performed with a Gilson HPLC (321 pumps, 819 injection module, 215 liquid handler/injector) connected to a Gilson 155 UV/vis detector. On both instruments, HPLC chromatographic separations were conducted using Waters XBridge C18 columns, 19 x 100 mm, 5 um particle size; using 0.1% ammonia in water (solvent A) and acetonitrile (solvent B) or 0.1% formic acid in water (solvent A) and acetonitrile (solvent B) as mobile phase. Supercritical fluid chromatography (SFC) chiral separation was performed on a Waters SFC 350 using a Daicel Chiralpak column. ^{1}H, ^{13}C, and ^{19}F NMR spectra were recorded on a Bruker Avance DPX 500 spectrometer (^{1}H at 500.1 MHz, ^{13}C at 125.8 MHz, ^{19}F at 470.5 MHz), or a Bruker Avance DPX 400 (^{1}H at 400 MHz, ^{13}C at 100.6 MHz). Chemical shifts (δ) are expressed in parts per million (ppm), recorded using the residual solvent as the internal reference in all cases. Signal splitting patterns are described as

singlet (s), doublet (d), triplet (t), quartet (q), multiplet (m), broad (br), or a combination thereof. Coupling constants (*J*) are quoted to the nearest 0.1 Hz. ^{19}F NMR spectra are ^{1}H and ^{13}C-decoupled. Low resolution electrospray (ES) mass spectra were recorded on an Agilent HPLC 1100 series connected to a Bruker Daltonics MicrOTOF or an Agilent Technologies 1200 series HPLC connected to an Agilent Technologies 6130 quadrupole LC/MS, where both instruments were connected to an Agilent diode array detector. High resolution mass spectroscopy (HRMS) was performed using a Bruker MicroTof mass spectrometer or Thermo Exploris 120 Orbitrap. NMR and mass spectra for all final compounds are reported in the attached appendix.

Lactam series synthesis

2-amino-4-(trifluoromethyl)-6-(((1R,3S)-3-(trifluoromethyl)cyclohexyl)methyl)-6,7-dihydro-5H-pyrrolo[3,4-d]pyrimidin-5-one (DDD489)

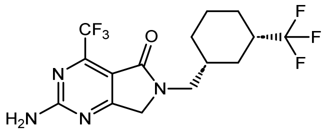

The product was synthesised as previously described.*(39)* ^{1}H NMR (500 MHz, DMSO-d_6) δ 7.93 – 7.89 (m, 2H), 4.44 – 4.35 (m, 2H), 3.38 – 3.30 (m, 2H), 2.28 – 2.22 (m, 1H), 1.82 – 1.78 (m, 4H), 1.66 (br. d, *J* = 12.9 Hz, 1H), 1.32 – 1.25 (m, 1H), 1.20 – 1.13 (m, 1H), 0.97 – 0.88 (m, 2H). ^{13}C NMR (125 MHz, DMSO-d_6) δ 176.3, 163.8, 162.5, 150.8 (q, $^{2}J_{CF}$ = 37.6 Hz), 127.8 (q, $^{1}J_{CF}$ = 277.3 Hz), 119.9 (q, $^{1}J_{CF}$ = 274.7 Hz), 109.0, 51.2, 47.5, 40.3 (q, $^{2}J_{CF}$ = 25.7 Hz), 34.8, 29.3, 28.6, 24.3, 23.5. ^{19}F NMR (470 MHz, DMSO-d6) δ -66.15, -72.36. HRMS (ES+) calcd. for $C_{15}H_{17}F_6N_4O$ $[M + H]^+$ 383.1307, found 383.1305. Purity: 99.97%

2-amino-6-(spiro[2.5]octan-5-ylmethyl)-4-(trifluoromethyl)-6,7-dihydro-5H-pyrrolo[3,4-d]pyrimidin-5-one (DDD909)

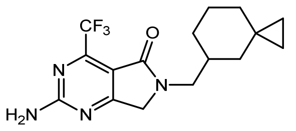

The product was synthesised as previously described.*(39)* ^{1}H NMR (500 MHz, DMSO-d_6) δ 7.91 – 7.88 (m, 2H), 4.36 (d, *J* = 4.1 Hz, 2H), 3.30 – 3.27 (m, 2H), 1.88 – 1.80 (m, 1H), 1.68 – 1.64 (m, 2H), 1.61 – 1.56 (m, 1H), 1.39 – 1.32 (m, 2H), 0.99 – 0.83 (m, 3H), 0.26 – 0.23 (m, 2H), 0.21 – 0.14 (m, 2H). ^{13}C NMR (100 MHz, DMSO-d_6) δ 176.3, 163.7, 162.4, 150.7 (q,

$^{2}J_{CF}$ = 37.5 Hz), 120.4, 119.9 (q, $^{1}J_{CF}$ = 298.1), 109.1, 51.0, 47.3, 35.3, 35.0, 29.7, 24.2, 18.2, 11.8, 11.7. ^{19}F NMR (470 MHz, DMSO-d6) δ -66.09. HRMS (ES+) calcd. for $C_{16}H_{20}F_3N_4O$ $[M + H]^+$ 341.1589, found 341.1583. Purity: 99.74%

2-amino-4-(difluoromethyl)-6-(((1R,3S)-3-(trifluoromethyl)cyclohexyl)methyl)-6,7-dihydro-5H-pyrrolo[3,4-d]pyrimidin-5-one (DDD352)

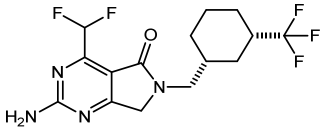

The product was synthesised as previously described.*(39)* ^{1}H NMR (500 MHz, DMSO-d6) δ 7.86 – 7.71 (m, 2H), 7.27 (t, J = 53.8 Hz, 1H), 4.45 – 4.35, (m, 2H), 3.33 – 3.26 (m, 2H), 2.28 – 2.20 (m, 1H), 1.83 – 1.77 (m, 4H), 1.66 – 1.64 (m, 1H), 1.31 – 1.23 (m, 1H), 1.19 – 1.16 (m, 1H), 0.96 – 0.87 (m, 2H). ^{13}C NMR (100 MHz, DMSO-d_6) δ 174.9, 164.6, 164.3, 156.1 (t, $^{2}J_{CF}$ = 22.8 Hz), 127.9 (q, $^{1}J_{CF}$ = 278.5 Hz), 110.2, 108.6 (t, $^{1}J_{CF}$ = 238.7 Hz), 51.4, 47.3, 40.4, 34.8, 29.3, 28.6, 24.3, 23.6. ^{19}F NMR (470 MHz, DMSO-d_6) δ -72.3, -122.0 (d, J = 21.4 Hz). HRMS (ES+) calcd. for $C_{15}H_{18}F_5N_4O$ $[M+H]^+$ 365.1323, found 365.1402. Purity: 99.44%

2-amino-4-methyl-6-(((1R,3S)-3-(trifluoromethyl)cyclohexyl)methyl)-6,7-dihydro-5H-pyrrolo[3,4-d]pyrimidin-5-one (DDD478)

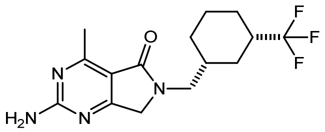

The product was synthesised as previously described.*(39)* ^{1}H NMR (400 MHz, DMSO-d_6): δ 7.17 (br. s, 2H), 4.29 – 4.18 (m, 2H), 3.36 – 3.31 (m, 1H), 3.27 – 3.23 (m, 1H), 2.47 (s, 3H), 2.31 – 2.20 (m, 1H), 1.84 – 1.73 (m, 4H), 1.63 (br. d, J = 12.7 Hz, 1H), 1.33 – 1.10 (m, 2H), 0.96 – 0.86 (m, 2H). ^{13}C NMR (100 MHz, DMSO-d_6): δ 173.3, 166.3, 165.3, 164.1, 127.9 (q, $^{1}J_{CF}$ = 250.4 Hz), 110.8, 50.6, 47.0, 34.9, 29.3, 28.6, 24.3, 23.6, 19.6. ^{19}F NMR (470 MHz, DMSO-d6) δ -72.34. HRMS (ES+) calcd. for $C_{15}H_{20}F_3N_4O$ $[M+H]^+$ 329.1589, found 329.1599. Purity: 99.91%

Ethyl 2-amino-4-(bromomethyl)pyrimidine-5-carboxylate

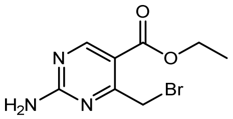

To a solution of ethyl 2-amino-4-methyl-pyrimidine-5-carboxylate (9 g, 49.7 mmol) in AcOH (331 mL) was added Br_2 (1.8 mL, 34.8 mmol) and the reaction mixture was stirred at 20 °C for 30 min then heated at 60 ºC for 1 h. The reaction mixture was concentrated then diluted with sat. $Na_2S_2O_3$ (50 mL) and extracted with ethyl acetate (3 × 50 mL). The combined organics were washed with sat. $Na_2S_2O_3$ (50 mL), dried, filtered, and concentrated under vacuum. The product was purified by column chromatography (SiO_2, 0:100→30:70 EtOAc:heptanes) to afford ethyl 2-amino-4-(bromomethyl)pyrimidine-5-carboxylate (2.7 g, 20% yield) as a white solid. ^{1}H NMR (500 MHz, DMSO-d_6) δ 8.74 (s, 1H), 7.61 (br. s, 2H), 4.70 (s, 2H), 4.27 (q, *J* =7.1 Hz, 2H), 1.31 (t, *J* = 7.1 Hz, 3H). MS (ESI): *m/z* (%) 260/262 (94) $[M+H]^+$.

2-Amino-6-(cyclohexylmethyl)-7H-pyrrolo[3,4-d]pyrimidin-5-one (DDD212)

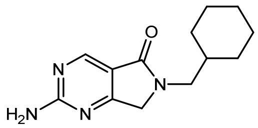

To a solution of benzylamine (150 µL, 1.15 mmol) in DMF (2 mL) at 20 °C was added a solution of ethyl 2-amino-4-(bromomethyl)pyrimidine-5-carboxylate (100 mg, 0.38 mmol) in DMF (2 mL) dropwise. The reaction mixture was stirred at 20 °C for 30 min then a solution of lithium hydroxide monohydrate (16.1 mg, 0.38 mmol) in water (0.5 mL) was added and the reaction mixture was stirred at 20 °C for 17 h. A further solution of lithium hydroxide monohydrate (16.1 mg, 0.38 mmol) in water (0.5 mL) was added and the reaction mixture was stirred at 20 °C for 3 h. The reaction mixture was concentrated under vacuum. The product was purified by column chromatography (SiO_2, 0:100→10:90 MeOH:DCM) to afford 2-amino-6-(cyclohexylmethyl)-7H-pyrrolo[3,4-d]pyrimidin-5-one (34 mg, 34% yield) as a colourless solid. ^{1}H-NMR (400 MHz, DMSO-d_6) δ 8.47 (s, 1H), 7.34 (s, 2H), 4.28 (s, 2H), 3.25 (d, *J* = 7.3 Hz, 2H), 1.67 - 1.58 (m, 6H), 1.23 - 1.12 (m, 3H), 0.95 - 0.86 (m, 2H). ^{13}C-NMR (400 MHz, DMSO-d_6) δ 173.3, 165.4, 164.7, 153.7, 113.3, 50.9, 47.3, 36.0, 30.2, 26.0, 25.1. HRMS (ES+): calcd. for $C_{13}H_{19}N_4O$ $[M+H]^+$ 247.1559, found 247.1565. Purity: 99.92%

Chromone series synthesis

6-hydroxy-N-((1-hydroxycyclohexyl)methyl)-4-oxo-4H-chromene-2-carboxamide (DDD508)

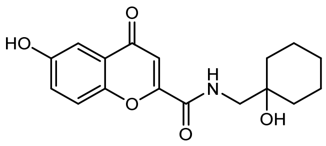

The product was synthesised as previously described.*(40)* ^{1}H-NMR (500 MHz, DMSO-d_6): δ 10.10 (s, 1H), 8.61 (t, J = 6.2 Hz, 1H), 7.64 – 7.63 (m, 1H), 7.32 – 7.30 (m, 2H), 6.77 (s, 1H), 4.37 (s, 1H), 3.30 – 3.28 (m, 2H), 1.59 – 1.53 (m, 2H), 1.48 – 1.33 (m, 7H), 1.24 – 1.18 (m, 1H). ^{13}C-NMR (125 MHz, DMSO-d_6): δ 177.1, 159.5, 155.4, 155.2, 148.7, 124.5, 123.8, 120.2, 109.3, 107.3, 70.2, 49.3, 34.9, 25.3, 21.4. HRMS (ES+): calcd. for $C_{17}H_{20}NO_5$ $[M+H]^+$ 318.1341, found 318.1350. Purity: 99.11%.

N-(cyclohexylmethyl)-7-fluoro-8-hydroxy-4-oxo-4H-chromene-2-carboxamide (DDD714)

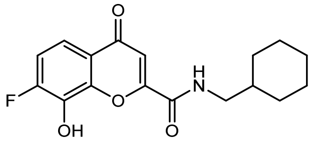

The product was synthesised as previously described.*(40)* ^{1}H-NMR (500 MHz, DMSO-d_6): δ 10.71 (s, 1H), 9.10 (t, J = 5.9 Hz, 1H), 7.51 (dd, J = 8.9, 5.7 Hz, 1H), 7.38 (dd, J = 10.4, 9.0 Hz, 1H), 6.81 (s, 1H), 3.21 (t, J = 6.5 Hz, 2H), 1.75 – 1.69 (m, 4H), 1.64 – 1.55 (m, 2H), 1.25 – 1.14 (m, 3H), 1.00 – 0.94 (m, 2H). ^{13}C-NMR (125 MHz, DMSO-d_6): δ 176.7, 158.4, 155.4, 153.6 (d, $^{1}J_{CF}$ = 246.5 Hz), 152.6, 145.8 (d, $^{3}J_{CF}$ = 7.4 Hz), 134.6 (d, $^{2}J_{CF}$ = 14.7 Hz), 120.9, 114.6, 114.4 (d, $^{2}J_{CF}$ = 13.4 Hz), 110.4, 45.3, 37.5, 30.4, 25.9, 25.3. ^{19}F-NMR (470 MHz, DMSO-d_6): δ -126.6. HRMS (ES+): calcd. for $C_{17}H_{19}NO_4F$ $[M+H]^+$ 320.1298, found 320.1301. Purity: 99.99%

7-fluoro-8-hydroxy-N-((1-hydroxycyclohexyl)methyl)-4-oxo-4H-chromene-2-carboxamide (DDD844)

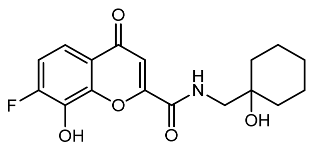

The product was synthesised as previously described.*(40)* ^{1}H-NMR (500 MHz, DMSO-d_6): δ 11.02 (s, 1H), 9.01 (t, J = 5.9 Hz, 1H), 7.51 (dd, J = 8.8, 5.7 Hz, 1H), 7.39 (dd, J = 10.4, 9.0 Hz, 1H), 6.84 (s, 1H), 4.41 (s, 1H), 3.34 (d, J = 6.4 Hz, 2H), 1.58 – 1.54 (m, 2H), 1.48 – 1.35 (m, 7H), 1.26 – 1.20 (m, 1H). ^{13}C-NMR (125 MHz, DMSO-d_6): δ 176.7, 158.8, 155.6, 153.7 (d, $^{1}J_{CF}$ = 246.0 Hz), 145.9 (d, $^{3}J_{CF}$ = 7.7 Hz), 134.7 (d, $^{2}J_{CF}$ = 14.6 Hz), 121.0, 114.5 (d, $^{2}J_{CF}$ = 10.3 Hz), 114.4, 110.6, 70.5, 49.2, 35.0, 25.3, 21.4. ^{19}F-NMR (470 MHz, DMSO-d_6): δ -126.5. HRMS (ES+): calcd. for $C_{17}H_{19}NO_5F$ $[M+H]^+$ 336.1247, found 336.1227. Purity: 99.31%

8-amino-6-fluoro-N-((1-hydroxycyclohexyl)methyl)-4-oxo-4H-chromene-2-carboxamide (DDD229)

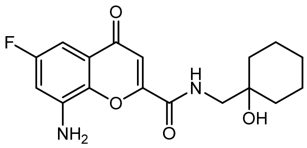

The product was synthesised as previously described.*(40)* ^{1}H-NMR (400 MHz, DMSO-d_6): 9.03 (t, J = 6.2 Hz, 1H), 6.84 – 6.80 (m, 2H), 6.76 (dd, J = 8.5, 3.0 Hz, 1H), 6.55 (s, 2H), 4.33 (s, 1H), 3.30 (d, J = 6.5 Hz, 2H), 1.59 – 1.52 (m, 2H), 1.47 – 1.32 (m, 7H), 1.23 – 1.15 (m, 1H). ^{13}C-NMR (125 MHz, DMSO-d_6): δ 177.0, 160.0 (d, $^{1}J_{CF}$ = 239.6 Hz), 158.9, 155.3, 141.4 (d, $^{3}J_{CF}$ = 12.5 Hz), 140.2, 125.1 (d, $^{3}J_{CF}$ = 9.9 Hz), 109.9, 103.7 (d, $^{2}J_{CF}$ = 28.2 Hz), 93.8 (d, $^{2}J_{CF}$ = 24.6 Hz), 70.7, 49.5, 35.0, 25.3, 21.3. ^{19}F-NMR (470 MHz, DMSO-d_6): δ -114.3. HRMS (ES+): calcd. for $C_{17}H_{20}N_2O_4F$ $[M+H]^+$ 335.1407, found 335.1377. Purity: 99.85%

Dimethyl 2-(3-fluoro-2-hydroxyphenoxy)but-2-enedioate

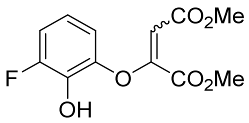

To a solution of 3-fluorobenzene-1,2-diol (27 g, 211 mmol) in DCM (50 mL) at 0 °C was added dimethyl but-2-ynedioate (25.9 mL, 211 mmol). Triethylamine (29.4 mL, 211 mmol) was added and the reaction mixture was stirred at room temperature for 16 h. The reaction mixture was diluted with DCM (50 mL), washed with 2M HCl (3 × 50 mL), passed through a hydrophobic frit, and concentrated under vacuum to afford dimethyl 2-(3-fluoro-2-hydroxy-phenoxy)but-2-enedioate (56.2 g, 99% yield) as a brown oil. ^{1}H NMR (500 MHz, DMSO-d_6) δ 6.93 – 6.87 (m, 3.6H), 5.74 (s, 0.4H), 3.78 (s, 3H), 3.64 (s, 3H). Note, 1:1.5 mixture of E:Z alkenes. Exchangeable proton was not detected.

Dimethyl 2-(3,4-difluoro-2-methoxyphenoxy)but-2-enedioate

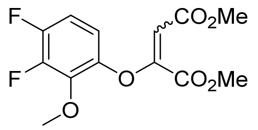

Synthesised according to procedure for dimethyl 2-(3-fluoro-2-hydroxyphenoxy)but-2-enedioate using 3,4-difluoro-2-methoxyphenol (2.1 g, 14.9 mmol) to afford dimethyl 2-(3,4-difluoro-2-methoxyphenoxy)but-2-enedioate (4.5 g, 99% yield) as a brown oil. ^{1}H NMR (400 MHz, $CDCl_3$) δ 6.93 – 6.77 (m, 1.4H), 6.66 – 6.61 (m, 0.6H), 6.51 (s, 0.6H), 5.11 (s, 0.4H), 4.01 (d, J = 1.7 Hz, 1.2H), 3.97 (d, J = 1.1 Hz, 1.8H), 3.93 (s, 1.2H), 3.77 (s, 1.8H), 3.73 (s, 1.8H), 3.69 (s, 1.2H). Note, 1:1.5 mixture of E:Z alkenes.

Dimethyl 2-(3-fluoro-2-methoxyphenoxy)but-2-enedioate

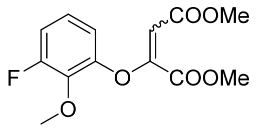

To a solution of dimethyl 2-(3-fluoro-2-hydroxy-phenoxy)but-2-enedioate (28 g, 104 mmol) and potassium carbonate (31.5 g, 228 mmol) in DMF (20 mL) was added iodomethane (14.2 mL, 228 mmol) and the reaction mixture was stirred at room temperature for 16 h. The reaction mixture was concentrated under vacuum then dissolved in EtOAc (50 mL), washed with water (5 × 50 mL), passed through a hydrophobic frit, and concentrated under vacuum to afford dimethyl 2-(3-fluoro-2-methoxy-phenoxy)but-2-enedioate (24.9 g, 85% yield) as a brown oil. ^{1}H NMR (500 MHz, DMSO-d_6) δ 7.29 – 7.16 (m, 1H), 7.06 – 6.99 (m, 1.6H), 6.75 – 6.74 (m, 0.6H), 6.57 (s, 0.6H), 5.35 (s, 0.4H), 3.87 (s, 1.2H), 3.83 (s, 1.2H), 3.82 (s, 1.8H), 3.73 (s, 1.8H), 3.65 (s, 1.8H), 3.62 (s, 1.2H). Note, 1:1.5 mixture of E:Z alkenes.

2-(3-fluoro-2-methoxyphenoxy)but-2-enedioic acid

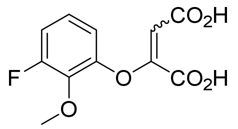

To a solution of dimethyl 2-(3-fluoro-2-methoxy-phenoxy)but-2-enedioate (24.9 g, 87.6 mmol) in water (20 mL) and methanol (20 mL) was added NaOH (17.5 g, 438 mmol) portionwise and the reaction mixture was stirred at room temperature for 16 h. The reaction mixture was acidified to pH1 with 2M HCl, extracted with EtOAc (3 × 100 mL), passed through a

hydrophobic frit, and concentrated under vacuum to afford 2-(3-fluoro-2-methoxy-phenoxy)but-2-enedioic acid (22.4 g, 99% yield) as an orange solid. ^{1}H NMR (500 MHz, DMSO-d$_6$) δ 7.24 – 7.14 (m, 1H), 7.05 – 6.94 (m, 1.6H), 6.69 – 6.67 (m, 0.6H), 6.50 (s, 0.6H), 5.10 (s, 0.4H), 3.86 (s, 1.2H), 3.82 (s, 1.8H). Note, 1:1.5 mixture of E:Z alkenes. Exchangeable protons were not detected.

2-(3,4-difluoro-2-methoxyphenoxy)but-2-enedioic acid

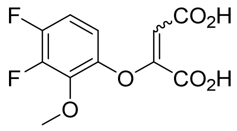

Synthesised according to procedure for 2-(3-fluoro-2-methoxyphenoxy)but-2-enedioic acid using dimethyl 2-(3,4-difluoro-2-methoxy-phenoxy)but-2-enedioate (4.5 g, 14.9 mmol) to afford 2-(3,4-difluoro-2-methoxyphenoxy)but-2-enedioic acid (4 g, 98% yield) as a yellow solid. ^{1}H NMR (500 MHz, DMSO-d$_6$) δ 7.24 – 7.14 (m, 1H), 7.05 – 6.94 (m, 1.6H), 6.69 – 6.67 (m, 0.6H), 6.50 (s, 0.6H), 5.10 (s, 0.4H), 3.86 (s, 1.2H), 3.82 (s, 1.8H). Note, 1:1.5 mixture of E:Z alkenes. Exchangeable protons were not detected.

7-fluoro-8-methoxy-4-oxo-4H-chromene-2-carboxylic acid

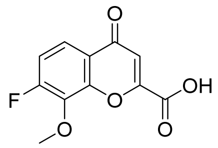

To a solution of 2-(3-fluoro-2-methoxy-phenoxy)but-2-enedioic acid (22.4 g, 87.4 mmol) in acetyl chloride (156 mL) was added sulphuric acid (4.7 mL, 87.4 mmol) dropwise and the reaction mixture was heated at 50 °C for 2 h. The reaction mixture was cooled, poured over ice, and the resulting precipitate was collected by filtration and dried under vacuum to afford 7-fluoro-8-methoxy-4-oxo-chromene-2-carboxylic acid (9.4 g, 45% yield) as a white solid. ^{1}H-NMR (500 MHz, DMSO-d_6): δ 7.78 (dd, J = 8.9, 5.7 Hz, 1H), 7.46 (dd, J = 10.3, 9.2 Hz, 1H), 6.91 (s, 1H), 4.08 (s, 3H). Note, exchangeable proton was not detected. MS (ES+): m/z (%) 239 (87) $[M+H]^+$.

6,7-difluoro-8-methoxy-4-oxo-4H-chromene-2-carboxylic acid

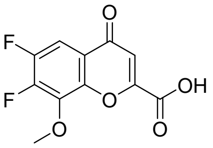

Synthesised according to procedure for 7-fluoro-8-methoxy-4-oxo-4H-chromene-2-carboxylic acid using 2-(3,4-difluoro-2-methoxy-phenoxy)but-2-enedioic acid (4 g, 14.6 mmol). The product was triturate with TBME to afford 6,7-difluoro-8-methoxy-4-oxo-4H-chromene-2-carboxylic acid (985 mg, 24% yield) as an off-white solid. ^{1}H-NMR (400 MHz, DMSO-d_6): δ 7.68 (dd, J = 9.9, 8.3 Hz, 1H), 6.93 (s, 1H), 4.16 (d, J = 1.0 Hz, 3H). Note, exchangeable proton was not detected. MS (ES+): *m/z* (%) 257 (88) $[M+H]^{+}$.

7-fluoro-8-hydroxy-4-oxo-4H-chromene-2-carboxylic acid

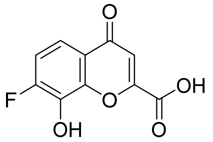

To a solution of 7-fluoro-8-methoxy-4-oxo-chromene-2-carboxylic acid (2.5 g, 10.5 mmol) in DCM (10 mL) at -78 °C was added tribromoborane (42 mL, 41.9 mmol) and the reaction mixture was stirred at room temperature for 16 h. The reaction mixture was poured over ice and the resulting precipitate was collected by filtration and dried under vacuum to afford 7-fluoro-8-hydroxy-4-oxo-chromene-2-carboxylic acid (2.04 g, 87% yield) as a yellow solid. ^{1}H-NMR (400 MHz, DMSO-d_6): δ 10.8 (br. s, 1H), 7.51 (dd, J = 8.9, 5.8 Hz, 1H), 7.38 (dd, 1H, J = 10.2, 9.1 Hz, ArH), 6.88 (s, 1H, CH).

7-fluoro-*N*-((1-fluorocyclohexyl)methyl)-8-hydroxy-4-oxo-4H-chromene-2-carboxamide (DDD993)

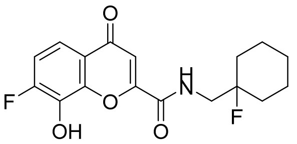

To a solution of 7-fluoro-8-hydroxy-4-oxo-chromene-2-carboxylic acid (1 g, 4.46 mmol) in DCM (10 mL) at 0 °C was added oxalyl dichloride (765 µL, 8.92 mmol) followed by DMF (3 drops). The reaction mixture was heated at 40 °C for 40 min. The reaction mixture was concentrated under vacuum then dissolved in DCM (10 mL). 1-fluoro-cyclohexane

methanamine hydrochloride (1.1 g, 6.69 mmol) and DIPEA (2.3 mL, 13.4 mmol) were added and the reaction mixture was stirred at rt for 16 h. The reaction mixture was diluted with DCM (20 mL), washed with citric acid (3 × 20 mL), passed through a hydrophobic frit, and concentrated under vacuum. The product was triturated with Et_2O, purified by mass-directed preparative HPLC (0.1% formic acid, 5:95→95:5 MeCN: H_2O) then recrystallised from EtOH to afford 7-fluoro-*N*-((1-fluorocyclohexyl)methyl)-8-hydroxy-4-oxo-4H-chromene-2-carboxamide (147 mg, 10% yield) as a colourless solid. ^{1}H NMR (400 MHz, DMSO-d_6): δ 10.90 (s, 1H), 9.27 (t, J = 6.1 Hz, 1H), 7.51 (dd, J = 8.9, 5.7 Hz, 1H), 7.39 (dd, J = 10.5, 9.0 Hz, 1H), 6.85 (s, 1H), 3.62 (d, J = 6.4 Hz, 1H), 3.56 (d, J = 6.4 Hz, 1H), 1.79 – 1.71 (m, 2H), 1.61 – 1.48 (m, 7H), 1.31 – 1.29 (m, 1H). ^{13}C NMR (100 MHz, DMSO-d_6): δ 176.7, 159.1, 155.1, 153.7 (d, $^{1}J_{CF}$ = 246.4 Hz), 145.9 (d, $^{3}J_{CF}$ = 7.4 Hz), 134.8 (d, $^{2}J_{CF}$ = 14.6 Hz), 120.9, 114.6 (d, $^{2}J_{CF}$ = 16.8 Hz), 114.5 (d, $^{3}J_{CF}$ = 5.8 Hz), 110.9, 95.9 (d, $^{1}J_{CF}$ = 174.4 Hz), 46.5 (d, $^{2}J_{CF}$ = 22.8 Hz), 32.4 (d, $^{2}J_{CF}$ = 21.4 Hz), 24.6, 21.4 (d, $^{3}J_{CF}$ = 2.6 Hz). ^{19}F NMR (470 MHz, DMSO-d6) δ -126.60. HRMS (ES+): calcd. for $C_{17}H_{18}NO_4F_2$ $[M+H]^+$ 338.1204, found 338.1190. Purity: 99.95%

7-Fluoro-8-hydroxy-4-oxo-*N*-(tetrahydropyran-2-ylmethyl)chromene-2-carboxamide (DDD695)

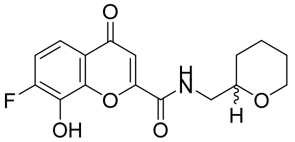

Synthesised according to procedure for DDD993 using tetrahydro-2*H*-pyran-2-methanamine hydrochloride (2 g, 13.4 mmol). The product was triturated with Et_2O, then the racemic mixture was separated by chiral SFC to afford 7-Fluoro-8-hydroxy-4-oxo-*N*-(tetrahydropyran-2-ylmethyl)chromene-2-carboxamide (412 mg, 14% yield) as a colourless solid. ^{1}H NMR (500 MHz, DMSO-d_6): δ 10.86 (s, 1H), 9.27 (t, J = 5.6 Hz, 1H), 7.51 (dd, J = 8.8, 5.6 Hz, 1H), 7.40 – 7.37 (m, 1H), 6.82 (s, 1H), 3.92 – 3.89 (m, 1H), 3.48 – 3.42 (m, 2H), 3.39 – 3.30 (m, 2H), 1.81 – 1.79 (m, 1H), 1.63 (d, J = 12.5 Hz, 1H), 1.50 – 1.43 (m, 3H), 1.26 – 1.19 (m, 1H). ^{13}C NMR (125 MHz, DMSO-d_6): δ 176.7, 158.5, 155.2, 153.7 (d, $^{1}J_{CF}$ = 246.5 Hz), 134.6, 120.9, 114.5 (d, $^{2}J_{CF}$ = 12.7 Hz), 114.4, 110.6, 75.7, 67.3, 44.3, 28.9, 25.5, 22.5. Note, one quaternary carbon was not detected. ^{19}F NMR (470 MHz, DMSO-d6) δ -126.62. HRMS (ES+): calcd. for $C_{16}H_{17}NO_5F$ $[M+H]^+$ 322.1091, found 322.1095. Purity: 99.83%

N-(cyclohexylmethyl)-6,7-difluoro-8-methoxy-4-oxo-4H-chromene-2-carboxamide

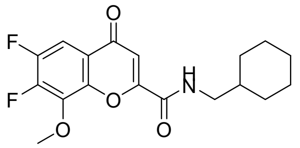

Synthesised according to procedure for DDD993 using 6,7-difluoro-8-methoxy-4-oxo-4H-chromene-2-carboxylic acid (750 mg, 2.93 mmol) and cyclohexylmethanamine (1.1 mL, 8.78 mmol). The product was purified by column chromatography (SiO_2, 0:100→100:0 EtOAc:heptanes) to afford *N*-(cyclohexylmethyl)-6,7-difluoro-8-methoxy-4-oxo-4H-chromene-2-carboxamide (499 mg, 49% yield) as an off-white solid. ^{1}H NMR (500 MHz, DMSO-d_6): δ 8.75 (t, J = 5.7 Hz, 1H), 7.68 (dd, J = 9.8, 8.4 Hz, 1H), 6.88 (s, 1H), 4.15 (s, 3H), 3.15 (t, J = 6.5 Hz, 2H), 1.72 – 1.68 (m, 4H), 1.63 – 1.57 (m, 2H), 1.21 – 1.16 (m, 3H), 0.98 – 0.91 (m, 2H). MS (ES+): *m/z* (%) 352 (99) $[M+H]^+$.

N-(cyclohexylmethyl)-6,7-difluoro-8-hydroxy-4-oxo-4H-chromene-2-carboxamide (DDD690)

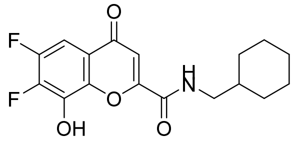

To a solution of 6,7-difluoro-8-methoxy-4-oxo-chromene-2-carboxylic acid (180 mg, 0.51 mmol) in DCM (10 mL) at -78 ºC was added tribromoborane (2.0 mL, 2.0 mmol) dropwise and the reaction mixture was stirred at 20 °C for 4 h. The reaction mixture was poured over ice and the resulting precipitate was collected by filtration and dried under vacuum to afford *N*-(cyclohexylmethyl)-6,7-difluoro-8-hydroxy-4-oxo-5,6-dihydro-4H-chromene-2-carboxamide (133 mg, 74% yield) as an off-white solid. ^{1}H NMR (500 MHz, DMSO-d_6): δ 11.36 (s, 1H), 9.07 (t, J = 5.9 Hz, 1H), 7.39 (dd, J = 9.9, 8.2 Hz, 1H), 6.83 (s, 1H), 3.21 (t, J = 6.5 Hz, 2H), 1.75 – 1.69 (m, 4H), 1.64 – 1.56 (m, 2H), 1.23 – 1.14 (m, 3H), 1.00 – 0.94 (m, 2H). ^{13}C NMR (125 MHz, CD_3OD): δ 178.9, 161.1, 157.4, 151.1 (d, $^1J_{CF}$ = 260.4 Hz), 145.1 (d, $^1J_{CF}$ = 250.9 Hz), 138.8, 133.9, 121.1, 111.5, 101.3 (d, $^2J_{CF}$ = 20.3 Hz), 47.4, 39.2, 32.0, 27.5, 27.0. ^{19}F NMR (470 MHz, DMSO-d6) δ -137.75 (d, J = 22.4 Hz), -150.51 (d, J = 22.2 Hz). HRMS (ES+): calcd. for $C_{17}H_{18}NO_4F_2$ $[M+H]^+$ 338.1204, found 338.1202. Purity: 99.4%

6-methoxy-4-oxo-4H-chromene-2-carboxylic acid

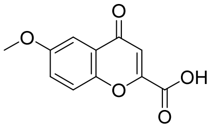

To a solution of sodium ethoxide in ethanol (59.6 mL, 151 mmol, 21%) was added a solution of 1-(2-hydroxy-5-methoxyphenyl)ethan-1-one (5 g, 30.1 mmol) in diethyl oxalate (16.3 mL, 120 mmol) and the reaction mixture was heated at 80 °C for 2 h. The reaction mixture was cooled and diluted with ethanol (20 mL). Conc. HCl (18.3 mL, 602 mmol) was added, and the reaction mixture was heated at 90 °C for 3 h. The reaction mixture was cooled, filtered, and concentrated under vacuum. The residue was dissolved in ethanol (20 mL) and water (20 mL). Sodium hydroxide (4.81 g, 120 mmol) was added, and the reaction mixture was stirred at rt for 16 h. The reaction mixture was acidified to pH1 with 2M HCl and extracted with EtOAc (3 × 100 mL). The resulting precipitate was collected by filtration and dried under vacuum to afford 6-methoxy-4-oxo-chromene-2-carboxylic acid (5.33 g, 80% yield) as a yellow solid. ^{1}H-NMR (500 MHz, DMSO-d_6): δ 7.70 (d, J = 9.2 Hz, 1H), 7.46 (dd, J = 9.2, 3.2 Hz, 1H), 7.40 (d, J = 3.2 Hz, 1H), 6.89 (s, 1H), 3.88 (s, 3H). Note, exchangeable proton was not detected. MS (ES+): *m/z* (%) 221 (97) $[M+H]^+$.

***N*-((1-hydroxycyclohexyl)methyl)-6-methoxy-4-oxo-4H-chromene-2-carboxamide (DDD583)**

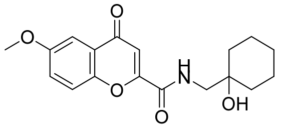

To a solution of 6-methoxy-4-oxo-chromene-2-carboxylic acid (2.94 g, 13.4 mmol), HATU (5.58 g, 14.7 mmol), and triethylamine (4.65 mL, 33.4 mmol) in DMF (10 mL) was added 1-aminomethyl-1-cyclohexanol hydrochloride (2.21 g, 13.4 mmol) and the reaction mixture was stirred at 20 °C for 60 h. The reaction mixture was concentrated under vacuum then diluted with EtOAc (10 mL) and washed with water (3 × 10 mL). The resulting precipitate was collected by filtration and dried under vacuum. The product was recrystallised from EtOH then triturated with TBME (2 mL) to afford *N*-((1-hydroxycyclohexyl)methyl)-6-methoxy-4-oxo-4H-chromene-2-carboxamide (968 mg, 21% yield) as an off-white solid. ^{1}H-NMR (500 MHz, DMSO-d_6): δ 8.66 (t, J = 6.1 Hz, 1H), 7.73 (d, J = 9.2 Hz, 1H), 7.49 (dd, J = 9.2, 3.2 Hz, 1H), 7.42 (d, J = 3.2 Hz, 1H), 6.83 (s, 1H), 4.37 (br. s, 1H), 3.88 (s, 3H), 3.31 – 3.29 (m, 2H), 1.60

– 1.53 (m, 2H), 1.48 – 1.34 (m, 7H), 1.25 – 1.18 (m, 1H). ^{13}C-NMR (125 MHz, DMSO-d_6): δ 176.9, 159.4, 156.9, 155.5, 149.7, 124.4, 124.0, 120.4, 109.6, 104.6, 70.2, 55.7, 49.4, 34.9, 25.3, 21.4. HRMS (ES+): calcd. for $C_{18}H_{22}NO_5$ $[M+H]^+$ 332.1498, found 332.1506. Purity: 99.98%

1-(2-hydroxy-5-methoxy-3-nitrophenyl)ethan-1-one

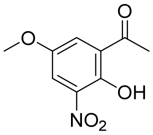

Fuming HNO_3 (8.75 g, 139 mmol) in AcOH (25 mL) was added dropwise to a solution of 1-(2-hydroxy-5-methoxyphenyl)ethanone (22.5 g, 135 mmol) in AcOH (225 mL) at 10 °C, and the reaction mixture was stirred at 25 °C for 2 h. The reaction mixture was poured over ice and the resulting precipitate was filtered, washed with water, azeotroped with toluene and dried under vacuum to afford 1-(2-hydroxy-5-methoxy-3-nitrophenyl)ethan-1-one (47 g, 82% yield) as a yellow solid. ^{1}H NMR (400 MHz, DMSO-d_6): δ 12.24 (s, 1H), 7.80 (d, J = 3.2 Hz, 1H), 7.75 (d, J = 3.2 Hz, 1H), 3.84 (s, 3H), 2.71 (s, 3H). MS (ES+): m/z (%) 212 (100) $[M+H]^+$.

ethyl 6-methoxy-8-nitro-4-oxo-4H-chromene-2-carboxylate

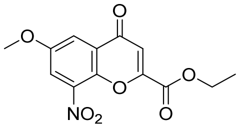

Na (2.18 g, 94.7 mmol) was added to EtOH (20 mL) at 0 °C and stirred for 30 min then 1-(2-hydroxy-5-methoxy-3-nitrophenyl)ethanone (2 g, 9.47 mmol) was added and the reaction mixture was stirred at 25 °C for 30 min. Diethyl oxalate (6.47 mL, 47.4 mmol) was added and the reaction mixture was heated at 80 °C for 2 h. The reaction mixture was diluted with 1M HCl, extracted with EtOAc (3 × 20 mL), dried over $MgSO_4$ and concentrated under vacuum. The residue was dissolved in EtOH (20 mL) and conc. HCl (3.2 mL) was added. The reaction mixture was heated at 80 °C for 12 h then concentrated under vacuum and triturated with cold EtOH (20 mL) to afford ethyl 6-methoxy-8-nitro-4-oxo-4H-chromene-2-carboxylate (2 g, 98% yield) as a yellow solid. ^{1}H NMR (400 MHz, DMSO-d_6): δ 8.18 (d, J = 3.2 Hz, 1H), 7.74 (d, J = 2.8 Hz, 1H), 7.04 (s, 1H), 4.50 - 4.32 (m, 2H), 3.96 (s, 3H), 1.43 - 1.25 (t, J = 7.2 Hz, 3H). MS (ES+): m/z (%) 294 (98) $[M+H]^+$.

6-methoxy-8-nitro-4-oxo-4H-chromene-2-carboxylic acid

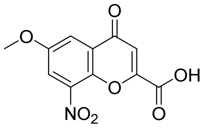

To a mixture of ethyl 6-methoxy-8-nitro-4-oxo-4*H*-chromene-2- carboxylate (1 g, 3.41 mmol) in AcOH (20 mL) at 0 °C under N_2 was added conc. HCl (5 mL, 50.4 mmol). The reaction mixture was heated at 85 °C for 1 h then quenched with sat. NH_4Cl (100 mL) and extracted with EtOAc (3 × 100 mL). The reaction mixture was diluted with water (50 mL), filtered, dried under vacuum, and azeotroped with toluene to afford 6-methoxy-8-nitro-4-oxo-4H-chromene-2-carboxylic acid (0.9 g, 99% yield) as an off-white solid. ^1H NMR (400 MHz, DMSO-d_6): δ 8.16 (d, J = 3.2 Hz, 1H), 7.73 (d, J = 3.2 Hz, 1H), 6.99 (s, 1H), 3.95 (s, 3H). Note, exchangeable proton was not detected. MS (ES+): *m/z* (%) 266 (98) $[M+H]^+$.

(*S*)-*N*-(1-cyclohexylethyl)-6-methoxy-8-nitro-4-oxo-4H-chromene-2-carboxamide

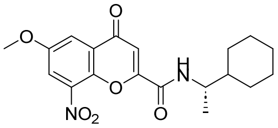

To a solution of 6-methoxy-8-nitro-4-oxo-4*H*-chromene-2-carboxylic acid (25 g, 94.3 mmol) in DCM (250 mL) was added $(COCl)_2$ (16.5 mL, 188 mmol) at 0 °C, followed by DMF (72.5 µL, 0.94 mmol). The reaction mixture was heated at 40 °C for 1 h. The reaction mixture was concentrated under vacuum then dissolved in DCM (250 mL). A solution of (*S*)-1-cyclohexylethanamine (19.7 mL, 132 mmol) and DIPEA (46.1 mL, 264 mmol) in DCM (250 mL) was added at 0 °C, and the reaction mixture was stirred at 25 °C for 1 h. The reaction mixture was diluted with water (500 mL) and extracted with DCM (3 × 200 mL). The combined organics were washed with brine (500 mL), dried over Na_2SO_4, filtered and concentrated under vacuum. The product was purified by column chromatography (SiO_2, 10:90→50:50 EtOAc:petroleum ether) to afford (*S*)-*N*-(1-cyclohexylethyl)-6-methoxy-8-nitro-4-oxo-4H-chromene-2-carboxamide (30 g, 91% yield) as a white solid. ^1H NMR (400 MHz, DMSO-d_6): δ 8.20 - 8.11 (m, 2H), 7.76 (d, J = 3.2 Hz, 1H), 6.88 (s, 1H), 3.96 (s, 3H), 3.88 - 3.78 (m, 1H), 1.81 - 1.56 (m, 6H), 1.50 - 1.35 (m, 1H), 1.30 - 1.19 (m, 2H), 1.13 (d, J = 12.0 Hz, 3H), 1.06 - 0.94 (m, 2H). MS (ES+): *m/z* (%) 375 (97) $[M+H]^+$.

(*S*)-8-amino-*N*-(1-cyclohexylethyl)-6-methoxy-4-oxo-4H-chromene-2-carboxamide

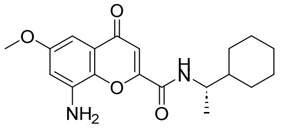

To a mixture of (*S*)-*N*-(1-cyclohexylethyl)-6-methoxy-8-nitro-4-oxo-4*H*-chromene-2-carboxamide (15 g, 40.1 mmol) in EtOH (120 mL) and H_2O (30 mL) was added Fe (6.71 g, 120 mmol) and NH_4Cl (6.43 g, 120 mmol). The reaction mixture was heated at 100 °C for 16 h. The reaction mixture was filtered, and the filtrate was diluted with water (300 mL) and extracted with ethyl acetate (3 × 200 mL). The combined organics were washed with brine (300 mL), dried over Na_2SO_4, filtered and concentrated under vacuum. The product was purified by column chromatography (SiO_2, 15:85→30:70 EtOAc:petroleum ether) to afford (*S*)-8-amino-*N*-(1-cyclohexylethyl)-6-methoxy-4-oxo-4H-chromene-2-carboxamide (11.4 g, 81% yield) as a yellow solid. ^1H NMR (400 MHz, DMSO-d_6): δ 8.73 (d, J = 8.8 Hz, 1H), 6.79 (s, 1H), 6.68 - 6.56 (m, 2H), 6.24 (s, 2H), 3.55 - 3.85 (m, 1H), 3.76 (s, 3H), 1.83 - 1.64 (m, 4H), 1.63 - 1.54 (m, 1H), 1.53 - 1.40 (m, 1H), 1.25 - 1.05 (m, 6H), 1.01 - 0.85 (m, 2H). MS (ES+): *m/z* (%) 345 (99) $[M+H]^+$.

(*S*)-*N*-(1-cyclohexylethyl)-6-methoxy-4-oxo-8-((2-(pyrrolidin-1-yl)ethyl)sulfonamido)-4H-chromene-2-carboxamide (DDD582)

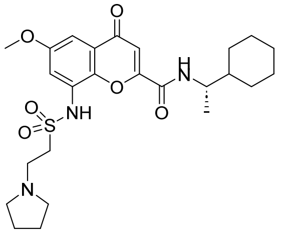

To a mixture of (*S*)-8-amino-*N*-(1-cyclohexylethyl)-6-methoxy-4-oxo-4*H*-chromene-2-carboxamide (1.0 g, 2.90 mmol) and pyridine (1.17 mL, 14.5 mmol) in DCE (30 mL) was added 2-chloroethanesulfonyl chloride (1.5 mL, 14.5 mmol). The reaction mixture was heated at 45 °C for 18.5 h, then pyrrolidine (4.8 mL, 58.1 mmol) was added, and the reaction mixture was heated at 45 °C for 2.5 h. The reaction mixture was diluted with water (50 mL) and extracted with DCM (3 × 30 mL). The combined organics were washed with brine (100 mL), dried over Na_2SO_4, filtered, and concentrated under vacuum. The product was purified by preparative HPLC (0.1% NH_4OH, 10:90→40:60 MeCN: H_2O), then lyophilized. The residue was diluted with DCM (15 mL) and adjusted to pH ~ 8 with sat. $NaHCO_3$, then extracted with

DCM (3 × 15 mL), washed with brine (15 mL), dried over Na_2SO_4, filtered and concentrated under vacuum to afford (*S*)-*N*-(1-cyclohexylethyl)-6-methoxy-4-oxo-8-((2-(pyrrolidin-1-yl)ethyl)sulfonamido)-4H-chromene-2-carboxamide (461 mg, 31% yield) as a yellow solid. ^{1}H-NMR (500 MHz, DMSO-d_6): δ 8.85 (d, *J* = 8.7 Hz, 1H), 7.38 (d, *J* = 2.8 Hz, 1H), 7.18 (d, *J* = 2.7 Hz, 1H), 6.83 (s, 1H), 3.88 – 3.83 (m, 4H), 3.29 (t, *J* = 7.2 Hz, 2H), 2.81 (t, *J* = 7.2 Hz, 2H), 2.38 – 2.36 (m, 4H), 1.78 – 1.77 (m, 2H), 1.74 – 1.69 (m, 2H), 1.63 – 1.61 (m, 1H), 1.56 – 1.53 (m, 4H), 1.50 – 1.44 (m, 1H), 1.24 – 1.11 (m, 7H), 1.04 – 0.93 (m, 2H). ^{13}C-NMR (125 MHz, DMSO-d_6): δ 176.7, 158.1, 156.5, 155.3, 143.0, 130.1, 124.9, 116.8, 110.0, 100.2, 55.7, 53.1, 50.8, 49.9, 49.2, 42.2, 29.2, 28.8, 25.8, 25.6, 22.9, 17.5. HRMS (ES+): calcd. for $C_{25}H_{36}N_3O_6S$ $[M+H]^+$ 506.2325, found 506.2328. Purity: 99.89%

X-ray crystallography

Structures of *Cp*KRS were obtained using a similar method to that described previously.*(8)* Crystals were grown using hanging drop vapour diffusion, with the protein incubated with 2 mM lysine prior to crystallisation. Crystals were transferred into drops consisting of 1 µL of reservoir solution (25% PEG 3350, 0.2 M lithium sulfate and 0.1 M tris pH 7.8) mixed with 1 µL of protein buffer (25 mM HEPES, 0.5 M NaCl, 5 % w/v glycerol, 2 mM TCEP, pH 7.0) supplemented with 10 mM ligand, from a 200 mM stock in DMSO. Crystals were soaked for one hour prior to flash freezing in liquid nitrogen, cryo protected using reservoir supplemented with 33% glycerol.

Diffraction data for *Cp*KRS:DDD01529508 were collected at beamline I03 at Diamond Light Source, integrated using XDS*(41)* and xia2,*(42)* and scaled and merged using Aimless.*(43)* Data for *Cp*KRS:DDD01867489 were collected at beamline ID2301 at the European Synchrotron Radiation Facility, integrated using XDS and scaled and merged using Aimless.

Structures for both compounds were solved by molecular replacement, using the previously determined structure of *Cp*KRS (PDB: 5elo) as a search model in Phaser.*(44)* Manual model building was performed using Coot,*(45)* and the structure refined using Refmac,*(46)* incorporated into the CCP4 suite of software.*(47)* The ligand dictionary was prepared using AceDRG,*(48)* and model quality assessed using Molprobity.*(49)* Data collection and refinement statistics are given in Table S6.

Supplementary figures

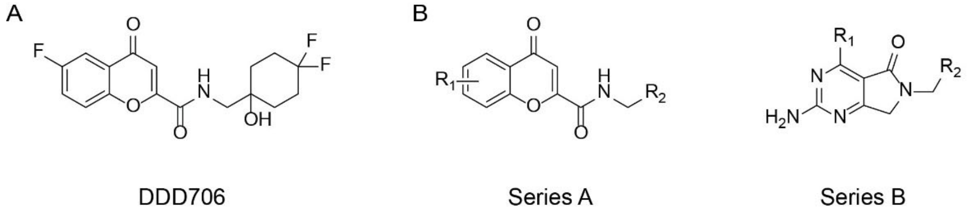

Figure S1. Chemical structures of lead compounds and series.

(A) Early lead *Cp*KRS inhibitor, DDD01510706, "DDD706", also referred to as compound 5 in cited publication.*(8)* **(B)** Generic structures of two series of *Cp*KRS inhibitors developed through medicinal chemistry optimization.

DDD212
EC_{50} 0.17 µM

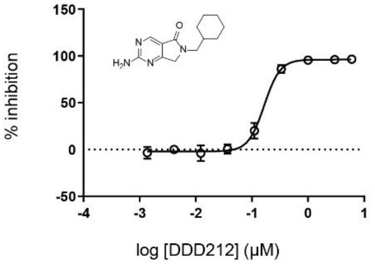

DDD993
EC_{50} 0.10 µM

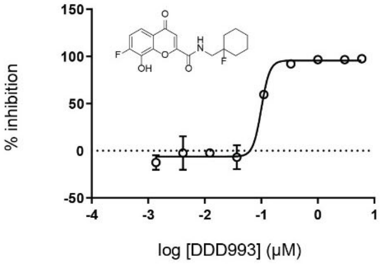

DDD714
EC_{50} 0.03 µM

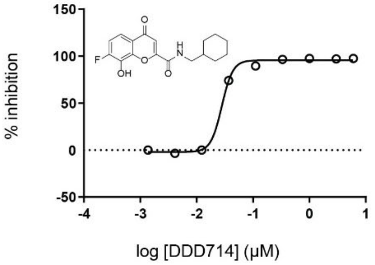

DDD690
EC_{50} 0.19 µM

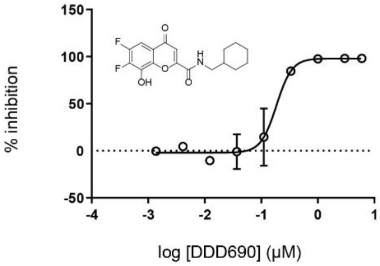

DDD909
EC_{50} 0.07 µM

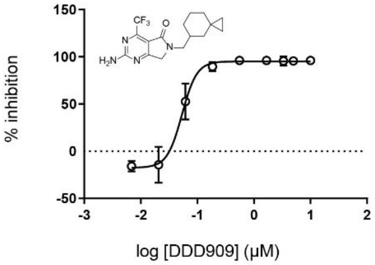

DDD489
EC_{50} 0.04 µM

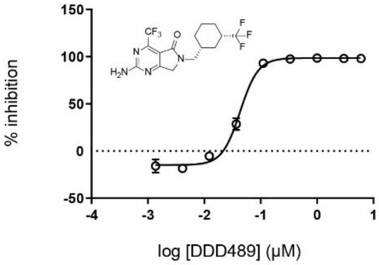

DDD352
EC_{50} 0.02 µM

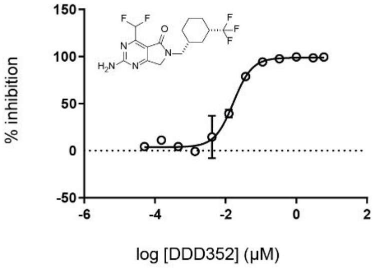

DDD478
EC_{50} 0.02 µM

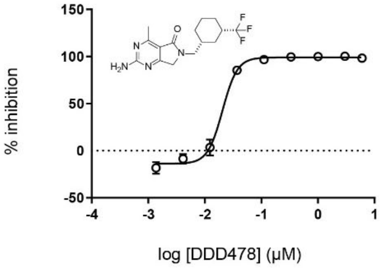

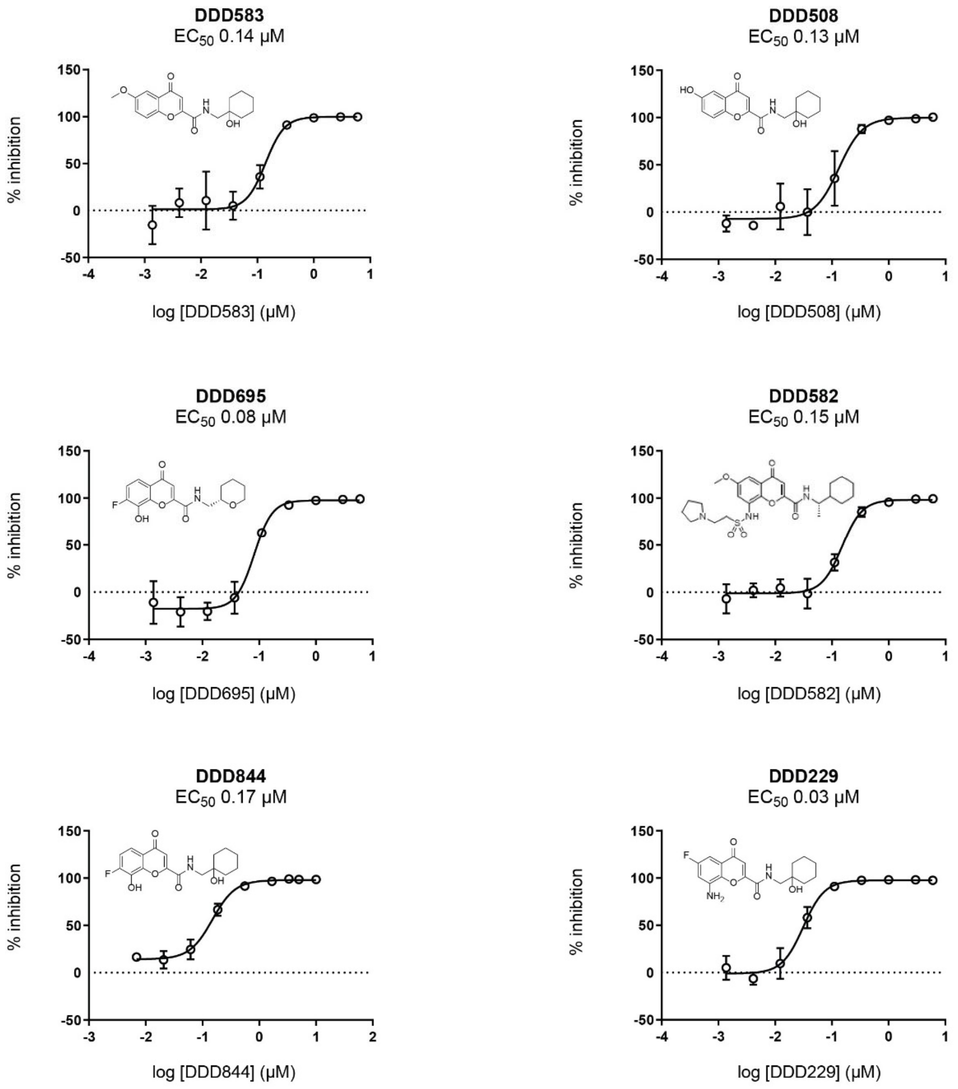

Figure S2. Potent tool compounds were selected with *C. parvum* EC_{50} < 0.2 µM.

In vitro dose response curves for parasite growth inhibition were determined for all fourteen tool compounds; EC_{50} values and chemical structure reported. HCT-8 cells were infected with wild type *C. parvum*, compound was added at the time of infection, and inhibition of parasite growth was measured by immunofluorescence staining assay (VVL-FITC to detect *Cryptosporidium* and DAPI to quantify HCT-8 cells) and quantified using high-throughput imaging and analysis. Four parameter logistic non-linear regression plotted (GraphPad). Mean ± SD (six technical replicates at each concentration); representative graph of two biological replicates shown.

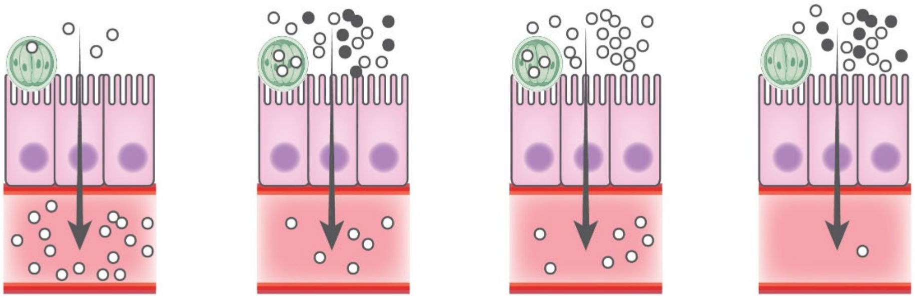

Figure S3. Hypothesized compound absorption by BCS class.

Illustration of effect of solubility and permeability on absorption of drug molecule into systemic circulation and resulting concentration that remains in the small intestine and is available to accumulate within C*ryptosporidium* parasitophorous vacuole. Black circles represent solid drug molecules and white circles represent solubilized drug molecules.

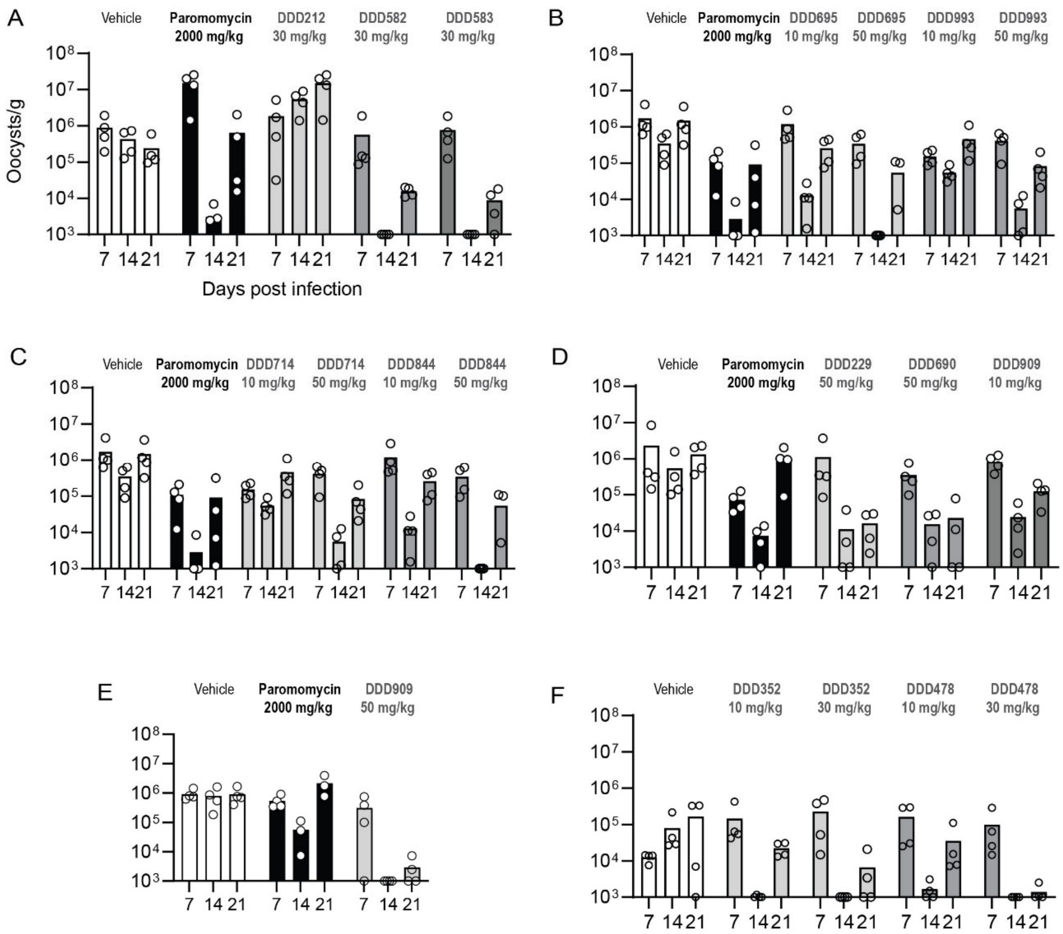

Figure S4. Efficacy of tool compounds in NOD SCID Gamma mouse model.

NOD SCID mice (4 mice per cage) were infected with wild type *C. parvum*. Starting day 7 PI, mice were treated with vehicle (white), positive control (paromomycin, black), or experimental compounds (gray) via oral gavage. Tool compounds were administered twice daily for 7 days (ending on day 13 PI). Fecal samples were collected at day 7, 14, and 21 PI from individual animals. Parasite shedding was measured using qPCR at the individual animal level and reported as oocysts/gram fecal material. Bar height indicates mean for each treatment group (4 mice per cage), data points indicate individual animals (mean of three technical replicates plotted). (**A-F**) Data is grouped by independent biological experiment with corresponding controls. Compounds described here failed to reduce parasite shedding to sufficient levels at day 14 PI or they failed to prevent parasite recrudescence at day 21 PI.

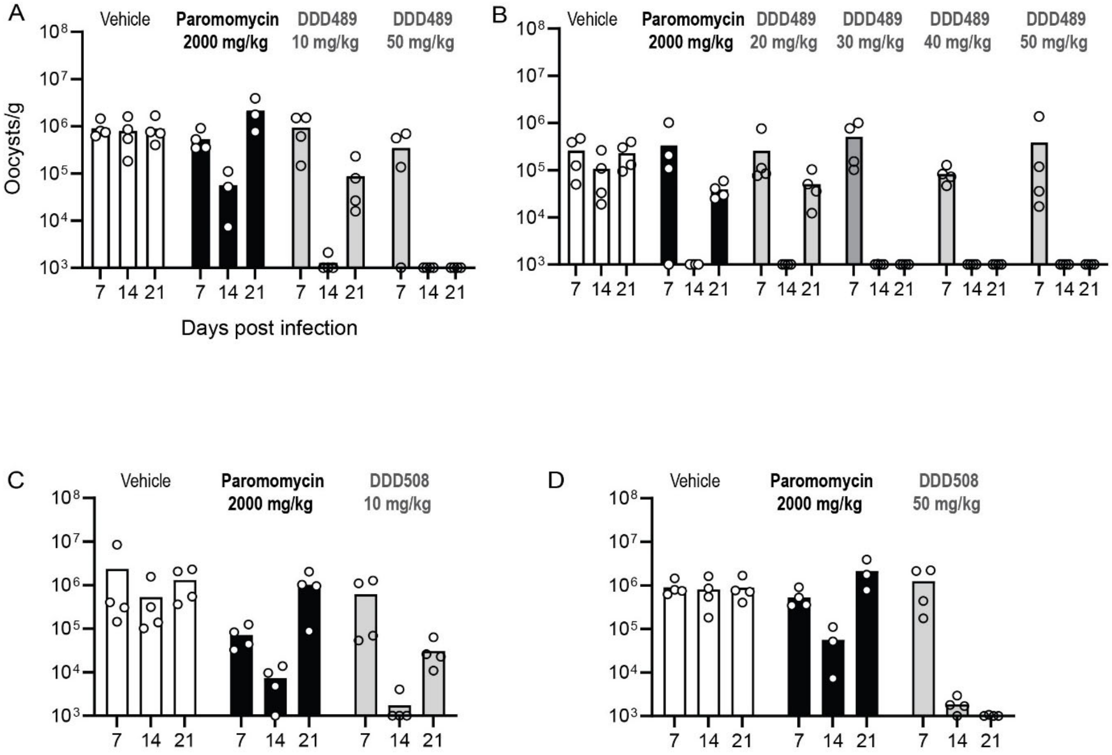

Figure S5. Efficacy of DDD489 and DDD508 in NOD SCID Gamma mice when administered at other doses.

NOD SCID mice (4 mice per cage) were infected with wild type *C. parvum* as described in Fig. 1C. Starting day 7 post infection, mice were treated with vehicle (white), positive control (paromomycin, black), or DDD489 (gray, **A** and **B**) or DDD508 (gray, **D** and **E**) via oral gavage. Late lead compounds were administered twice daily for 7 days (ending on day 13 PI). Fecal samples were collected at day 7, 14, and 21 PI from individual animals. Fecal samples were collected from individual mice and analyzed by qPCR to quantify parasite shedding (oocysts/g). Bar height indicates mean for each treatment group (4 mice per cage), data points indicate individual animals (mean of three technical replicates plotted). **(A)** Treatment with 10 mg/kg DDD489 reduces parasite shedding to the level of detection once treatment has finished but does not prevent recrudescence at day 21 PI. Treatment with 50 mg/kg DDD489 prevents recrudescence at day 21 PI. **(B)** Dose response experiment identifies 30 mg/kg DDD489 as the minimum efficacious dose required to reduce parasite shedding and prevent parasite recrudescence (data also presented in Fig. 2A). **(C)** Treatment with 10 mg/kg DDD508 reduces parasite shedding to the level of detection at day 14 PI, **(D)** but 30 mg/kg (Fig. 2B) or 50 mg/kg is required to also prevent parasite recrudescence.

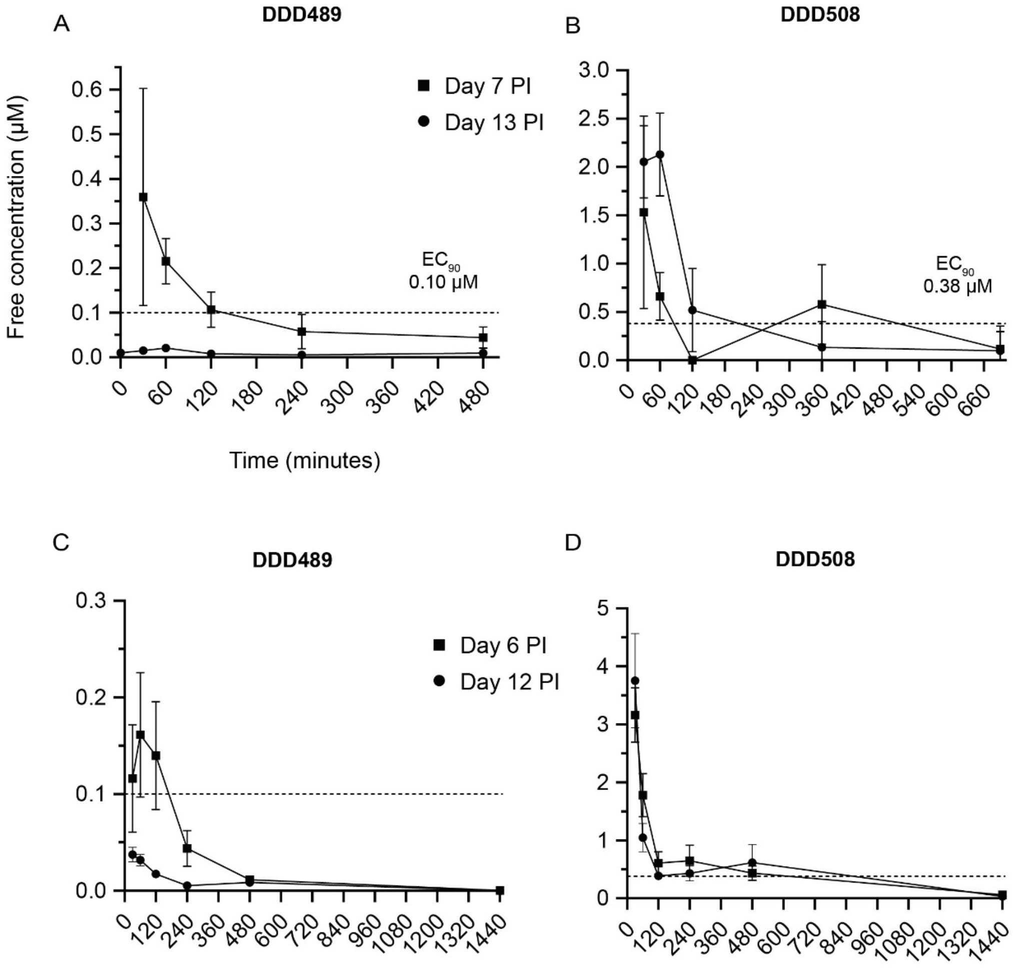

Figure S6. Blood concentration time profiles for late lead compounds in infected mice.

Mean free blood concentration time profiles following oral administration of late lead compounds to NOD SCID Gamma (**A and B**) and IFN-Gamma KO (**C and D**) mice twice daily for 7 days as described in Fig. 1C and Fig. 4A) respectively. Data are mean ± SD (3 mice per group). Blood samples were collected on the first day (squares) and last day (circles) of compound dosing (Day 7 and 13 PI for NOD SCID Gamma, Day 6 and 13 for IFN-Gamma KO). Total drug concentrations were corrected for fraction unbound (fu) in PPB assay (DDD489 fu: 0.16; DDD508 fu: 0.6) assuming a blood to plasma ratio of 1. Dotted lines indicate *C. parvum* EC_{90}, calculated from the average EC_{50} and hill slope **(**DDD489 EC_{50} = 0.043 µM, hill slope 2.716; DDD508 EC_{50} = 0.132 µM, hill slope 2.076). **(A)** DDD489 (30 mg/kg) was administered to NOD SCID Gamma mice. **(B)** DDD508 (10 mg/kg) was administered to NOD SCID Gamma mice. Blood concentrations were scaled to 30 mg/kg for comparison, assuming dose linearity. **(C)** DDD489 (50 mg/kg) was administered to IFN-Gamma KO mice. **(D)** DDD508 (50 mg/kg) was administered to IFN-Gamma KO mice.

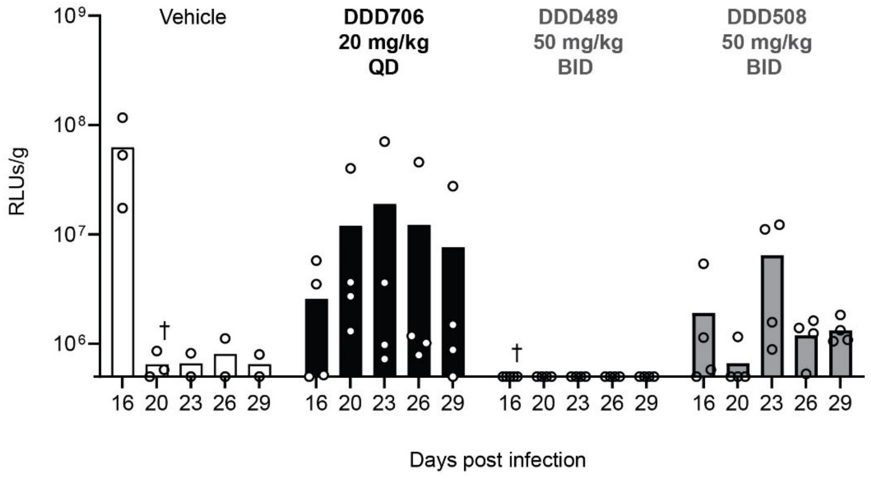

Figure S7. DDD489 prevents parasite recrudescence in the acute cryptosporidiosis model (IFN-Gamma KO mice).

Mice were treated with vehicle (white), DDD706 (positive control, black), DDD489 (gray), DDD508 (gray) by oral gavage (as described in **Fig. 4A**). Fecal samples were collected days 16-29 PI to measure parasite recrudescence. Fecal samples collected from individual animals (pooled samples described in **Fig. 4B**) and parasite shedding quantified via NanoLuciferase assay (relative luminesce units, RLUs/g). Bar height indicates average for each treatment group (5 mice per cage), data points indicate individual animals (average of three technical replicates plotted). †Mice were culled due to weight loss (1 vehicle-treated mouse at day 20 PI; 1 DDD489-treated mouse at day 16 PI).

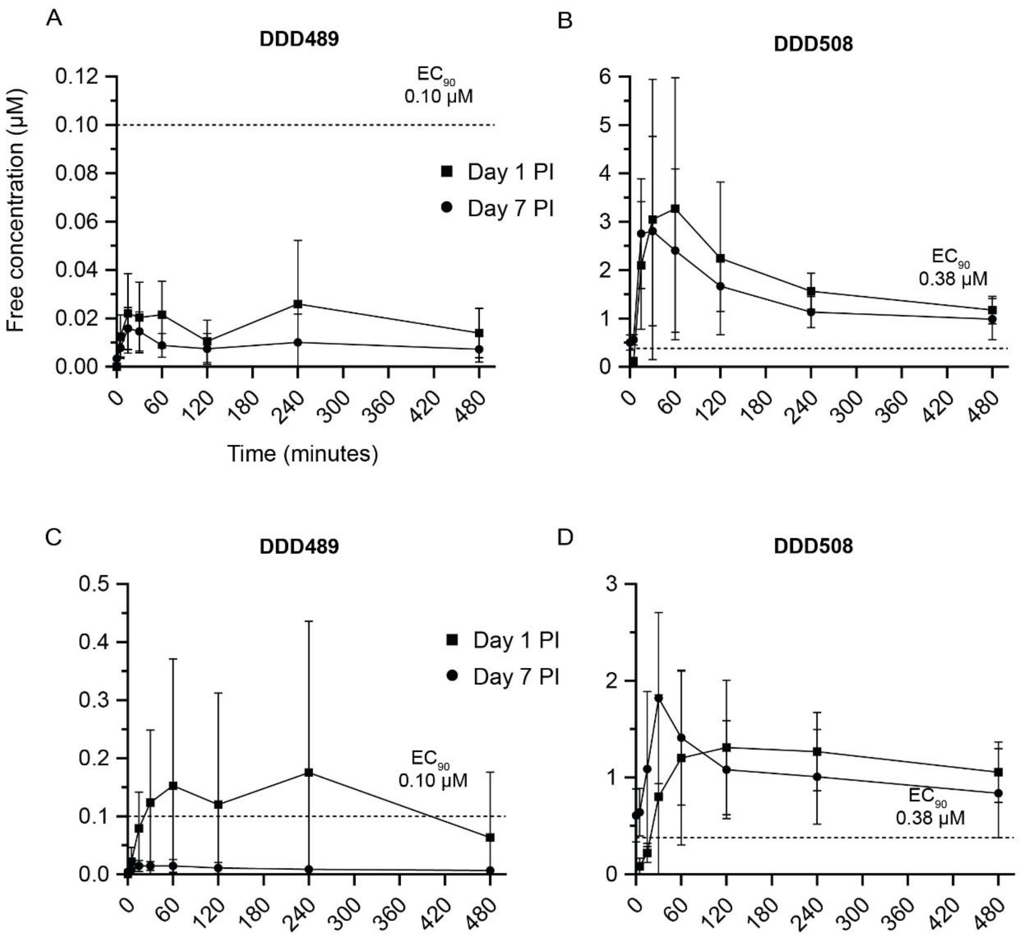

Figure S8. Plasma concentration time profiles for late lead compounds in uninfected and infected calves.

Mean free plasma concentration time profiles following oral administration of late lead compounds at 15 mg/kg BID for 7 days to uninfected calves (**A and B**, 4 calves per group) and to infected calves as described in Fig. 5A (**C and D**, 7 calves per group). Data are mean ± SD. Blood samples were collected on the first day (squares) and last day (circles) of compound dosing (Day 1 and 7 PI) and processed to plasma. Total drug concentrations were corrected for fraction unbound (fu) in PPB assay (DDD489 fu: 0.26; DDD508 fu: 0.53). Dotted lines indicate *C. parvum* EC_{90}, calculated from the average EC_{50} and hill slope (DDD489 EC_{50} = 0.043 µM, hill slope 2.716; DDD508 EC_{50} = 0.132 µM, hill slope 2.076).

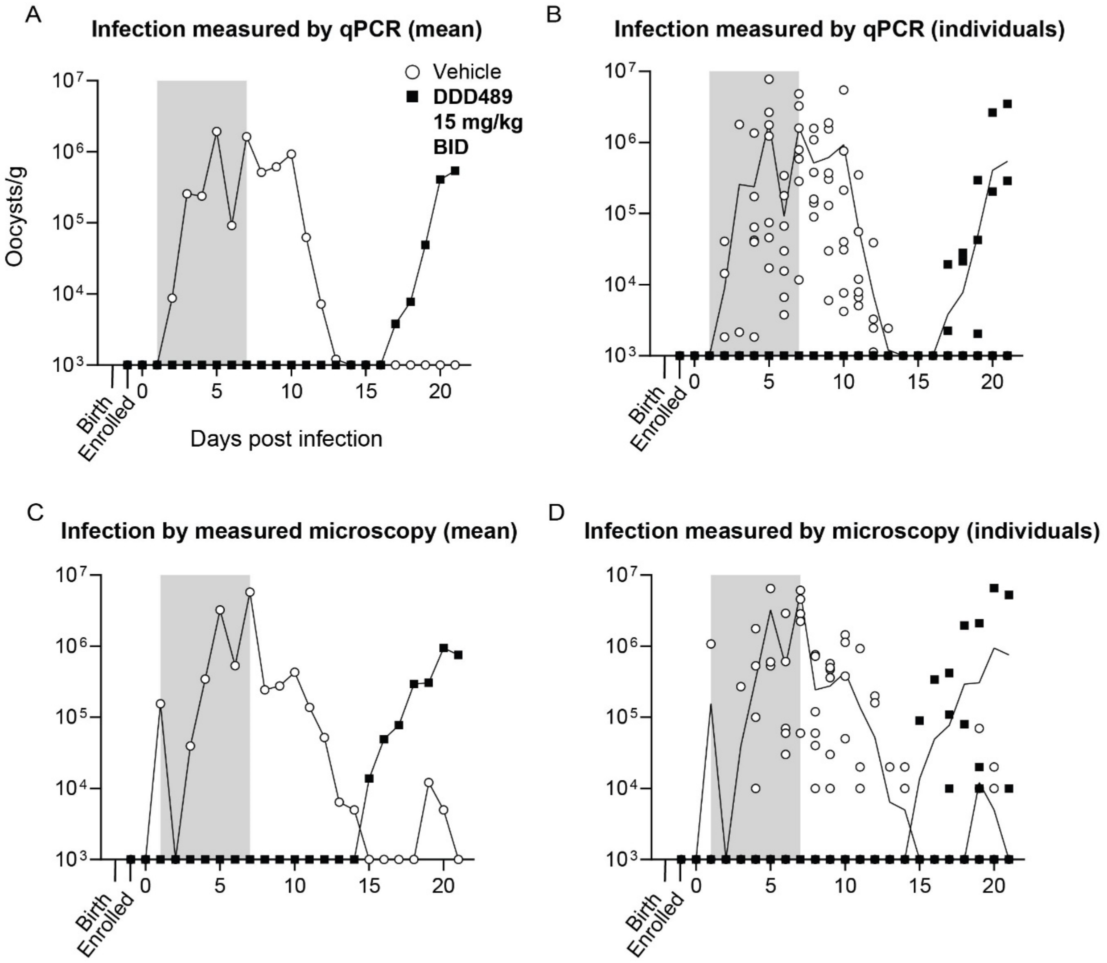

Figure S9. Reduction in parasite shedding from DDD489 calf efficacy study graphed by group mean or individual animal.

Parasite shedding of vehicle-treated group (white), or DDD489-treated group (black) was quantified by qPCR and was plotted using the mean for each group (**A**, reported in Fig. 5B) or individual animal values (**B**). Parasite shedding was also quantified by immunofluorescence microscopy (using commercial detection reagents, see Methods) and was plotted as the mean for each group (**C**) or individual animal values (**D**). Similar infection patterns were observed using either qPCR or microscopy-based methods.

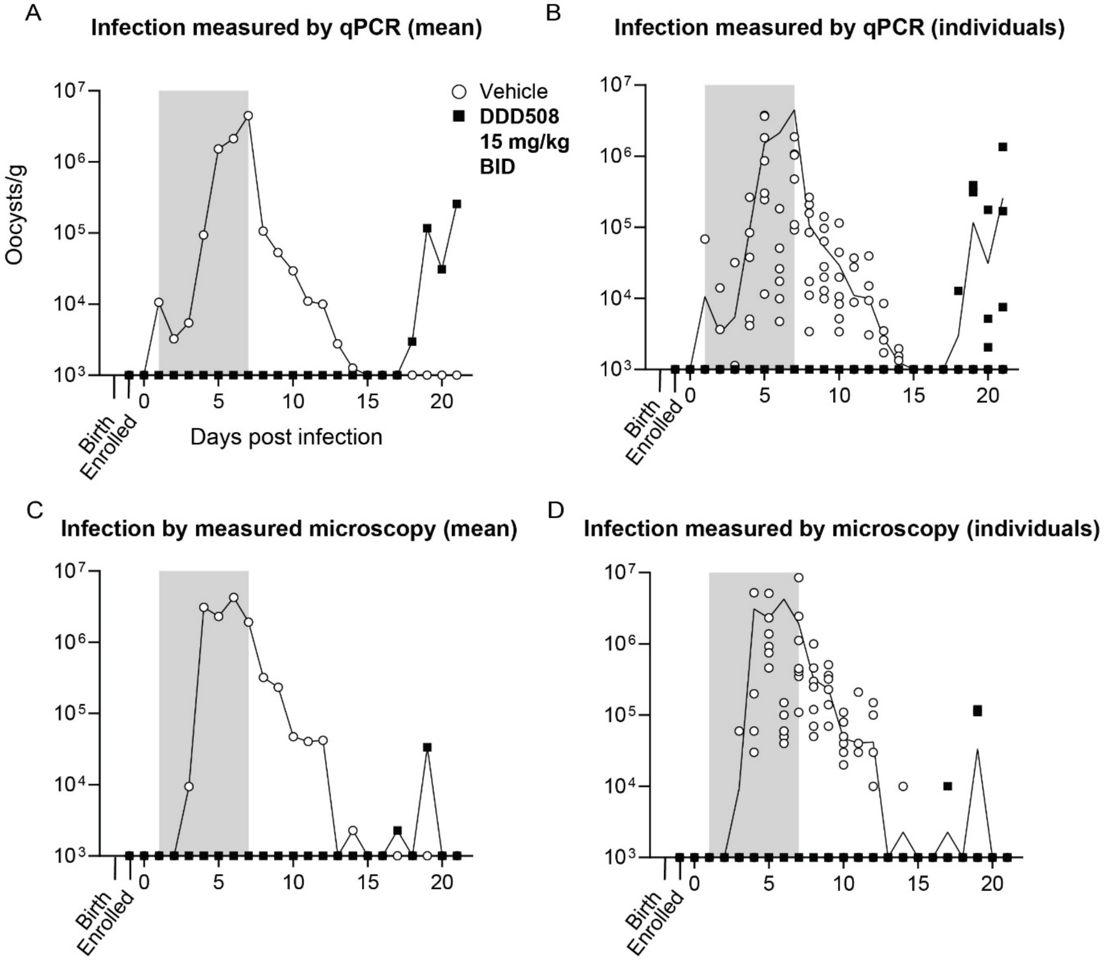

Figure S10. Reduction in parasite shedding from DDD508 calf efficacy study graphed by group mean or individual animal.

Parasite shedding of vehicle-treated group (white), or DDD508-treated group (black) was quantified by qPCR and was plotted using the mean for each group (**A,** reported in Fig. 5C) or individual animal values (**B**). Parasite shedding was also quantified by immunofluorescence microscopy (using commercial detection reagents, see Methods) and was plotted as the mean for each group (**C**) or individual animal values (**D**). Similar infection patterns were observed using either qPCR or microscopy-based methods.

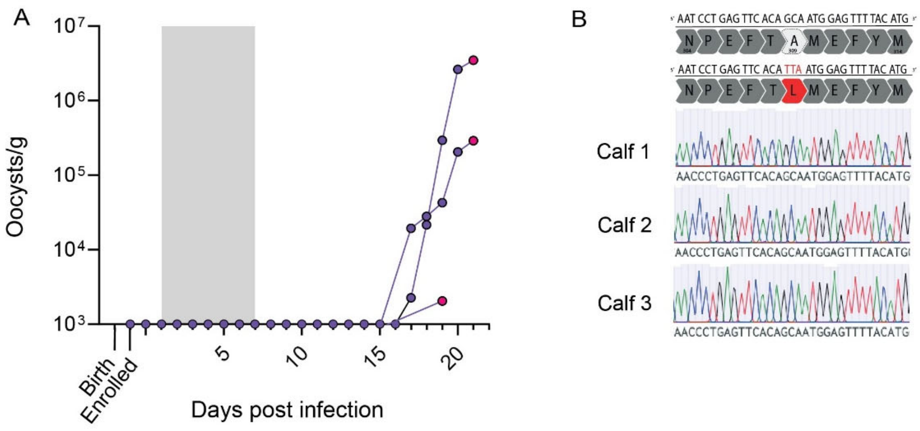

Figure S11. Recrudescence of *Cryptosporidium* shedding in treated calves is not due to mutation of the target gene.

(**A**) Parasite shedding of individual animals was quantified by qPCR. Of the seven calves treated with DDD489 (purple), three calves shed *Cryptosporidium* starting day 15 or 16 PI, almost two weeks after treatment concluded (treatment period indicated in gray). Fecal samples with the highest level of infection from each animal (samples indicated in pink) was used for DNA was extraction. The *Cp*KRS locus was amplified by PCR. (**B**) DNA sequencing confirms there no are mutations at the *Cp*KRS locus shed from these calves. In particular, the mutation known to generate resistance to KRS inhibitors (alanine 309 to leucine) was not present in any of the three samples analyzed.

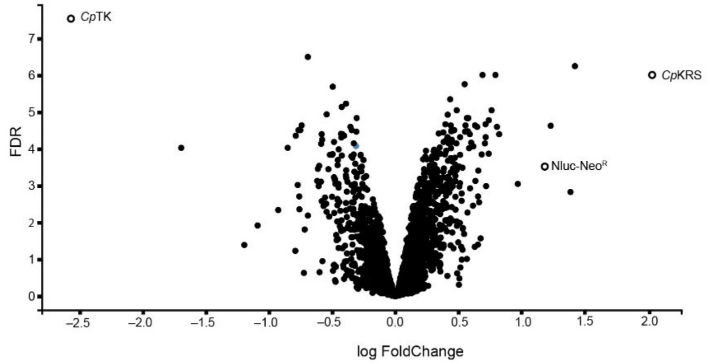

Figure S12. Quantitative proteomics confirms *Cp*KRS is over-expressed in genetically modified strain.

C. parvum was genetically modified to over-express *Cp*KRS. The *Cp*TK locus was replaced with a cassette containing *Cp*KRS and the dual reporter-selection marker, Nluc-Neo^{R}, driven by the *CpEnolase* promoter as previously described.*(12)* Quantitative proteomics analysis confirms over-expression of *Cp*KRS and similar significantly increased expression of Nluc-Neo^{R}.

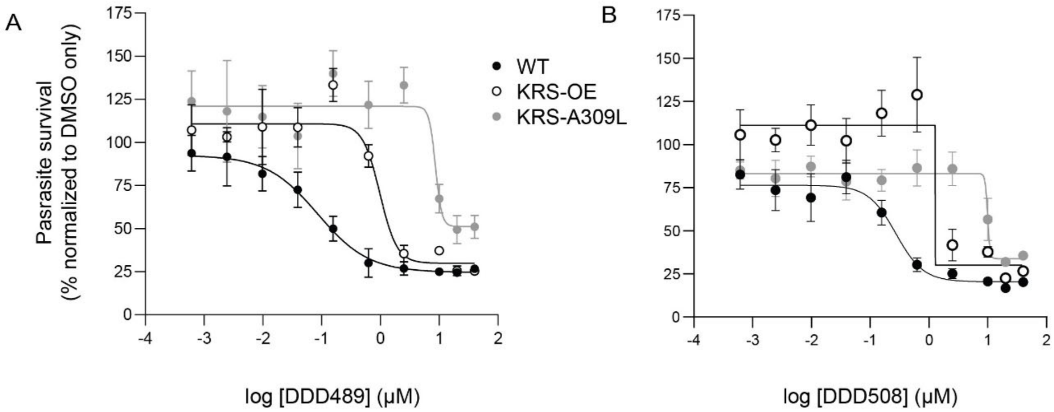

Figure S13. Genetic validation confirms that late lead compounds are on-target.

Overexpression of *Cp*KRS (KRS-OE) or mutation of *Cp*KRS with an A309L substitution (KRS-A309L) render parasites less susceptible to treatment with DDD489 (**A**) and DDD508 (**B**) compared to wild type parasites (WT). HCT-8 cells were infected with indicated transgenic strain, compound was added at the time of infection, and parasite survival was measured by NanoLuciferase after 48 hours of co-culture. Four parameter logistic non-linear regression plotted (GraphPad). EC_{50} values calculated for DDD489 (**A**) WT = 0.085 µM, KRS-OE = 0.978 µM, KRS-A309L= 8.63 µM and DDD508 (**B**) WT = 0.27 µM, KRS-OE = >1 µM, KRS-A309L = 9.93 µM. Mean ± SD (three technical replicates at each concentration); representative graph of three biological replicates shown. WT parasites are a transgenic reporter strain that expresses NanoLuciferase from a locus independent of *CpKRS*.

Supplementary tables

Table S1. ***C. parvum*** **potency, solubility, and permeability data for all fourteen tool compounds and DDD706.**

Structure (ID)	BCS Class[a]	MW[b]	*C. parvum* EC_{50} (µM)[c]	*C. parvum* EC_{90} (µM)[d]	Aqueous solubility (µM)[e]	PAMPA Papp (nm/s)[f]
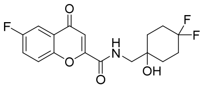 **(DDD706)**	1	355.3	1.46	3.01	185	63
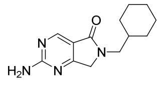 **(DDD212)**	1	246.3	0.17	0.35	232	131
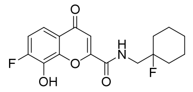 **(DDD993)**	1	337.3	0.10	0.15	234	63
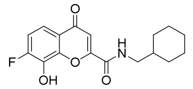 **(DDD714)**	1	319.3	0.03	0.05	377	43
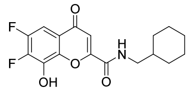 **(DDD690)**	1	337.3	0.19	0.37	226	32
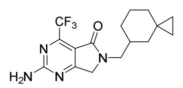 **(DDD909)**	2	340.3	0.07	0.13	14	174
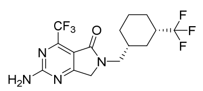 **(DDD489)**	2	382.3	0.04	0.10	21	171
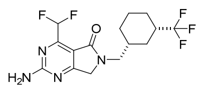 **(DDD352)**	2	364.3	0.02	0.06	32	193
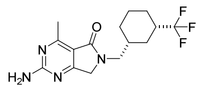 **(DDD478)**	2	328.3	0.02	0.04	57	138

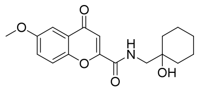 **(DDD583)**	2	331.4	0.14	0.33	33	115
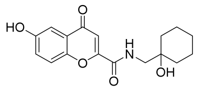 **(DDD508)**	3	317.4	0.13	0.38	184	6.9
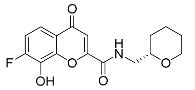 **(DDD695)**	3	321.3	0.08	0.18	236	11
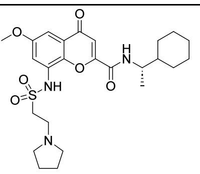 **(DDD582)**	3	505.6	0.15	0.37	225	16
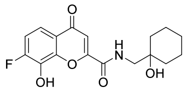 **(DDD844)**	4	335.3	0.17	0.46	63	1.7
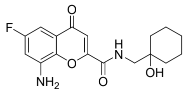 **(DDD229)**	4	334.3	0.03	0.08	1.9	19

[a]BCS is Biopharmaceutical Classification System. [b]MW is molecular weight. [c]Inhibition of *C. parvum* growth co-cultured with HCT-8 cells for 48 hrs. Data are means of at least 2 biological replicates. [d]*C. parvum* EC_{90}, calculated from the average EC_{50} and hill slope. [e]Aqueous solubility is kinetic solubility measured using UHPLC. [f]PAMPA is Parallel Artificial Membrane Permeability Assay; Papp is apparent permeability in nanometers/second. Solubility and permeability data are means of 2 technical replicates. Data for DDD706, an early lead *(8)* is included for comparison.

Table S2. ADME properties and *in vitro* assessment of toxicity/off-target effects for DDD489 and DDD508.

		DDD489	DDD508
A	FaSSIF solubility (mM)	48	325
	MDCK-MDR1 Papp A-B (nm/s)	514 (efflux ratio: 1.3)	159 (efflux ratio: 9.5)
	CHIlogD, clogP	2.8, 4.0	1.0, 2.0
	Mics CLi (m, b) (mL/min/g)	12, 25	1.4, < 0.5
	Heps CLi (m, r, d, h) (mL/min/g)	10, 1.9, 1.8, 1.1	3.7, 1.1, 1.2, < 0.5
	PPB (m, r, d, b, h) (% bound)	84, 58, 70, 74, 77	40, 53, 48, 47, 58
B	CYP450 IC_{50} (1A2, 2C19, 2C9, 2D6, 3A4) (mM)	40, >100, 32, 8, >100	> 100
	Mini-Ames (4 strains ± S9)	Negative	Negative
	hERG IC_{50} (mM)	> 30	> 30
C	Oxygen consumption AC_{50} (mM)	> 100	> 100
	Extracellular acidification rate AC_{50} (mM)	No response	No response
	Reserve capacity AC_{50} (mM)	No response	> 100
D	5-HT transporter (*h*) (% inhibition)	62	ND
	Na^{+} channel (site 2) (% inhibition)	50	ND

Data are from a single biological replicate. **(A)** *In vitro* ADME properties. FaSSIF is Fasted State Simulated Intestinal Fluid Solubility. MDCK-MDR1 is a Madin-Darby canine kidney cell line transfected with the MDR1 gene, encoding for the efflux protein, P-glycoprotein (P-gp). Papp is apparent permeability, measured in the presence of P-gp inhibitor, GF120918. Efflux ratio is calculated as the ratio of Papp B-A/A-B. CHIlogD is chromatographic hydrophobicity index logD, measured at pH 7.4. clogP is calculated logP, determined by Stardrop. Mics CLi is liver microsomal intrinsic clearance, calculated using microsomal protein yields of 48 mg protein/g liver (mouse) and 52.5 mg protein/g liver (bovine). Heps Cli is liver hepatocyte intrinsic clearance, calculated using scaling factors of 135 million hepatocytes/g liver (mouse), 108 million hepatocytes/g liver (rat), 170 million hepatocytes/g liver (dog), 117.5 million hepatocytes/g liver (human). PPB is plasma protein binding. Data quoted is percent bound. M=mouse, r=rat, d=dog, b=bovine, h=human. **(B)** *In vitro* toxicity assays. Cyp450 IC50 is cytochrome p450 enzyme inhibition. Mini-Ames was conducted in the presence and absence of S9 mix in four strains of *Salmonella typhimurium*. hERG IC_{50} is human ether-a-go-go related gene inhibition, measured using manual patch-clamp technique. **(C)** Mitochondrial toxicity assay data. **(D)** In a safety panel of receptors and enzymes (44 screens at 10 µM) DDD489 displayed significant inhibition (> 50%) in two assays. ND is not determined.

Table S3. Cross-species PK data for DDD489 and DDD508.

	DDD489						DDD508			
	Mouse		Rat		Dog		Mouse		Rat	
	PO	IV	PO	IV	PO	IV	PO	IV	PO	IV
**Dose (mg/kg)**	10	3	10	2.8	10	3	10	3	10	3.47
**AUC (µg-min/mL)**	31	137	8.2	72	40	60	16	89	22	54
**C_{max} (µg/mL)**	0.2		0.03		0.2		0.2		0.1	
**T_{max} (h)**	1		2		1.2		0.25		1	
**F (%)**	7		3.2		18		6		14	
**CL_B (mL/min/kg)**		22		39		52		34		64
**T½ (h)**		1.5		1.2		1.7		0.6		0.6
**Vd_{ss} (L/kg)**		2.8		3.2		4.2		1.8		2.1

PK data following a single oral or intravenous dose of DDD489 or DDD508 to female Balb/C mice (3 mice per group), male Han Wistar rats (3 rats per group), or male beagles (3 dogs per group). AUC is the area under the concentration time curve until the last measurement. C_{max} is the maximum concentration reached. T_{max} is the time at which the maximum concentration was reached. F% is oral bioavailability. CL_B is clearance in blood. T½ is half-life. Vd_{ss} is volume of distribution at steady state.

Table S4. Calf clinical scoring.

Observations	Score			
	**0**	**1**	**2**	**3**
**Fecal consistency (diarrhea)**	Normal	Mild	Moderate	Severe
**Demeanor**	Normal	Mild depression	Moderate depression	Severe depression
**Body condition**	Normal	Thin	Emaciated	N/A
**Dehydration (skin tent)**	< 2 seconds	2-6 seconds	> 6 seconds	N/A
**Appetite**	Normal	Reduced	Poor	Inappetent

Observations recorded at least once daily for duration of experiment. Staff performing scoring were blind to treatment.

Table S5: Oligonucleotides used in this work

Purpose	Name	Sequence (5'-3')
Mouse efficacy studies	Cp18s forward	TAGAGATTGGAGGTTGTTCCT
	Cp18s reverse	CTCCACCAACTAAGAACGGCC
Calf efficacy study	18S-JVA-F1	ATGACGGGTAACGGGGAAT
	18s-JVA-R1	CCAATTACAAAACCAAAAAGTCC
	18s-JVA-probe	[FAM]CGCGCCTGCTGCCTTCCTTAGATG[BHQ1]
DNA Sequencing CpKRS	KRS ORF F	GCATGGCATCTTAGAAGGATAATGTC
	KRS ORF R	GCGGTGGTTTCTCAACAACT

Table S6. Data collection and refinement statistics for *Cp*KRS complexes.

	**DDD489**	**DDD508**
**Beamline**	ESRF ID23-1	Diamond I03
**Detector**	DECTRIS PILATUS 6M	DECTRIS EIGER2 XE 16M
**Wavelength (Å)**	0.97625	0.97627
**Space group**	$P2_1$	$P22_12_1$
**Unit cell lengths (*a, b, c* Å)**	73.10, 118.67, 142.84	72.99, 121.76, 143.09
**Unit cell angles (a,b,g °)**	90, 90.38, 90	90, 90, 90
**Resolution range (Å)**	47.61-2.40 (2.44-2.40)	56.02 – 2.00 (2.04 – 2.00)
**Total no. of reflections**	306713 (15720)	1195340 (64489)
**Total unique reflections**	91915 (4619)	86869 (4559)
**Redundancy**	3.3 (3.4)	13.8 (14.1)
**Completeness**	96.7 997.6)	100 (100)
R_{sym}	0.108 (0.767)	0.223 (2.516)
R_{pim}	0.102 (0.706)	0.090 (1.002)
**<I>/<s>**	8.6 (1.6)	8.7 (1.2)
$CC_{1/2}$	0.992 (0.809)	0.997 (0.494)
R_{work} / R_{free}	22.57%/ 26.30%	18.16%/ 22.19%
**B-factors**		
**Protein (A/B/C/D)**	35.2/40.7/33.9/35.1	33.6/38.2/-/-
**Ligand (A/B/C/D)**	22.2/29.1/29.8/21.7	26.7/30.1/-/-
**Lysine (A/B/C/D)**	26.8/27.0/30.3/21.7	24.3/29.9/-/-
**Waters**	26.2	39.5
**R.m.s. deviations**		
**Bond lengths (Å)**	0.0065	0.0071
**Bond angles (°)**	1.1918	1.3375
**Ramachandran favoured / allowed / disallowed (%, all chains)**	98 / 2 / 0	99/1/0
**PDB code**	8R2A	8S00

Data measurement statistics for the highest resolution shell are shown in parentheses.

The following data files are available online:

Data file S1. Differential Expression Analysis of *Cryptosporidium parvum* Genes.

The table (attached as csv file) presents a summary of the differential expression analysis results. Each row corresponds to a unique gene characterized by the following attributes: “Gene_acc”: a unique integer identifier. “Gene_id”: The gene identifier from CryptoDB. “logFC”: The logarithm (base 2) of the fold change in TMT intensity between the WT (Wilde Type) and OE (OverExpression) samples as computed by the limma R package. “log_AveExpr”: The logarithm (base 2) of the average gene expression level across all samples as computed by the limma R package. “FDR”: The False Discovery Rate-adjusted p-value. Desc: The gene description from CryptoDB. “WT_1” to “WT_4”: The MaxQuant quantified TMT intensity levels for each of wild-type replicates, normalized and fed to the limma package. “OE_1” to “OE_4”: TheMaxQuant quantified TMT intensity levels for each of the overexpression condition, normalized and fed to the limma R package. For enhanced visualization and exploratory data analysis, the table has been formatted to be compatible with the interactive plotting tool available at https://plothub.pages.dev/. This online resource provides an intuitive graphical representation of the data, allowing for immediate visual assessment and interpretation.

Data file S2. Raw data from figures.
